# Supplementary material for: Sites of high local frustration in DNA origami
Source: Nat Commun. 2019 Mar 5;10:1061. doi: 10.1038/s41467-019-09002-6 (PMC6400978; doi:10.1038/s41467-019-09002-6)
Supplement: Supplementary file 1 — Supplementary Information [file 41467_2019_9002_MOESM1_ESM.pdf]

Sites of high local frustration in DNA origami

Richard Kosinski,[1] Ann Mukhortava,[2] Wolfgang Pfeifer,[1] Andrea Candelli,[2] Philipp Rauch,[2] and Barbara Saccà[1]*

[1]ZMB, University of Duisburg-Essen, Universitätstr. 2, 45117 Essen, Germany
[2]LUMICKS, De Boelelaan 1085, 1081 HV Amsterdam, The Netherland
*corresponding author. Email: barbara.sacca@uni-due.de

Supplementary Information

This document includes:

- Supplementary Figures 1 – 46
- Supplementary Tables 1 – 10
- Supplementary Note 1
- Supplementary Methods
- Supplementary References

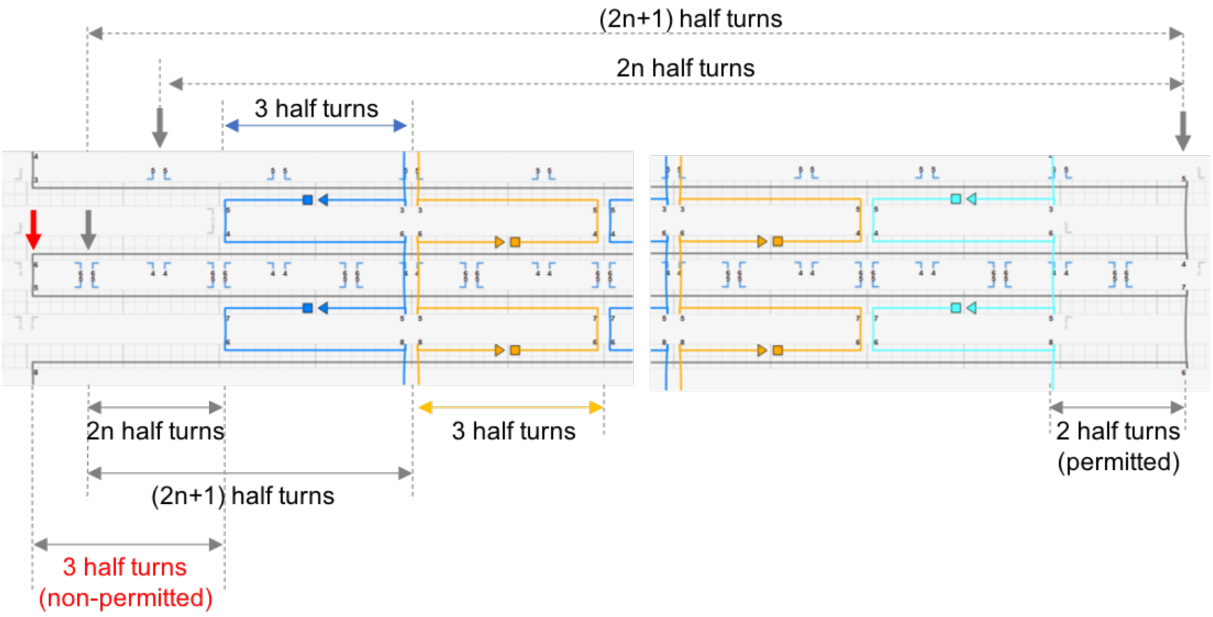

Supplementary Figure 1. Canonical two-dimensional (2D) origami design according to Rothemund´s rules. The design relies on the regular positioning of staple crossovers every 16 bp (i.e. 3 half helical turns) on alternating sides of the same helix. Scaffold crossovers are instead created by inversion of the scaffold strand when filling the raster pattern back and forth from one helix to another. To minimize the strain at the inversion points, scaffold crossovers between consecutive helices should be spaced by an odd number (2n + 1) of half turns (an even number of half turns is instead required for scaffold crossovers belonging to the same helix, as for example in presence of a central seam). In our design, each DNA origami is seamless and contains two sets of scaffold crossovers, one on the right side and one on the left side. Whereas the former is topologically permitted, the latter imposes a high topological stress, because it is positioned a half-helical turn away from the ideal location.

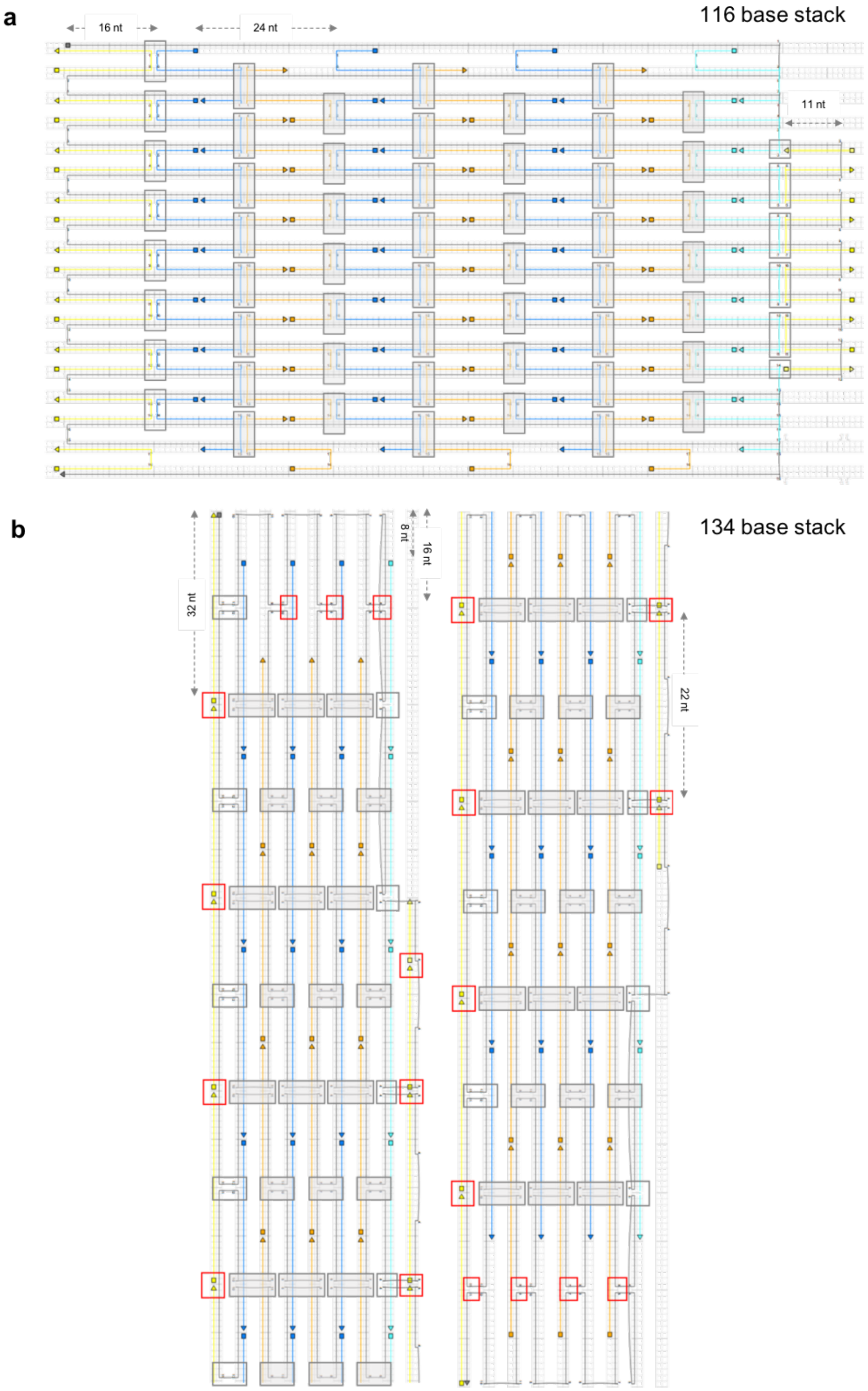

Supplementary Figure 2. Schematic representation of the *iso* I and *iso* II isomers. Each domain is constituted by a central core of 45 HJs and two sets of edges, respectively, on the left/right and bottom/up side of the shape. The core HJs (grey squares) of the *iso* I and *iso* II forms display isomerized base-stacking interactions at the crossover. The two shapes have approximately the same surface area, but a different aspect ratio, with *iso* I about 43 nm x 53 nm and *iso* II ca. 87 x 23 nm (the small discrepancy in the areas calculated as length x width derives from the fact that the domains are not perfectly rectangular in shape). Notably, whereas in the *iso* I form the scaffold runs back and forth performing 18 turns per domain, the *iso* II form displays about 140 turns per domain. The different geometric path of the scaffold in *iso* II, although being topologically disfavored by the high level of inner curvature, leads to formation of additional base stacking interactions between the 5'-end of one edge staple and the 3'-end of the adjacent one (red squares), resulting in 18 new stacked double-helical segments.

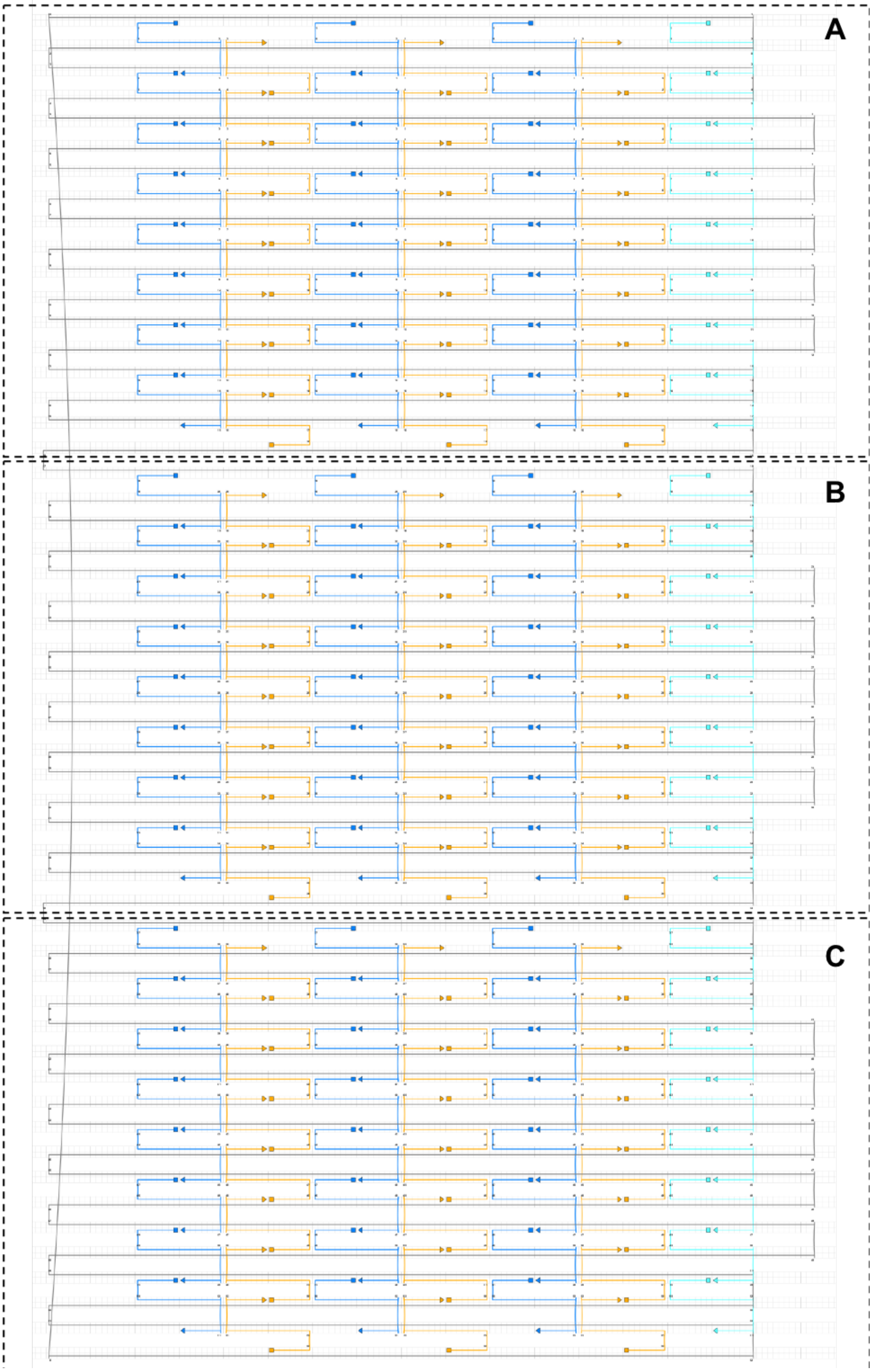

Supplementary Figure 3. Schematic representation of the e(-) construct in the *iso* I form. The full DNA origami structure is composed of three domains (A, B and C), that are identical in topology but different in sequence content. The core of each domain is kept in place by 56 staples, the last 8 of which (cyan) also define a portion of the right-side edge of the structure. The core staples are constituted by 8-16-8 nt-long stretches, hybridized to 2414 bases of the scaffold, thus employing the entire 7249-nt long M13 template for folding the full origami, leaving 2 or 3 bases of unpaired scaffold between consecutive domains. Design e(-) lacks the left and right side staples at the edges.

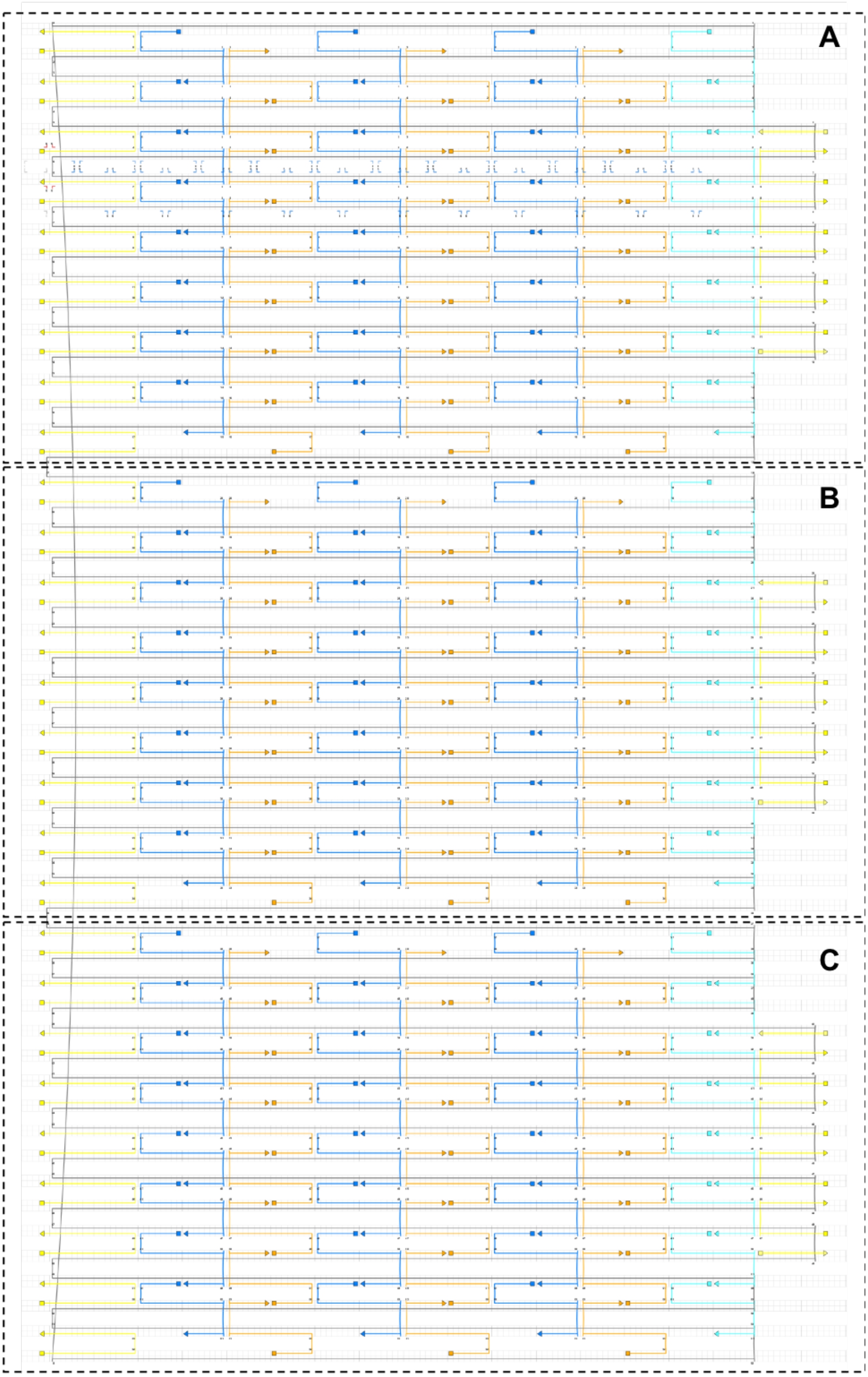

Supplementary Figure 4. Schematic representation of the e(0) construct in the *iso* I form. Adding the edges type 0 to the left and right side of the e(-) construct leads to the e(0) design. Edges type 0 (yellow strands) hybridize to distant regions of the scaffold forming a U-shape, with 2x16 nt (left-side) and 2x11nt (right-side) long segments.

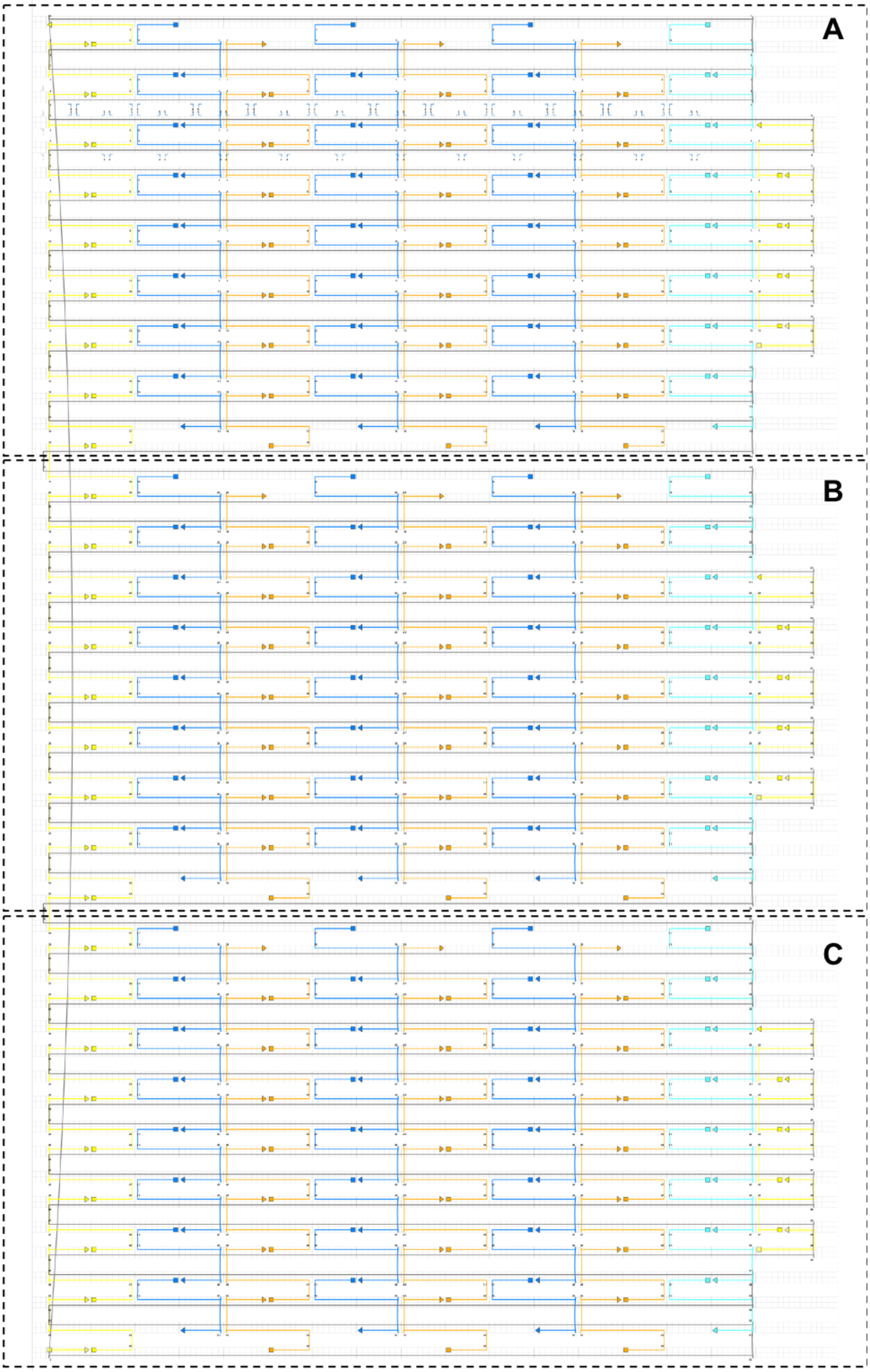

Supplementary Figure 5. Schematic representation of the e(1) construct in the *iso* I form. Adding the edges type 1 to the left and right side of the e(-) construct leads to the e(1) design. Edges type 1 (yellow strands) hybridize to three regions of the scaffold forming an S-shape, with 8-16-8 nt long segments (left side) and 5-11-6 nt-long segments (right side). They also comprise a T5 loop located over the scaffold turn.

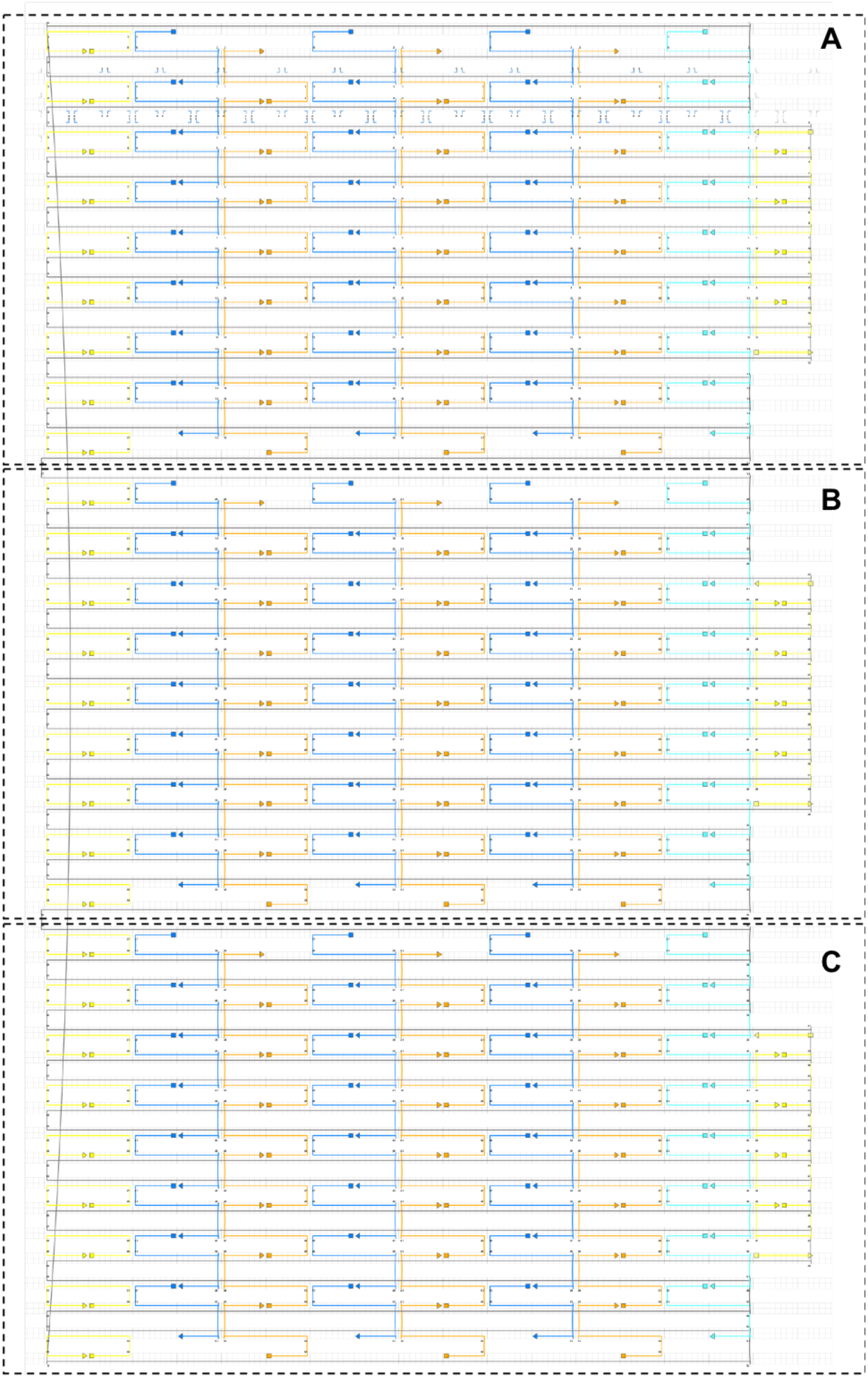

Supplementary Figure 6. Schematic representation of the e(2) construct in the *iso* I form. Adding the edges type 2 to the left and right side of the e(-) construct leads to the e(2) design. Edges type 2 (yellow strands) hybridize to three regions of the scaffold in an O-shape, with 8-16-8 nt long segments (left side) and 5-11-6 nt-long segments (right side). They also comprise a T5 loop placed between two adjacent scaffold turns.

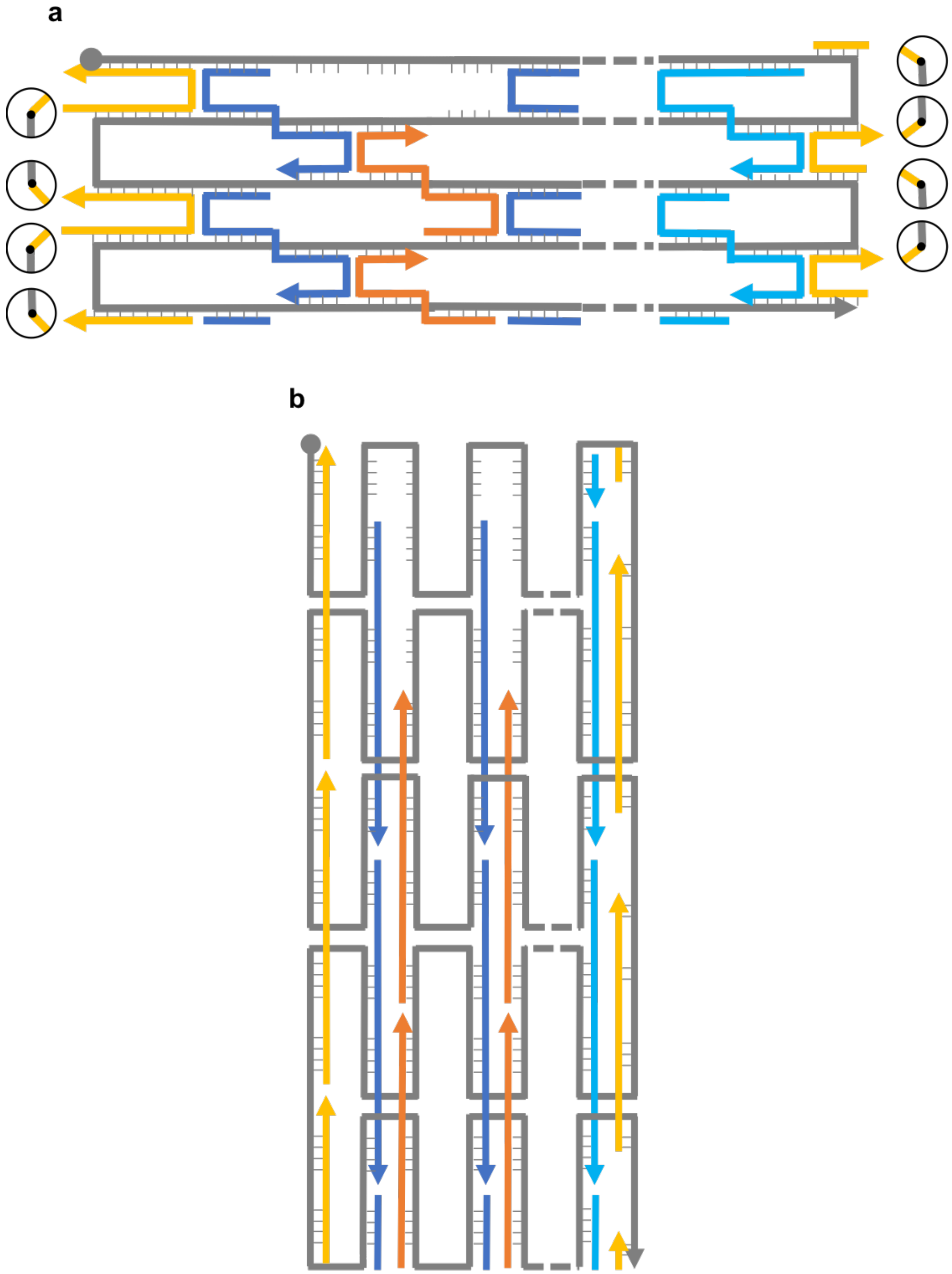

Supplementary Figure 7. Close view of the e(0) design in the *iso* I and *iso* II form. The scaffold travels from left to right and back in the *iso* I isomer (**a**), whereas adopts a more densely folded configuration in the *iso* II isomer, running from top to bottom and back several times (**b**). The structure is kept in place by staple crossovers of the core (blue and orange). The presence/absence of the last row of staples on the right side (cyan) and at the edges (yellow) affects the *iso* I/*iso* II ratio. The relative orientations of the nucleobase pairs at the edges of the structure are indicated by segments inscribed into a circle. The circle represents the DNA double helix, the segments represent the bases' orientations (yellow and grey, for the bases belonging, respectively, to the staple strand and the scaffold).

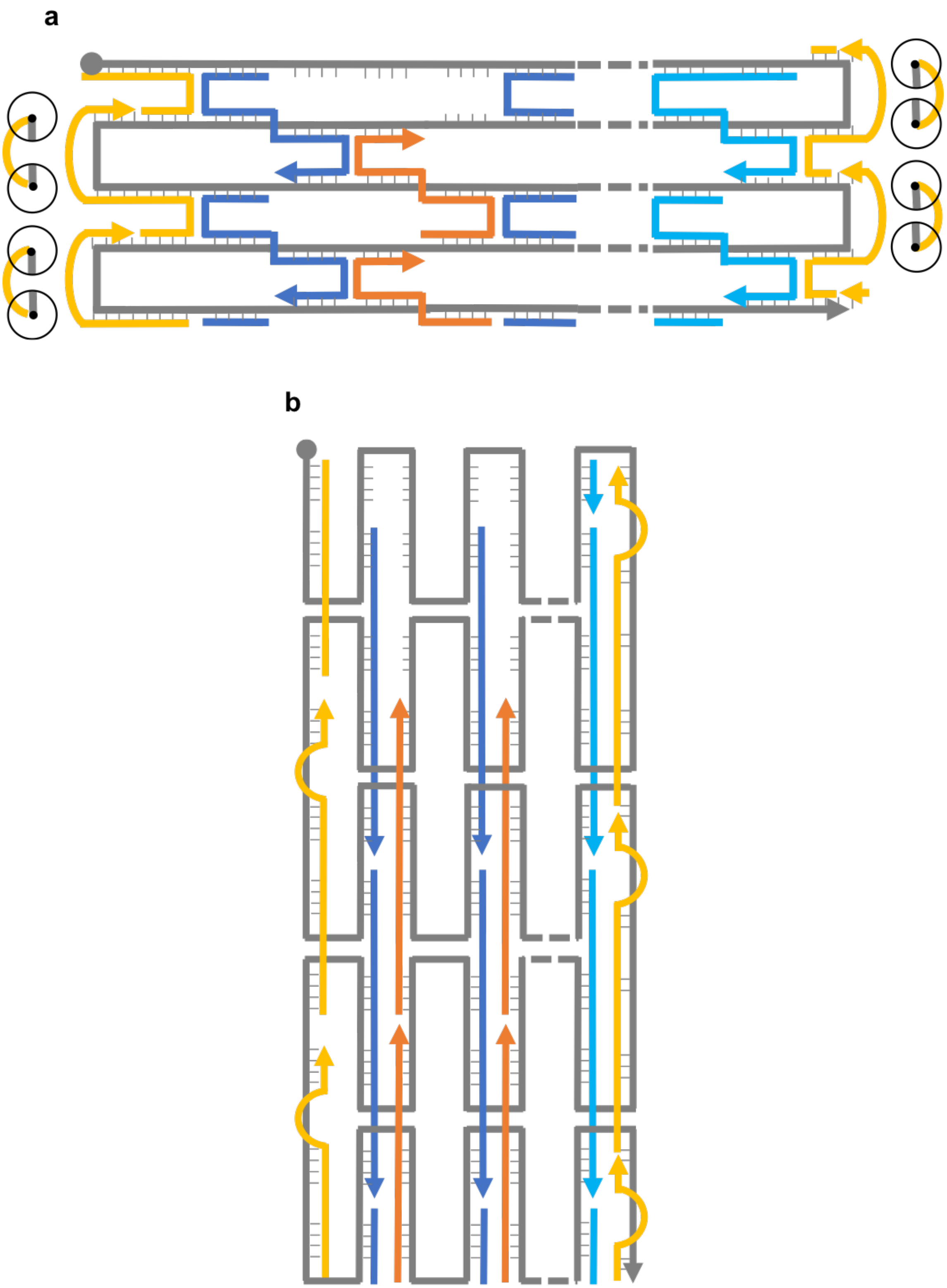

Supplementary Figure 8. Close view of the e(1) design in the *iso* I and *iso* II form. Description as in Supplementary Fig. 7. Note the T5 loops of the edges (curved yellow segments) traveling over the scaffold turn.

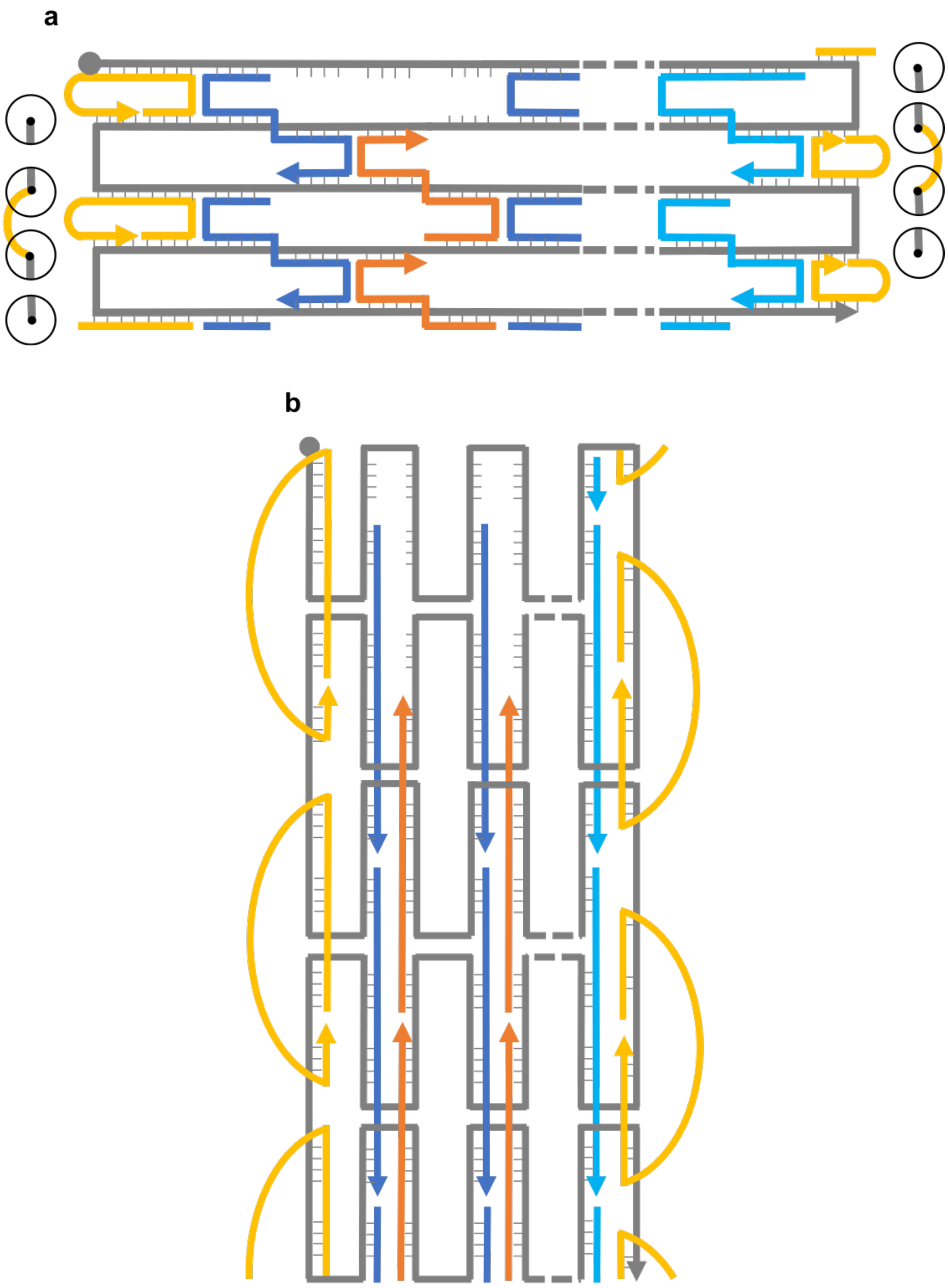

Supplementary Figure 9. Close view of the e(2) design in the *iso* I and *iso* II form. Description as in Supplementary Fig. 7. Note the T5 loops of the edges (curved yellow segments) traveling between adjacent scaffold turns. Circularization of the staples at the edges may hamper formation of the *iso* I species and prohibits formation of the *iso* II form. Release of mechanical stress can be thus achieved through formation of dimers as illustrated in Suppl. Fig. 15.

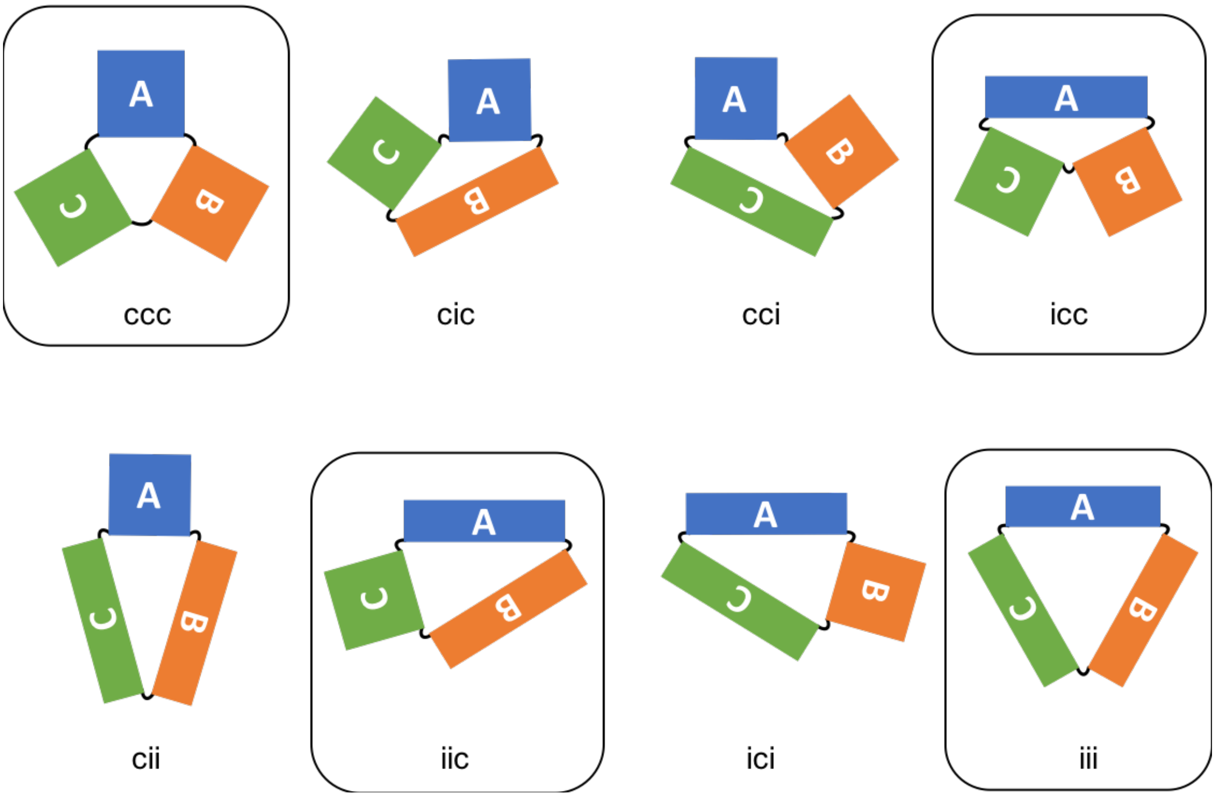

Supplementary Figure 10. All possible isomers of the full DNA origami structure. Considering that each of the three domains can assume one of two shapes, a canonical *iso* I (named as "*c*" and indicated by a square) or an isomerized *iso* II (named as "*i*" and indicated by a rectangle), the full structure can therefore appear in one of the eight possible permutated isomers. Studies performed on individually assembled domains enabled to reveal the preferential formation of four of the eight isomers (highlighted in black squares), namely, when reading from A to B to C, the isomers *ccc*, *icc*, *iic* and *iii*.

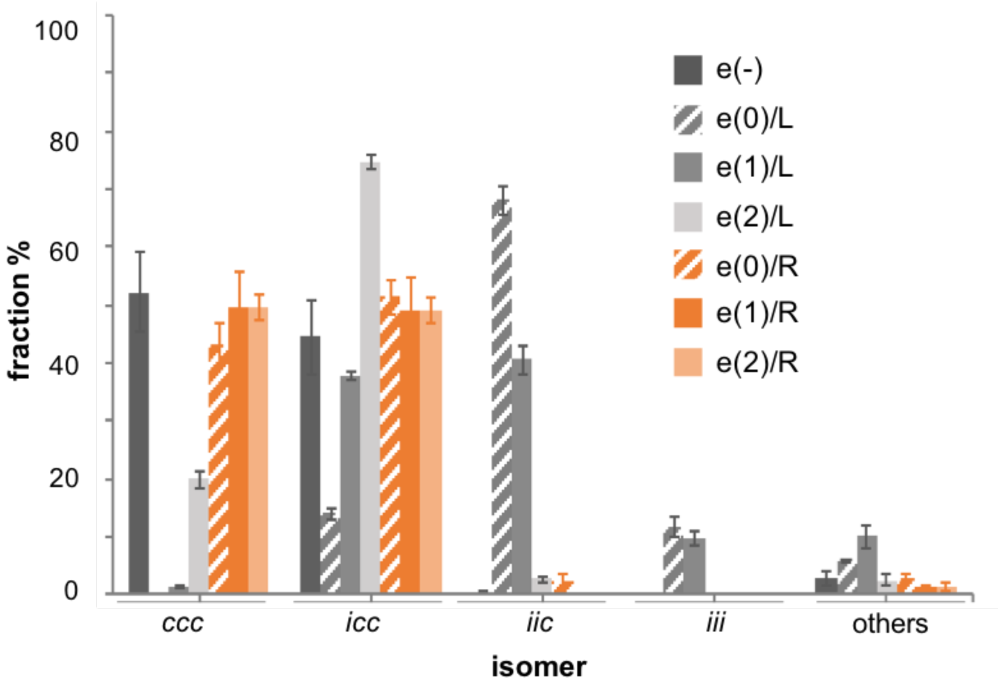

Supplementary Figure 11. Statistical AFM analysis of the full DNA origami structure in absence or presence of left- or right-side edges. The three domains were assembled in one-pot reaction in absence of any edges (e(-) construct), resulting in ca. 50% of the full shapes with one of the three domains in the *iso* II form (*icc*). Identical reaction mixtures were prepared and assembled in one-pot together with the staples that target either the left (L) or the right (R) side of each domain. Three sets of edges were used, either type 0, 1 or 2. Structures visualized at the AFM were counted manually (numerical values are given in Suppl. Table 1) and standard deviations were obtained from three different images from at least two distinct experiments). Assembly conditions were 2 nM scaffold and 50X staples in 1X TEMg at a cooling rate of -1°C/min. One can notice that, whereas the left-side edges can be used to trigger a substantial increase in the isomerization degree of the structure (light grey bars), the right-side edges (orange bars) do not lead to an appreciable change from the initial e(-) design (cfr. dark grey and orange bars). Error bars were obtained from three independent images of the same sample, each showing several hundreds of individual structures. Source data are provided as a Source Data file.

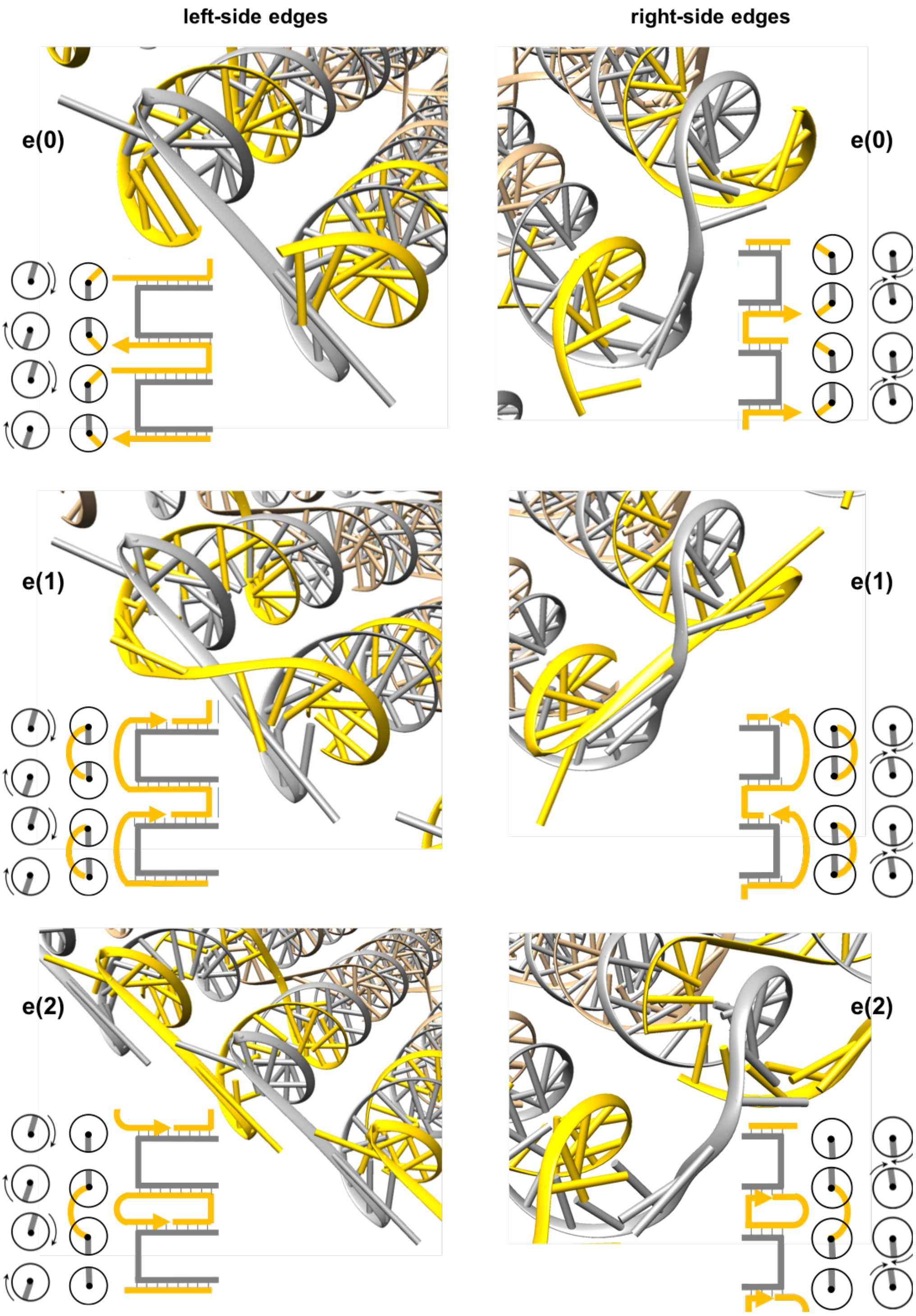

Supplementary Figure 12. Close-up view of the atomic models of the left- and right-side edges of the origami domain. Structural models were generated using the Atomic Model Generator tool supplied by the CanDo platform[1] and viewed in Chimera. The topology of the edges is depicted according to the original notation used by Rothemund and coworkers[2] (double helix: circular symbol; scaffold bases at the inversion point: grey segments; hybridized edge bases at the inversion point: yellow segments). Outer symbols indicate the orientation of the terminal scaffold bases in absence of any constraint, i.e. assuming formation of a B-DNA duplex. Hybridization of the unpaired scaffold loops with edge staples imposes a certain degree of torsional stress, that is proportional to the deviation from the ideal face-to-face orientation of the terminal scaffold bases. One can note that in all three cases analysed, binding of the edge staples on the right-side of the structure can be better accommodated when compared to the left-side. The insertion of T5 loops at the turning points of e(1) and e(2) staples (not indicated in the models) may also contribute to release torsional stress at the scaffold crossovers.

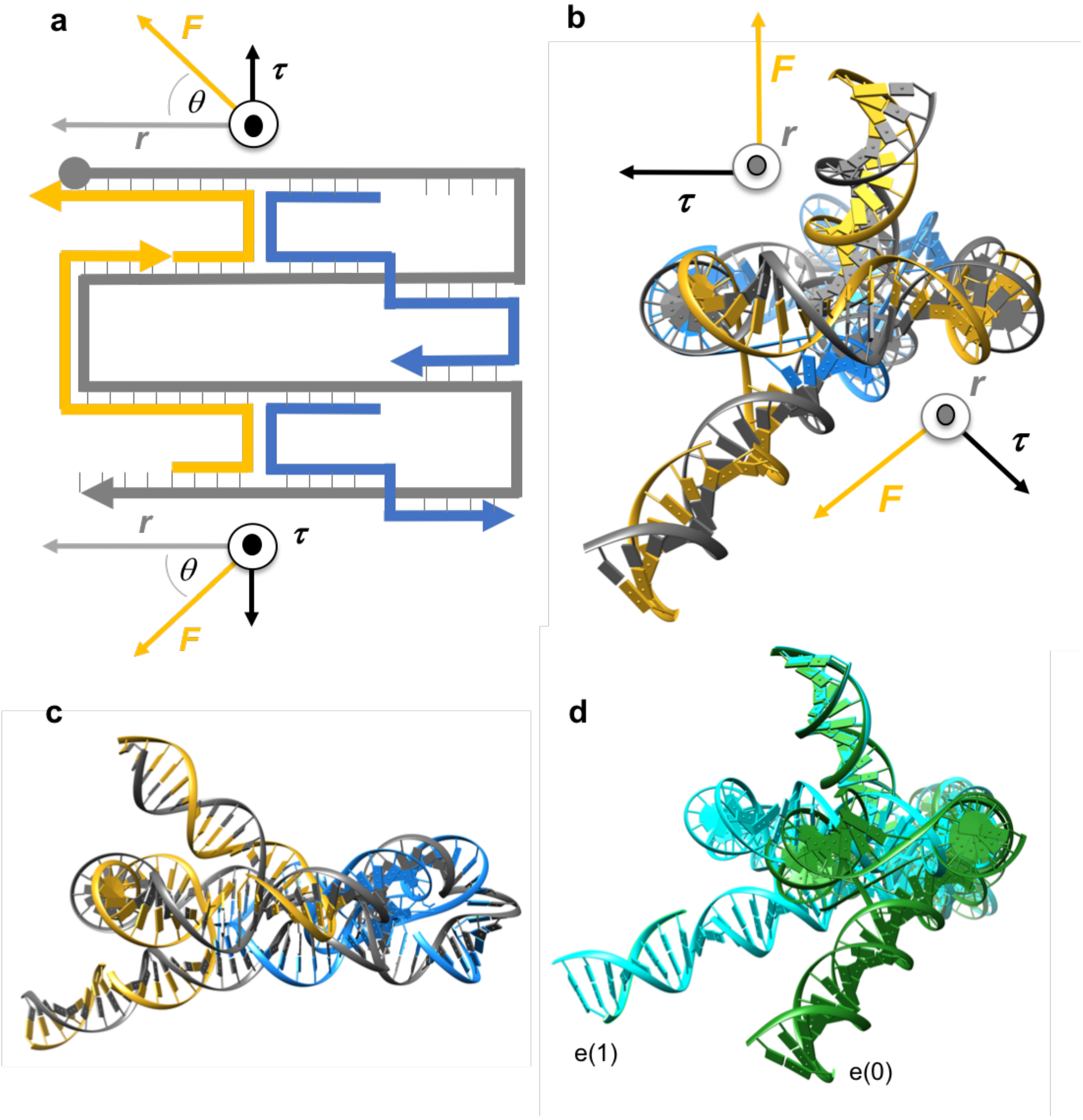

Supplementary Figure 13. Atomic model of the left-side edge of a minimal e(1) construct. Structural models were generated using the Atomic Model Generator tool supplied by the CanDo platform[1] and viewed in Chimera. Addition of edge staples of type 1 corresponds to the application of a force (***F***) to a lever whose arm length is equal to ***r*** (grey and yellow vectors) and applies at the crossover pivot. This generates two out-of-plane torques (black vectors, ***τ***), whose mechanical action drives the reconfiguration of the pattern of connected HJs into the *iso* II form. Schematic view of a minimal e(1) construct constituted by two vertically aligned HJs (**a**) and corresponding atomic model in lateral (**b**) and front (**c**) view. Overlap of the atomic models obtained for the e(0) and e(1) constructs (**d**) shows that the degree of torsional stress (i.e. the angular displacement between the two torque vectors) is larger in the e(0) design, in full agreement with our experimental observations.

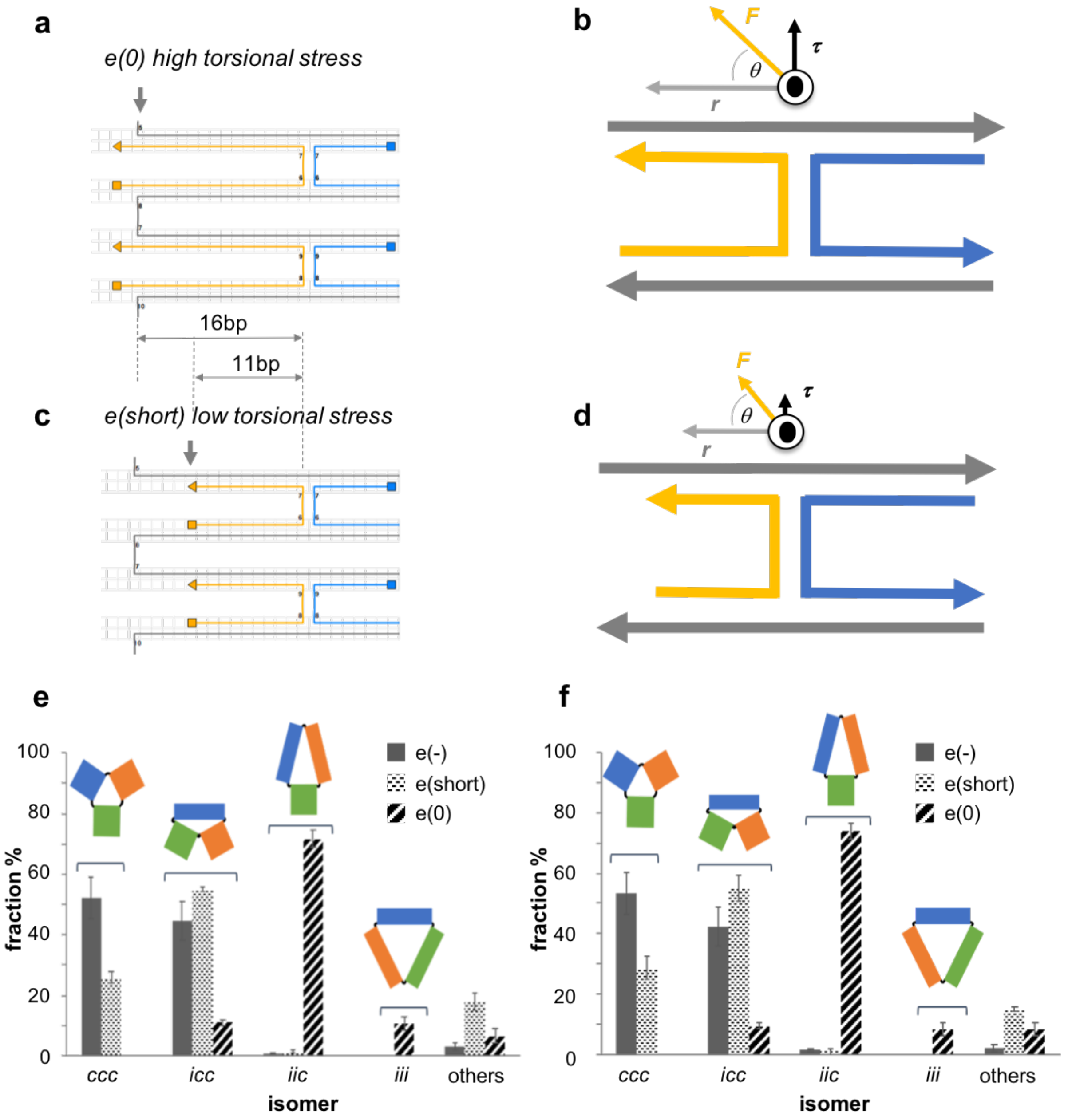

Supplementary Figure 14. Schematic representation of short e(0) staples and statistical analysis of the assembly products. According to our atomic model, the torque applied by the edge staples when binding to the scaffold is proportional to the length of the hybridizing segments and to their angular displacement (**a** and **b**). This latter is greater for terminal bases located far away from the "ideal" permitted position, as also schematically represented in Suppl. Fig 12. This hypothesis is confirmed by the lower degree of isomerization observed for edge staples of type 0 having a shorter length (**c** and **d**) and therefore terminating at an ideal scaffold position. AFM analysis of the end products of the e(short) design revealed indeed only a moderate increase in isomerized domains in respect to the e(-) construct, both at 2 nM (**e**) and 5 nM (**f**) scaffold concentration. Numerical data are given in Suppl. Table 3. Error bars were obtained from three independent images of the same sample, each showing several hundreds of individual structures. Source data are provided as a Source Data file.

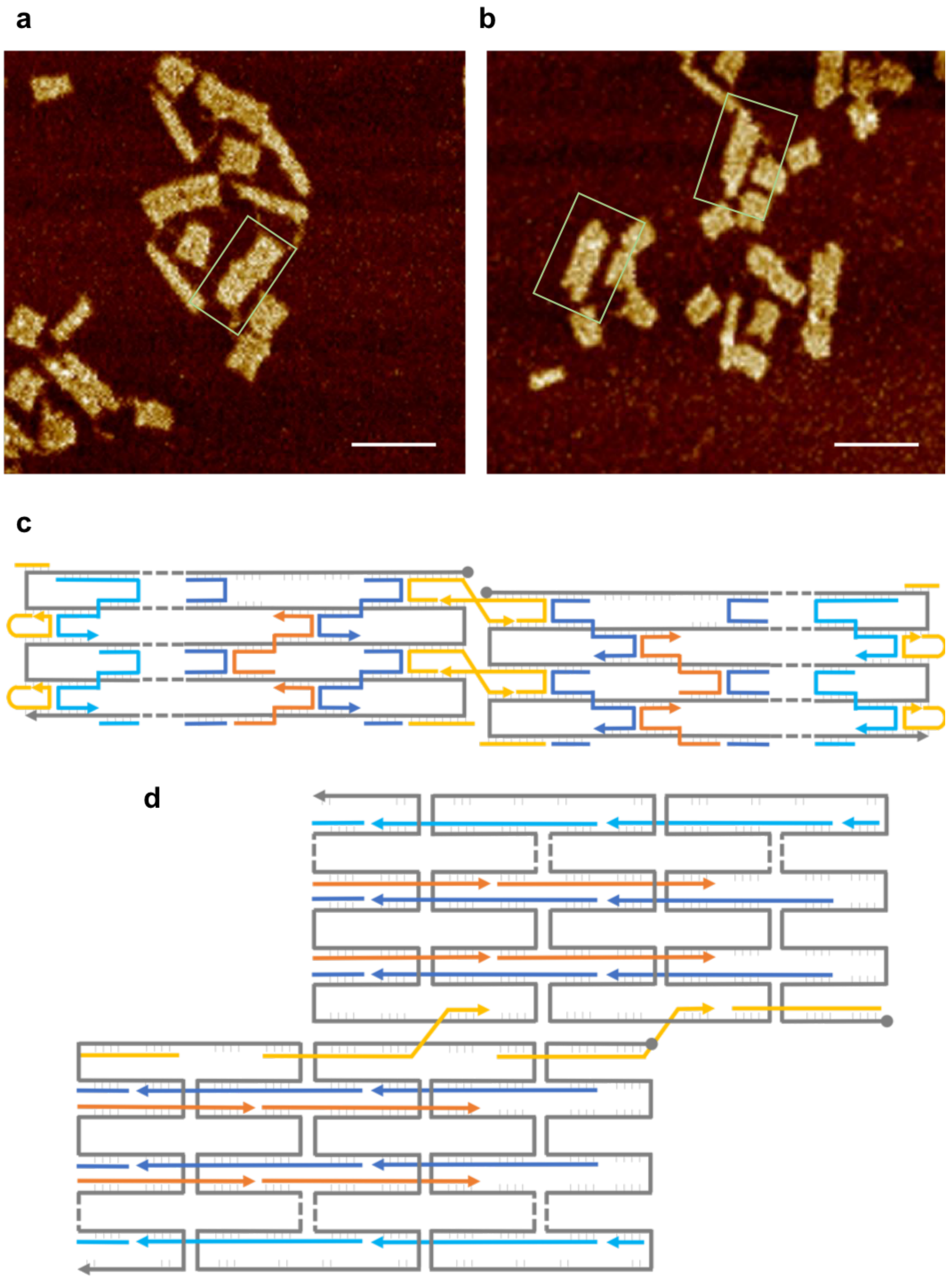

Supplementary Figure 15. Dimerization of e(2) constructs in the *iso* I and *iso* II forms. As shown in Suppl. Fig. 9, circularization of the edge staples of type 2 hampers formation of individual *iso* II isomers, forcing the structure to search for alternative ways to release the topological stress applied. A possibility, confirmed by the AFM images of the end products (**a** and **b**), is the formation of *iso* I or *iso* II dimers (**c** and **d**, respectively) through the bridging of edge staples between two distinct origami structures. This process is favoured by high sample concentrations, as showed in Suppl. Fig. 19 (cfr. e(2) images at standard assembly conditions and high concentrations). Scale bars are 100 nm.

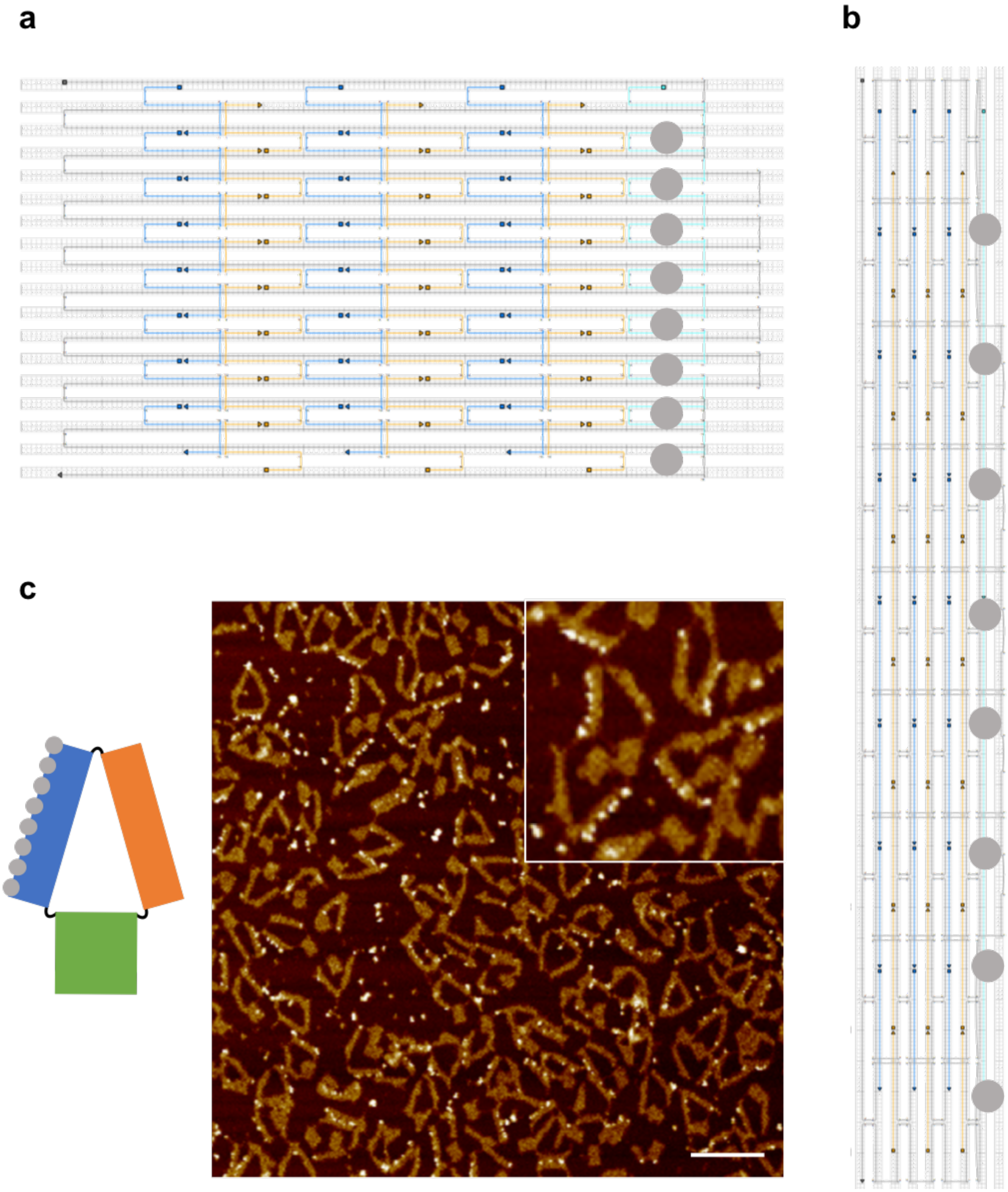

Supplementary Figure 16. Topographical mapping of the right-side edge of domain A. The last row of staples on the right side of domain A (cyan strands) has been marked with biotin molecules appended to their 3'-terminus. Addition of streptavidin (grey filled circles) should result in topographical mapping of these staples, enabling to prove their correct and full incorporation into the structure, either in the *iso* I (**a**) or *iso* II (**b**) conformation. AFM imaging of e(0) products of assembly (**c**) demonstrated successful incorporation of those staples in the *iso* II form, despite their partial deformation due to an adjacent scaffold loop of shorter length. Scale bar is 200 nm.

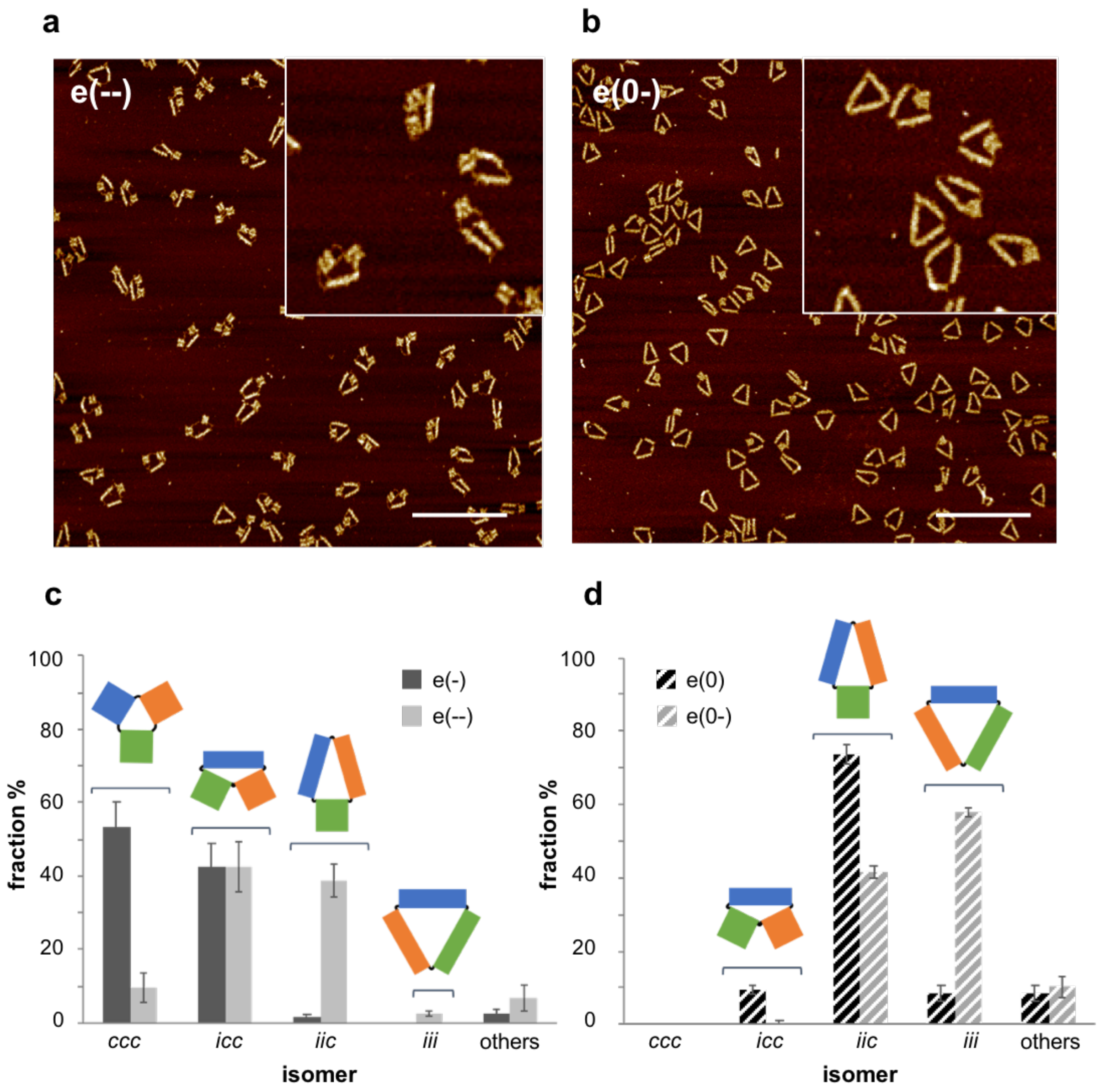

Supplementary Figure 17. Statistical AFM analysis of the end products of e(--) and e(0-) constructs. The full DNA origami was assembled in absence of the last raw of right-side staples (cyan strands in Suppl. Fig. 16). This led to an increased fraction of isomerized species, both in the absence (construct e(--)) or presence (constructs e(0-)) of the left-side edges of type 0. Representative AFM images of the two constructs (**a** and **b**, respectively) and comparison with the end products of e(-) and e(0), i.e. identical constructs which instead comprise the last raw of right-side staples (**c** and **d**, respectively). Numerical values of the AFM analysis are given in Suppl. Table 4. Assembly conditions were 5 nM scaffold at -1°C/min in 1X TEMg with 50X staple strands. Scale bars are 600 nm. Error bars were obtained from three independent images of the same sample, each showing several hundreds of individual structures. Source data are provided as a Source Data file.

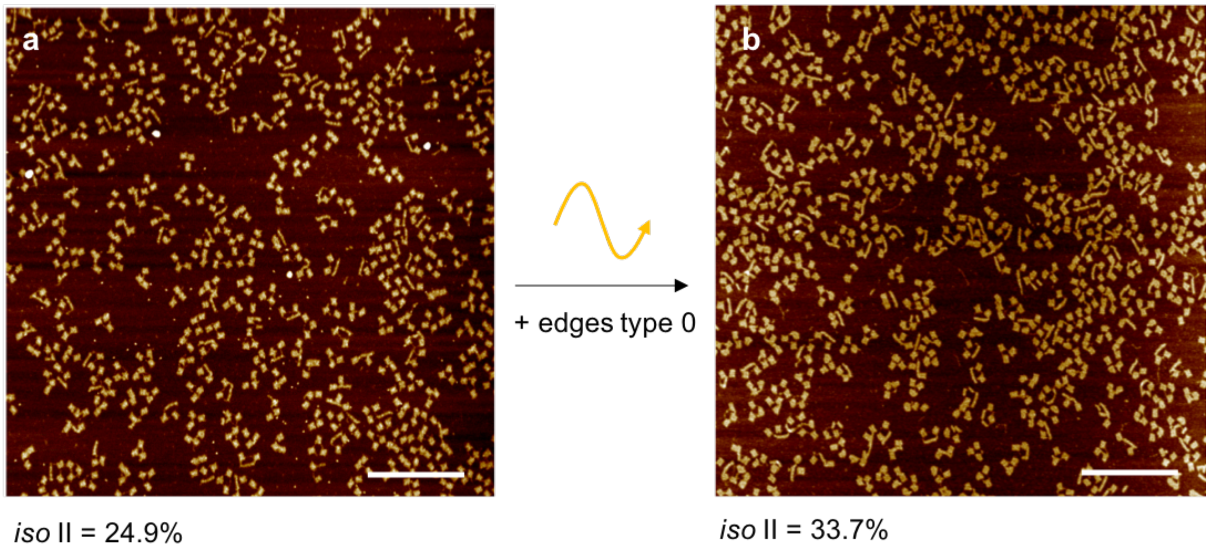

Supplementary Figure 18. Two-steps assembly of the domain A in the e(0) construct. Addition of edge staples of type 0 to a pre-assembled core of domain A (**a**) results in a minimal increase of isomerized species (**b**), going from a 24.9% of *iso* II domains in absence of edges to a 33.7% of *iso* II domains upon addition of edges type 0 for 1 hour at 30 °C. This suggests that effective incorporation of the edges occurs at higher temperatures, i.e. in an early stage of the assembly process. Assembly conditions were 2 nM scaffold at -1°C/min in 1X TEMg with 50X staple strands. Scale bars are 600 nm.

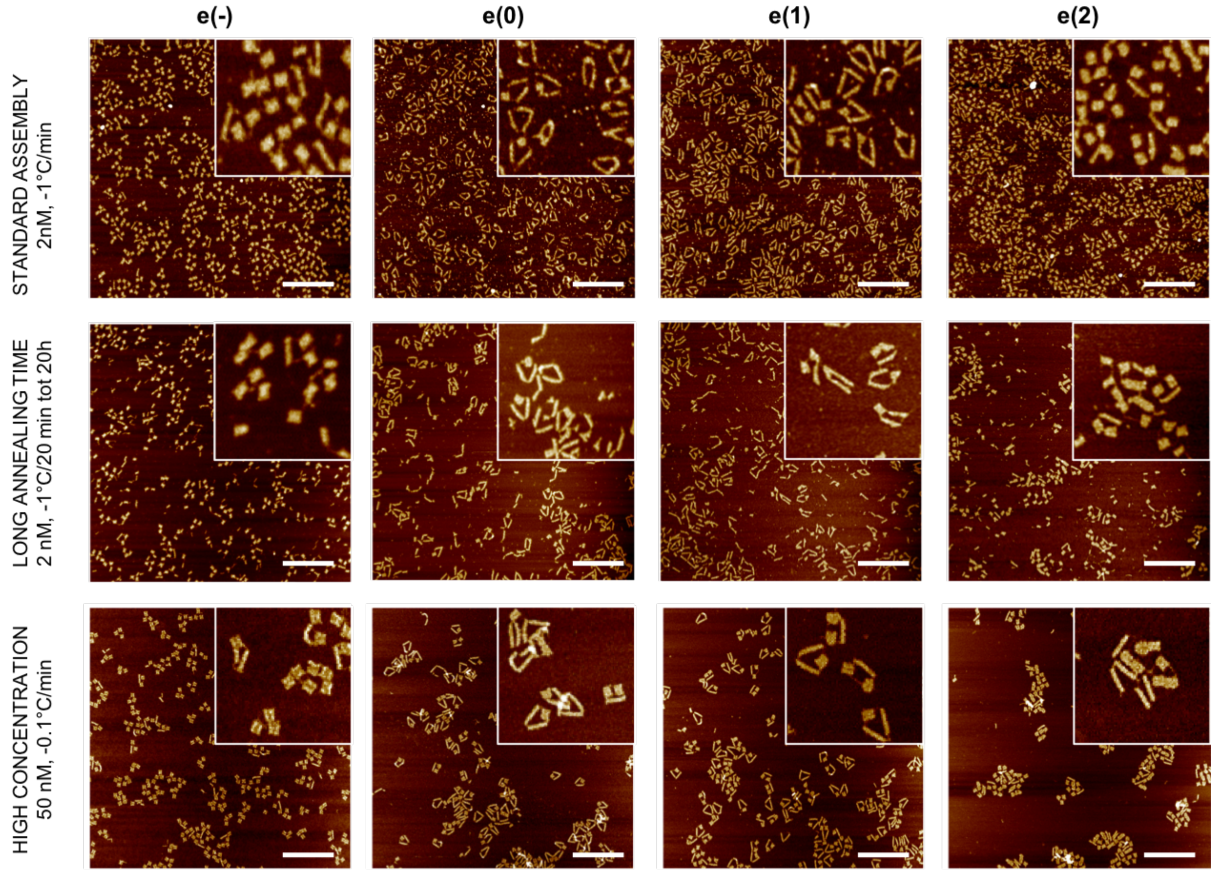

Supplementary Figure 19. Representative AFM images of the four constructs in different assembly conditions. Addition of edge staples of type 0, 1 or 2 (constructs e(0), e(1) and e(2), respectively) to the cores (construct e(-)) leads in all cases to a partial reconfiguration of one or more domains of the DNA origami structure. The extent of isomerization is largely dependent on the type of edges used. Increase of DNA concentration and/or annealing times (first and last raw, respectively) does not lead to a significant change in the isomerization rate of the domains. Contrarily, extremely slow annealing time (middle raw) result in accumulation of undefined structures and aggregates. Statistical analysis of the end products in all designs and conditions used is provided in Suppl. Fig. 20. Note the formation of e(2) dimers at high DNA concentrations (Suppl. Fig. 15). Scale bars are 600 nm. Insets are 1μm x 1μm.

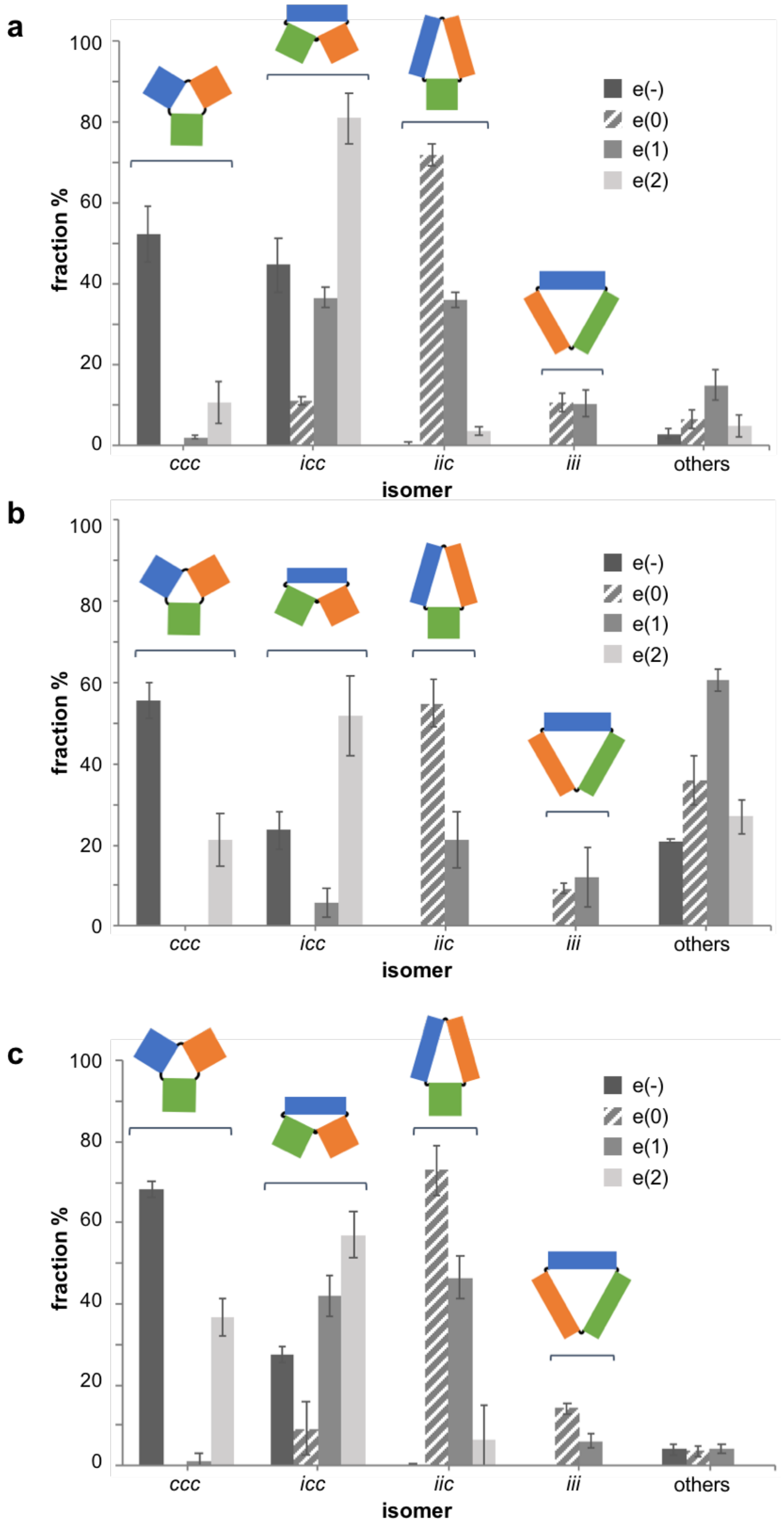

Supplementary Figure 20. Statistical AFM analysis of the full DNA origami structure in all designs and conditions used for assembly. The full DNA origami structure, either in absence (design e(-)) or presence of edges type 0, 1 or 2 (designs e(0), e(1) or e(2)) was assembled in different conditions, namely: 2 nM scaffold at -1°C/min (**a**), 2 nM scaffold at -1°C/20 min (**b**) or 50 nM scaffold at 0.1 °C/min (c). In all cases, the assembly buffer was 1X TEMg with 50X staple strands. Error bars were obtained from three independent images of the same sample, each showing several hundreds of individual structures. Source data are provided as a Source Data file.

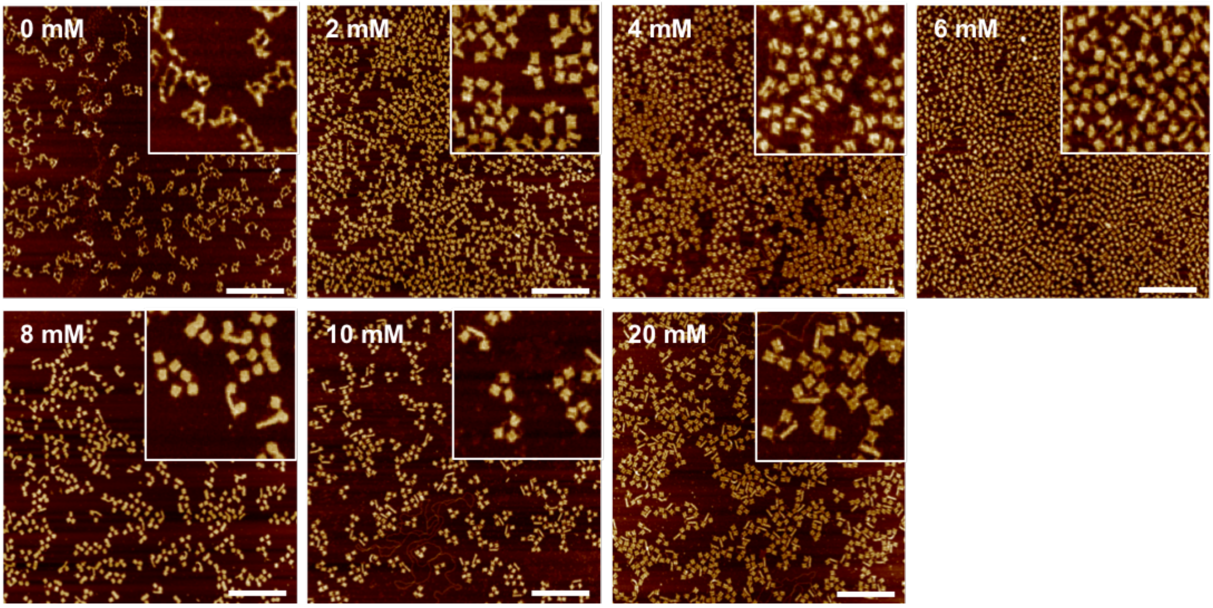

Supplementary Figure 21. Representative AFM images of the e(-) construct assembled in different magnesium ions concentrations. The full structure lacking the left- and right-sided edges (e(-) construct) was assembled in one-pot reaction mixture, using 1X TE buffer containing distinct concentrations of magnesium ions, from 2 mM to 20 mM. AFM analysis of the products obtained shows a direct correlation between the extent of isomerization and the increase in magnesium ions concentration. Statistical analysis of the end products is provided in Suppl. Table 6. Scale bars are 600 nm. Insets are 1μm x 1μm.

a

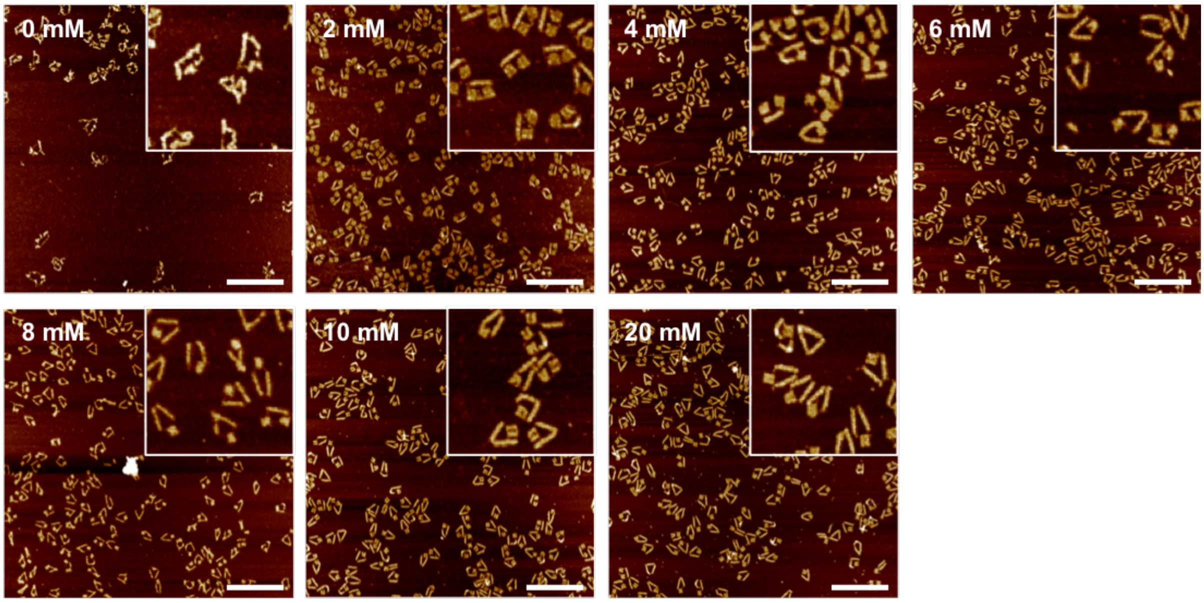

b

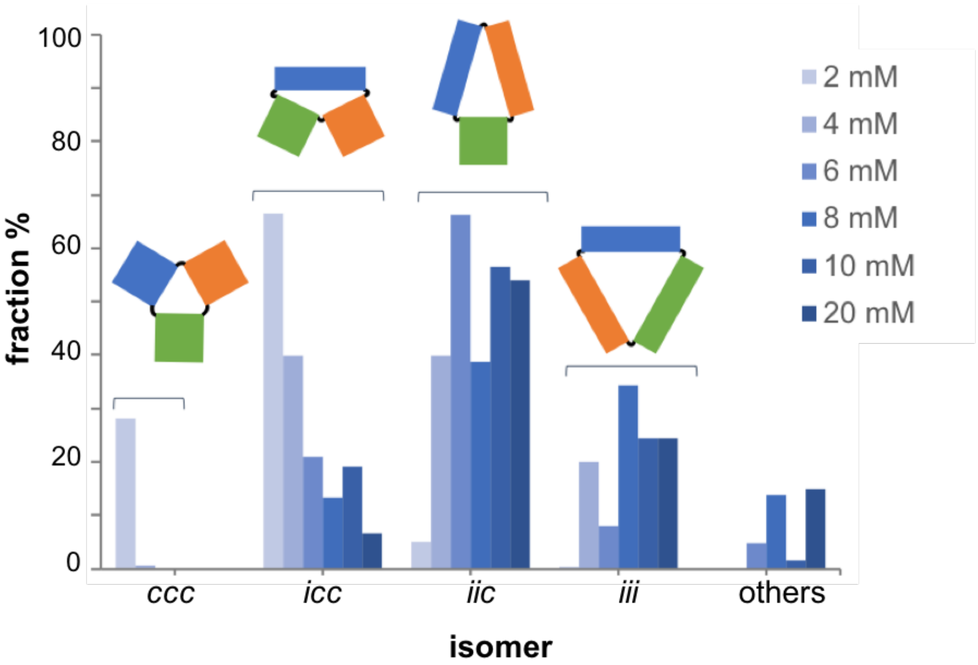

Supplementary Figure 22. Representative AFM images of the e(0) construct assembled in different magnesium ions concentrations. The full structure bearing both the left- and right-side edges of type 0 (e(0) construct) was assembled in one-pot reaction mixture, using 1X TE buffer containing distinct concentrations of magnesium ions, from 2 mM to 20 mM. (**a**) AFM analysis of the products obtained shows a tendential increase in the isomerization level with increasing magnesium ions concentration, although less evident than in the e(-) design (cfr. Suppl. Fig. 21). Scale bars are 600 nm. Insets are 1μm x 1μm. (**b**) Statistical analysis of the end products. Numerical values are provided in Suppl. Table 6. Source data are provided as a Source Data file.

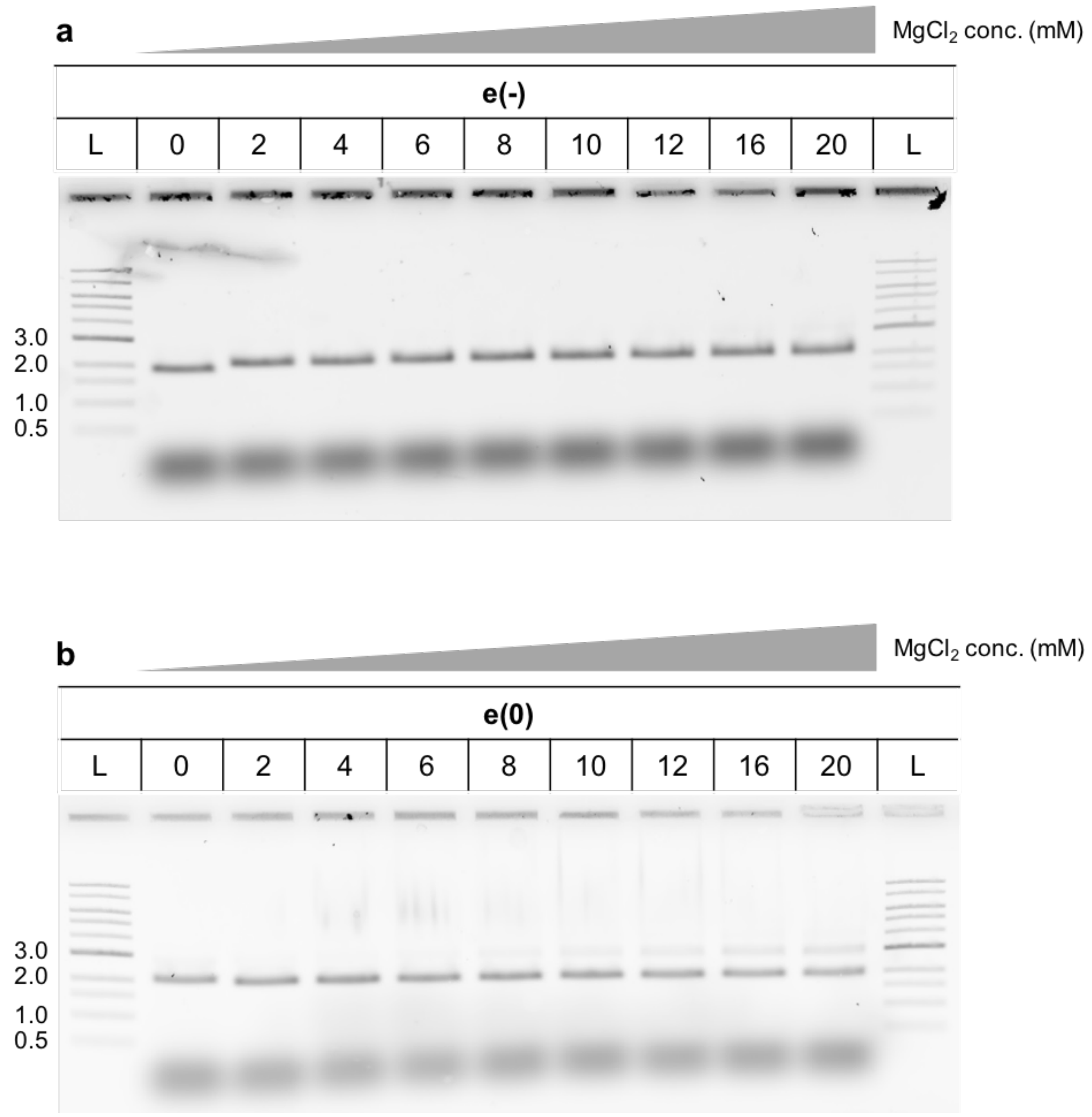

Supplementary Figure 23. Electrophoretic mobility assays of the e(-) and e(0) constructs at different magnesium ions concentrations. The full structure lacking or bearing the left- and right-side edges (construct e(-) or e(0), respectively, in **a** and **b**) was assembled in one-pot reaction mixture, using 1X TE buffer containing distinct concentrations of magnesium ions, from 0 mM to 20 mM. The end products of the assembly process were loaded on a 0.75% agarose gel (80 V, 2h at 4°C in 1X TBEMg buffer) and compared for their migration mobilities. The effect of Mg concentration on the yield of e(-) and e(0) structures appears to be negligible. The presence of the edges results into a slight increase in the migration rate of the main band, visible at Mg conc. > 0 mM (cfr. e.g. lane 2 in (a) and (b)). This might be explained by the increased number of base pairing in the e(0) design and a consequent slight increase in the molecular weight of the compound. Contrarily, the enhanced fraction of isomerized species for increasing magnesium concentrations does not seem to affect the migration rate of samples of the same edge-type (cfr. lanes 2 to 20 within each design). For this reason, the difference in migration rate between the two designs cannot be attributed to a different ratio of *iso* I/*iso* II species. Lane L contains a 1 kbp DNA ladder (Invitrogen). The DNA origami sample migrates between 2.0 and 3.0 kbp.

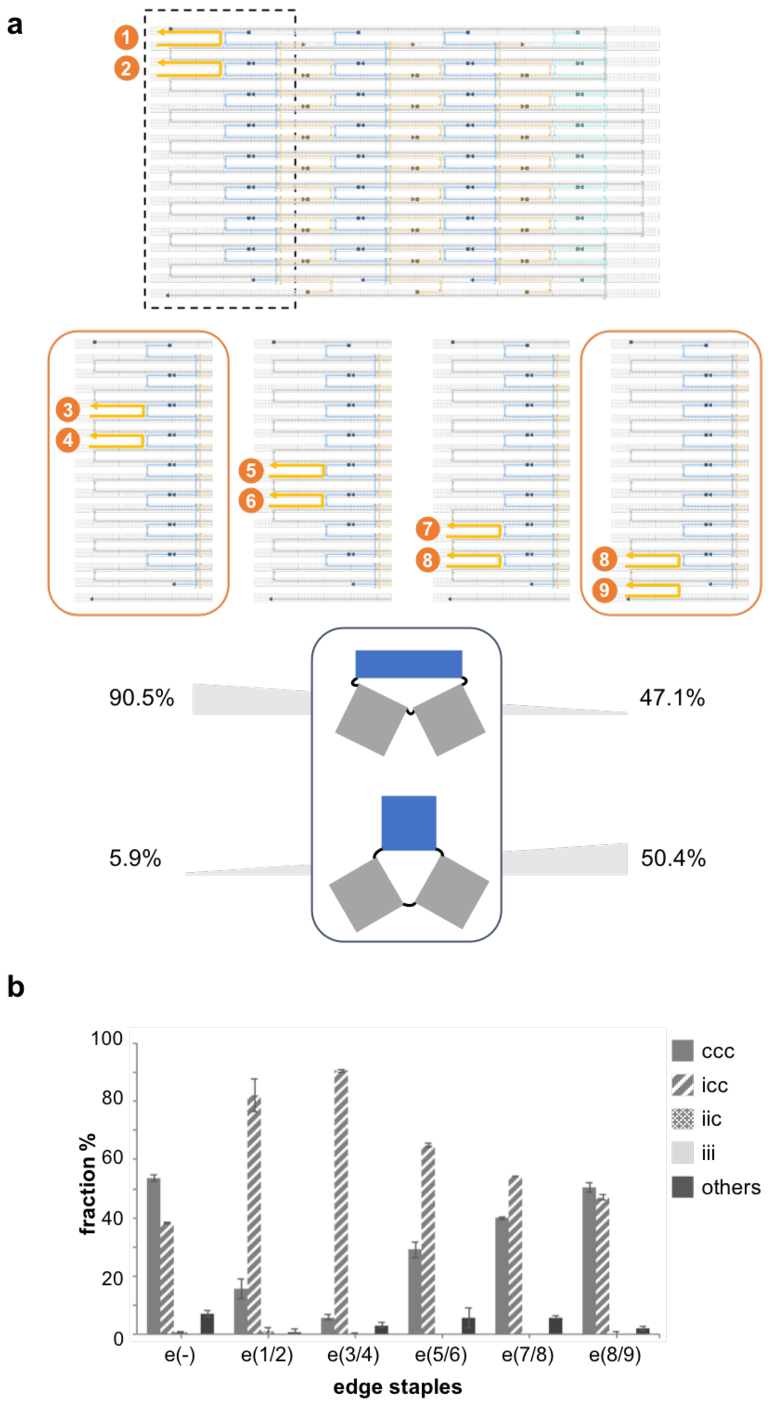

Supplementary Figure 24. Effect of individual triggers on the structural reconfiguration of domain A. The full structure was assembled in one-pot reaction in the absence of any edges (e(-) construct). Identical solutions were prepared where pairs of adjacent e(0) staples have been added, which target the left-side of domain A only. (**a**) Staple pairs are indicated as e(1/2), e(3/4), e(5/6), e(7/8) and e(8/9), and refer to the positions of the added staples on the left-side of domain A, from the top to the bottom of the structure. (**b**) The end products of the thermal assembly were visualized at the AFM and counted manually, revealing that edges (3/4) and (8/9) contribute to folding with opposite trends: whereas addition of edges (3/4) results in the largest isomerization fraction (90.5%; mostly represented by the *icc* isomer), addition of edges (8/9) has the smallest impact on isomerization, with about equal amounts of *iso* I and *iso* II isomers (47.1% and 50.4%, represented by the *icc* and *ccc* global shapes, respectively). Numerical values of the statistical analysis are given in Suppl. Table 7. Error bars were obtained from three independent images of the same sample, each showing several hundreds of individual structures. Source data are provided as a Source Data file.

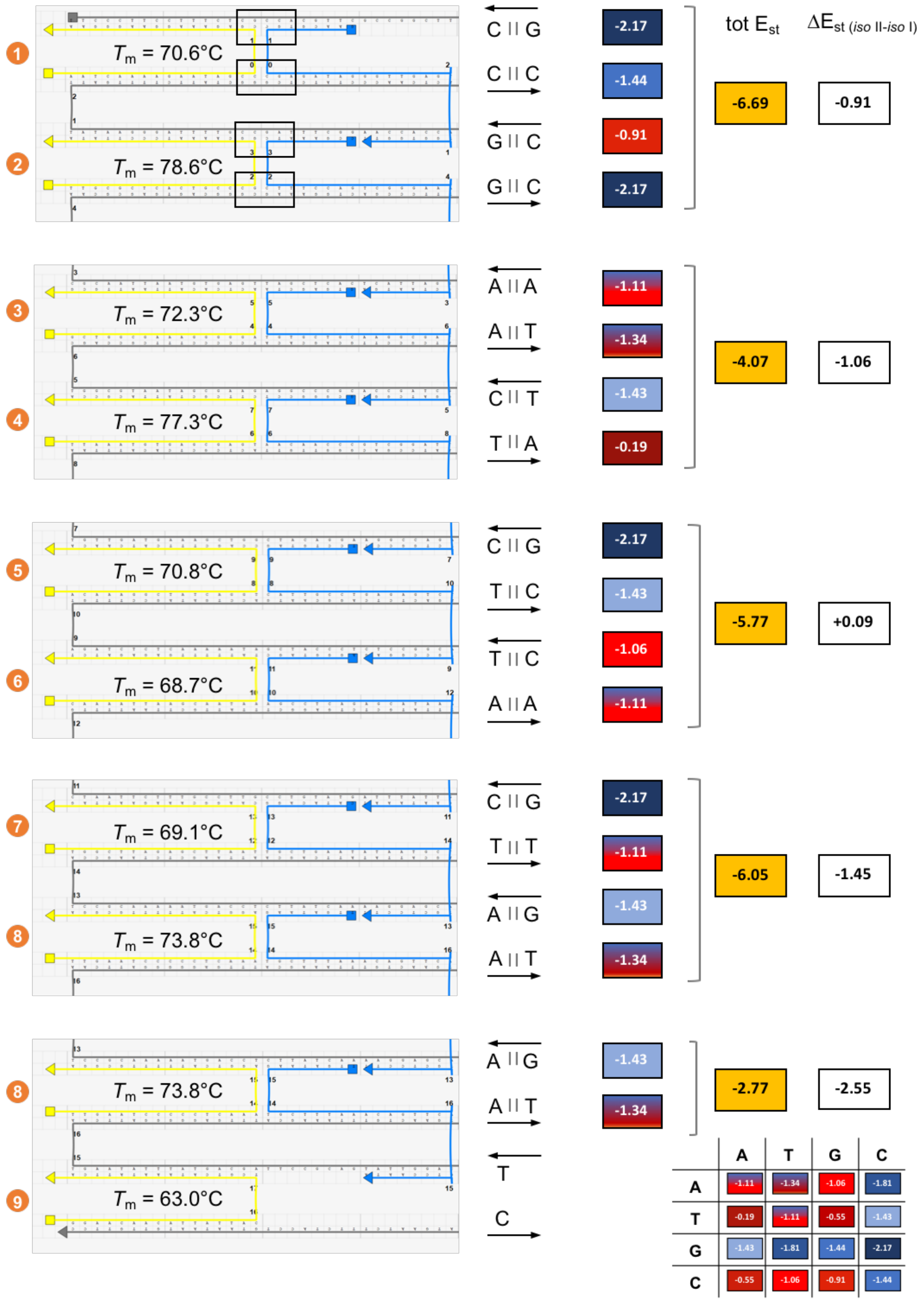

Supplementary Figure 25. Close-up view of the edge staples of domain A in the *iso* I conformer. Staples of type 0 (yellow strands, numbered from 1 to 9 when proceeding along the 5' to 3' direction of the scaffold path) have been mapped according to their melting temperature (calculated by Mfold[3] at 2nM DNA concentration, 12 mM magnesium and 10 mM sodium) and base stacking energies[4] at the crossover. The nucleobases at the crossover in the *iso* I conformer are indicated together with their color-coded energies (from blue to red for lower to higher values, i.e. from more to less stabilizing values). The total energies of base stacking (E_{st}) as well as the differences in base stacking contributions between the *iso* II (Suppl. Fig. 26) and the *iso* I conformers ($\Delta E_{st(iso\ II - iso\ I)}$) are also given (yellow and white boxes, respectively). Energy values are expressed in kcal/mol.

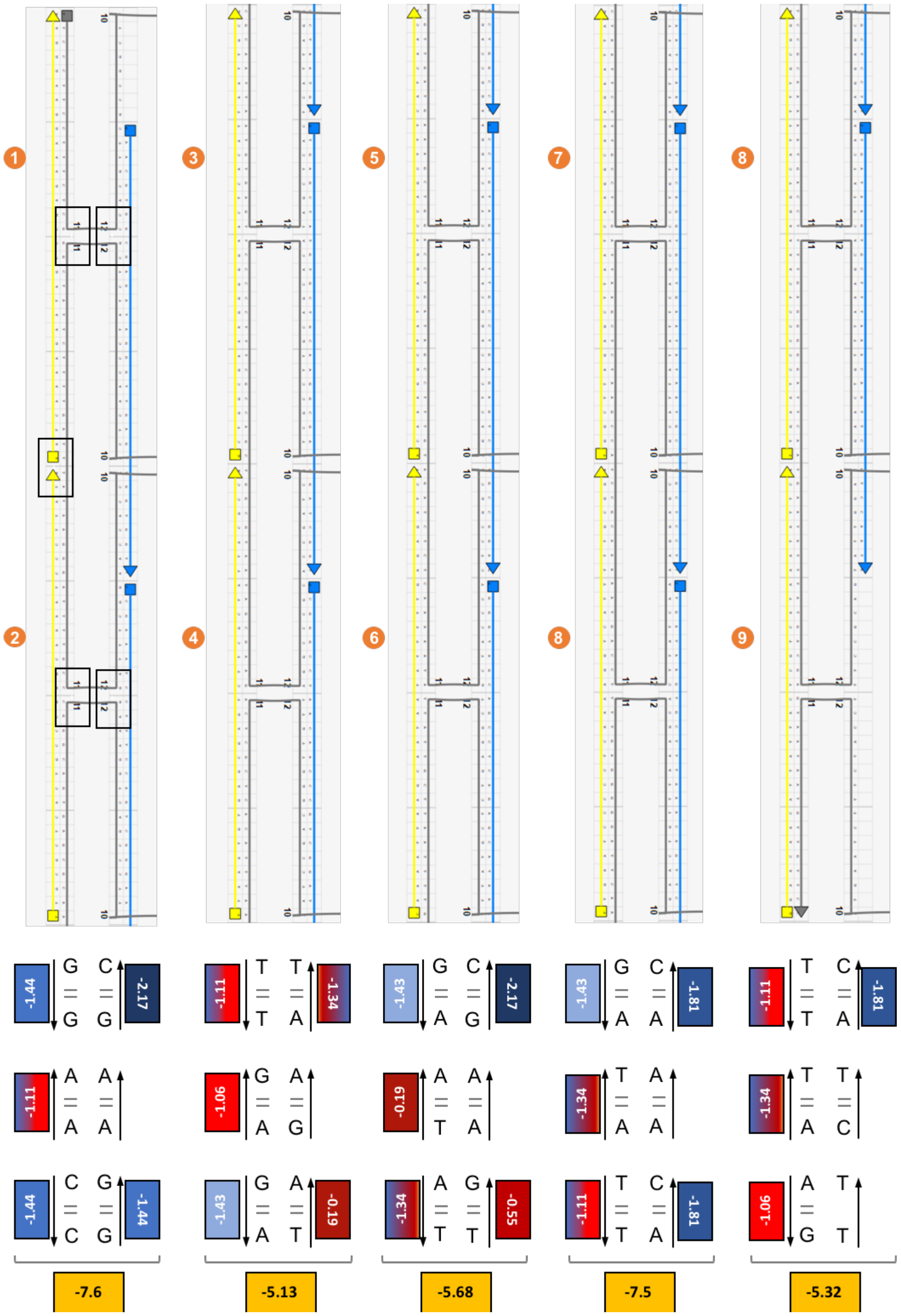

Supplementary Figure 26. Close-up view of the edge staples of domain A in the *iso* II conformer. Staples of type 0 (yellow strands, numbered from 1 to 9 when proceeding along the 5' to 3' direction of the scaffold path) are here represented in their *iso* II conformation. As the base sequences are identical in the two states, their melting temperatures keep identical, too. However, base stacking energies change, due to isomerization of the crossovers and establishment of additional stacking interactions between the terminal bases of two consecutive edge staples. The nucleobases at the crossover in the *iso* II conformer are indicated together with their color-coded energies (from blue to red for lower to higher values) and total energies of base stacking (yellow boxes). Energy values are expressed in kcal/mol.

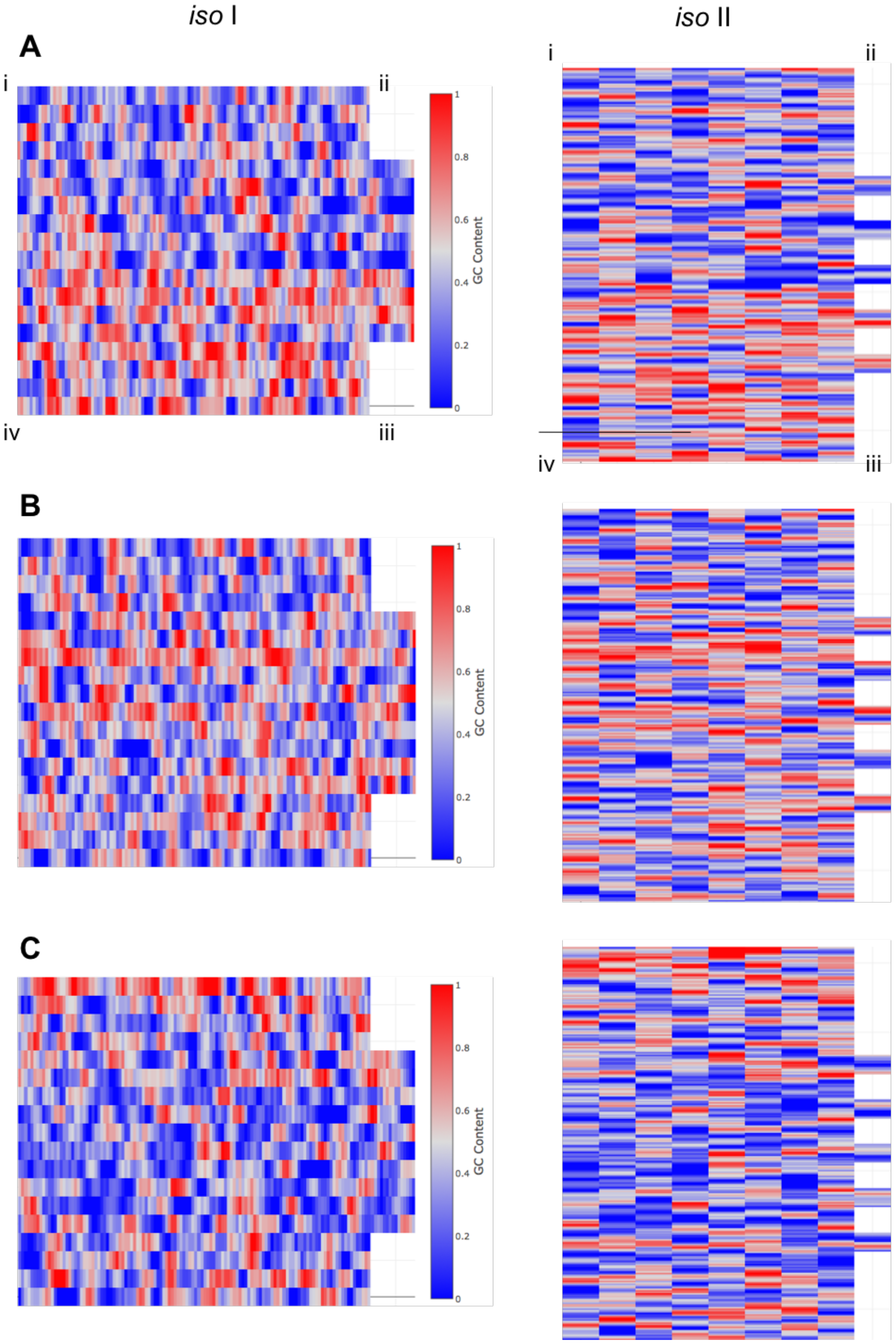

Supplementary Figure 27. Thermal GC maps of the individual domains in the two isomer forms. Each domain has been analyzed for its thermal stability (in terms of GC content)[5] in both the *iso* I and *iso* II shape, when edges type 0 are present in the design. Although horizontal "GC" pairs in *iso* I become vertically aligned in *iso* II, their spatial distribution within the structure is equal in the two shapes, going from the top left (i) to the bottom left corner (iv) and passing through (ii) and (iii). Thermal maps evidenced interesting differences between the domains, with A and B displaying high-T_m strands on the left-side and neighboring regions of the structure and C exhibiting instead a more homogeneous and lower thermal stability at the left-side edge, which may explain the minor "decisional" power of these strands during assembly.

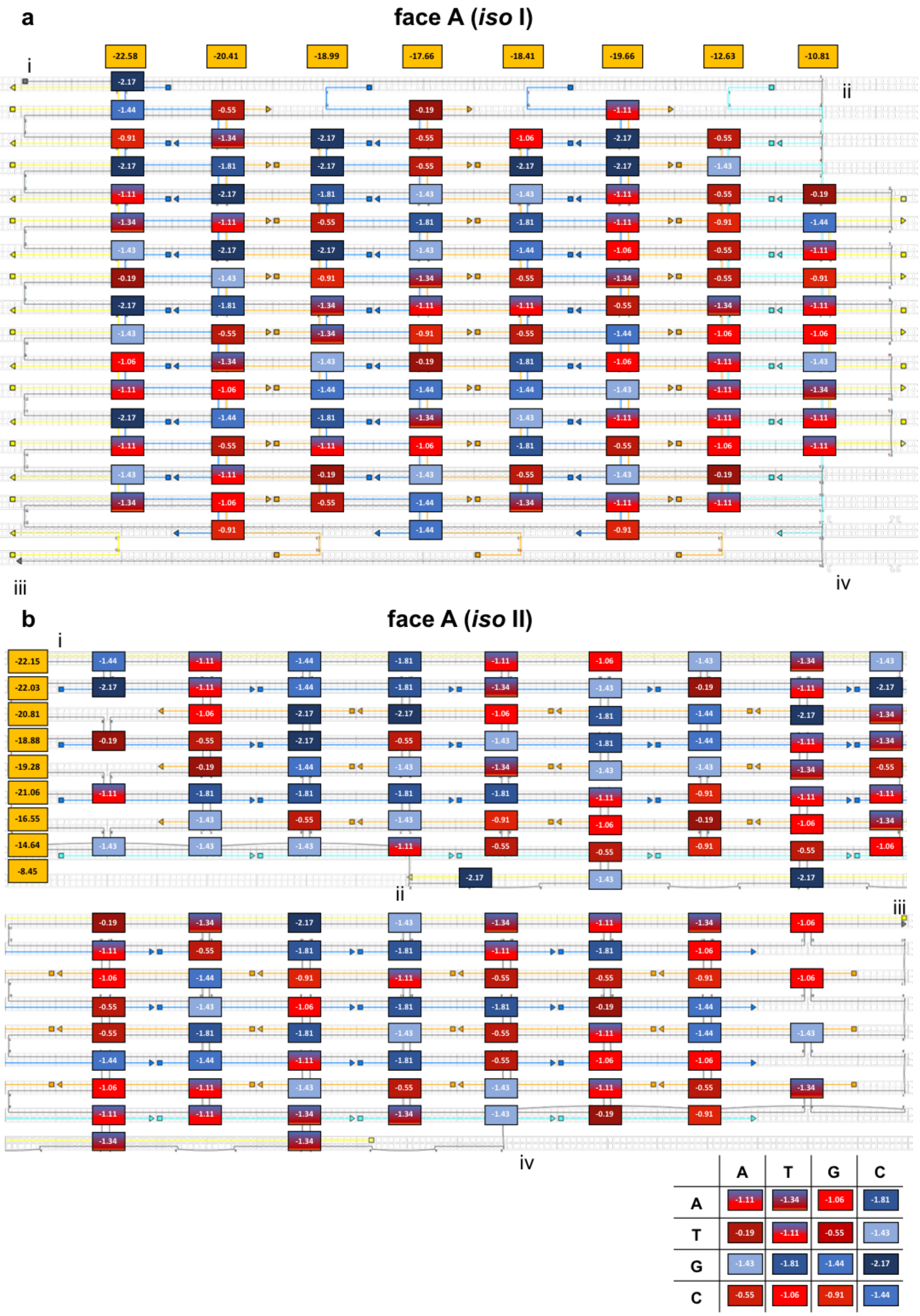

Supplementary Figure 28. Base-stacking map of domain A in the two isomer forms. The base-stacking energy of each crossover has been calculated as the sum of the energetic contributions from the two pairs of stacked bases in each of the two conformations.[4,6] Total values of energy (in kcal/mol) are given for aligned crossovers (yellow boxes).

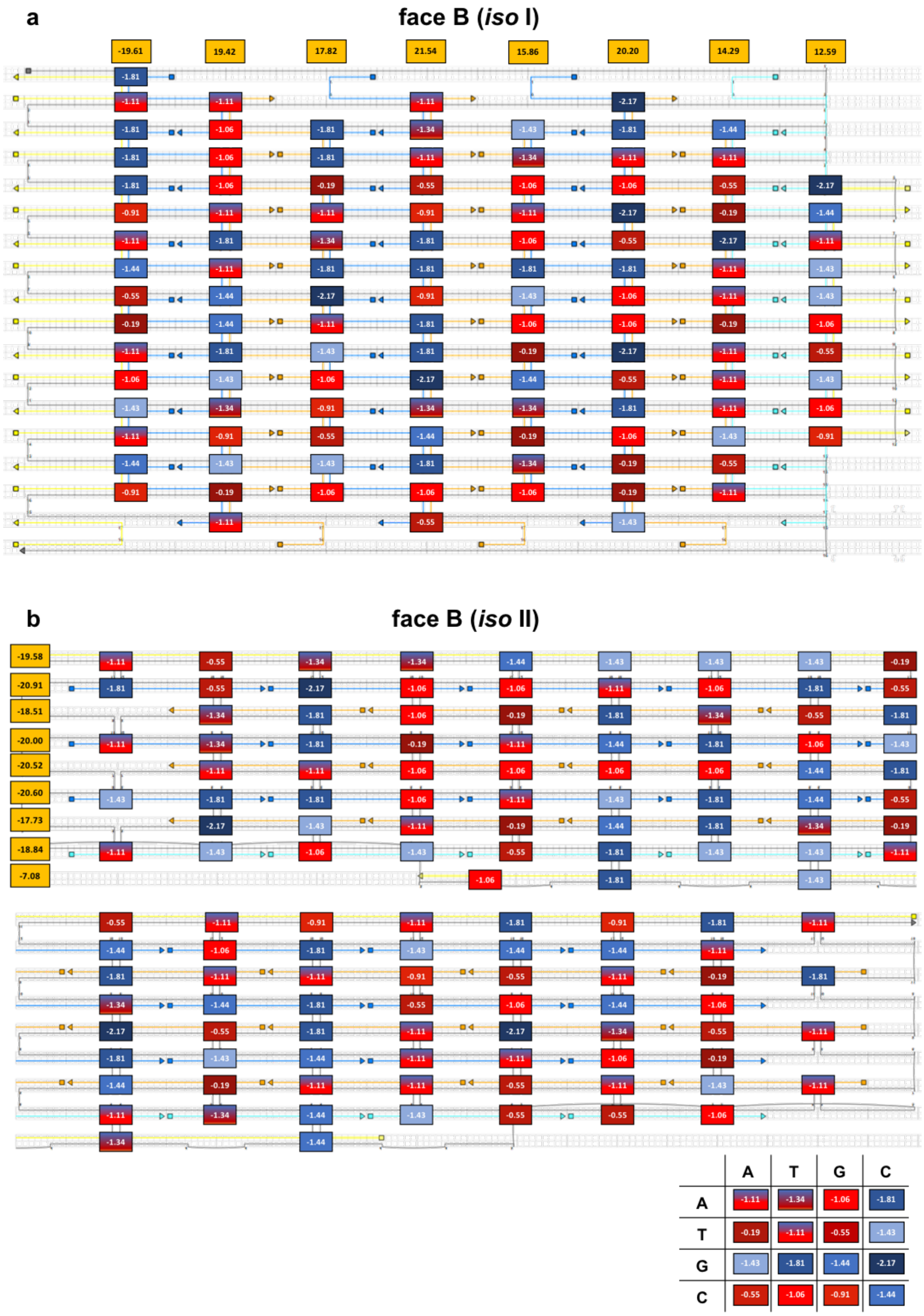

Supplementary Figure 29. Base-stacking map of domain B in the two isomer forms. The base-stacking energy of each crossover has been calculated as the sum of the energetic contributions from the two pairs of stacked bases in each of the two conformations.[4,6] Total values of energy (in kcal/mol) are given for aligned crossovers (yellow boxes).

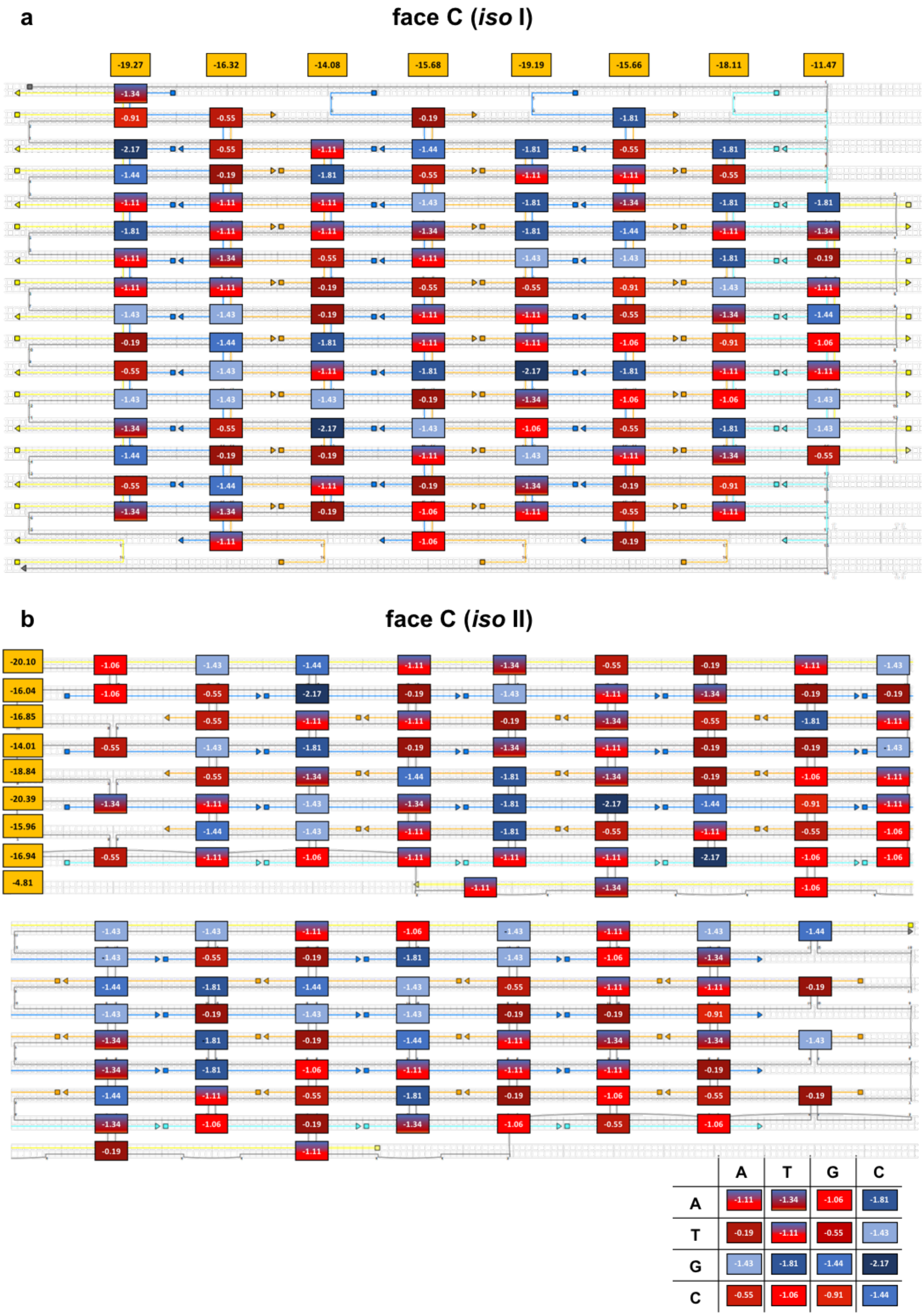

Supplementary Figure 30. Base-stacking map of domain C in the two isomer forms. The base-stacking energy of each crossover has been calculated as the sum of the energetic contributions from the two pairs of stacked bases in each of the two conformations.[4,6] Total values of energy (in kcal/mol) are given for aligned crossovers (yellow boxes).

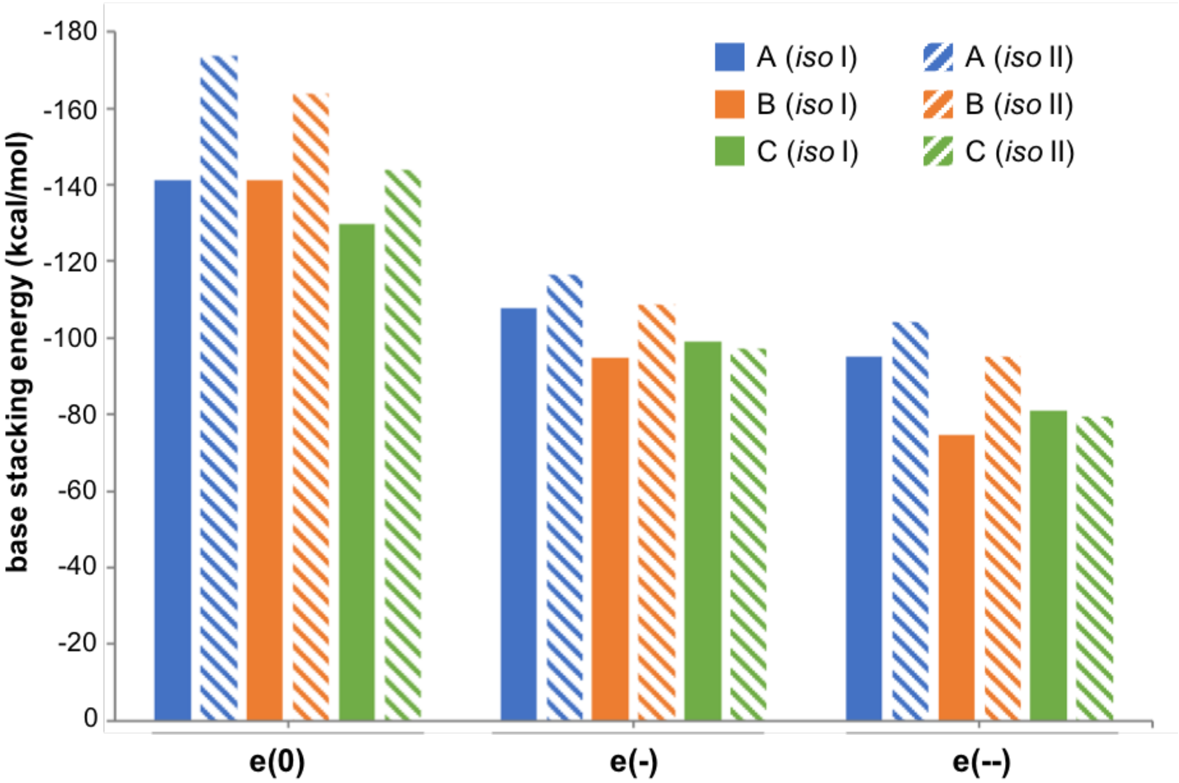

Supplementary Figure 31. Total base-stacking energies of the three domains in the two isomer forms and different edge designs. Base-stacking energies of the three domains were calculated as described in Suppl. Fig. 28-30 considering the contribution from edges type 0 (e(0) construct), their absence (e(-) construct) or the additional lack of the last row of staples on the right-side of the structure (e(--) construct). The following aspects emerge: (i) removal of edges leads to a corresponding decrease of energetic values, as excepted from a reduced number of staples and therefore stacked base pairs; (ii) for each domain, the *iso* II form is more stable than the *iso* I, which is attributed to the formation of additional base stacking interactions between the terminal bases of consecutive edge staples; (iii) domain A is tendentially more stable than the other two domains, which might be due to its particular sequence content; however the difference in stacking energies between the two isomers of the same domain (always in favor of *iso* II) does not correlate with our experimental observations in a consistent manner. Thus, although the sequence content is undoubtedly important in defining the assembly fate, base stacking contributions appear to play a minor (or not yet fully understood) role as compared to the thermal stability of the involved staples. Numerical values are provided in Suppl. Table 8. Source data are provided as a Source Data file.

Note on base-stacking energies:
The base-stacking energy of each crossover has been calculated as the sum of the energetic contributions from the two pairs of stacked bases, using the theoretical values reported in the literature.[4,6] However, recent measurements of isomer ratios on various immobile HJs demonstrate that the relative stability of one form over the other cannot be simply deduced by the difference in base stacking energies between the two pairs of stacked bases. Other factors might be implicated, which probably include the nucleobases in the direct vicinity of the crossover or even the entire sequence of the HJ, making the full energy mapping of an origami structure not a trivial task.

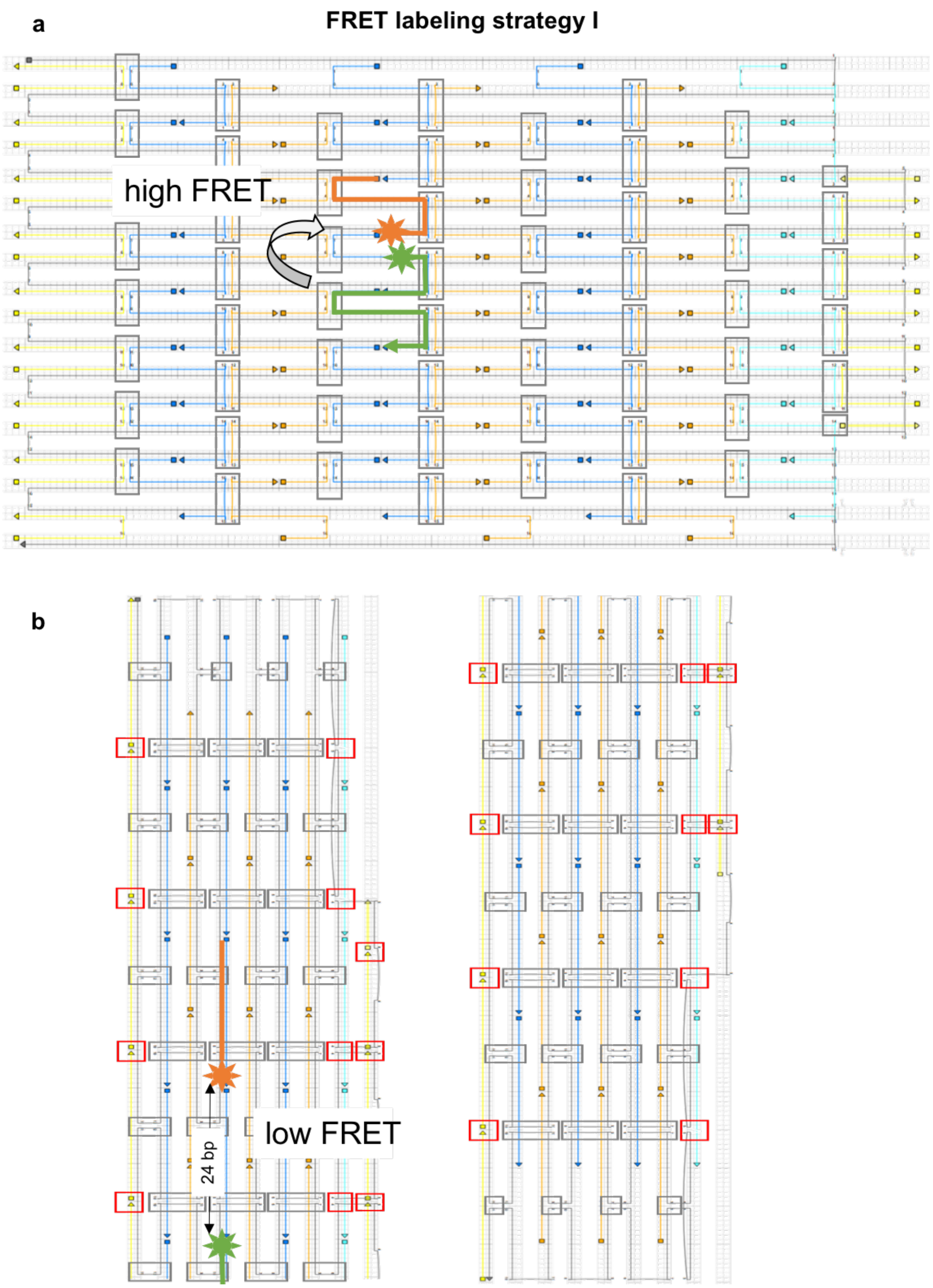

Supplementary Figure 32. FRET labelling strategy I to monitor the formation of the *iso* I conformer only. Two staple strands belonging to the core of domain A were labelled at their termini with a FAM and TAMRA fluorophore, such to give a high FRET signal when assembled in the *iso* I form. The distance between the fluorophores is largely increased (24 bp = ca. 8.2 nm) when the strands are assembled within the *iso* II conformer, resulting in a lower FRET signal.

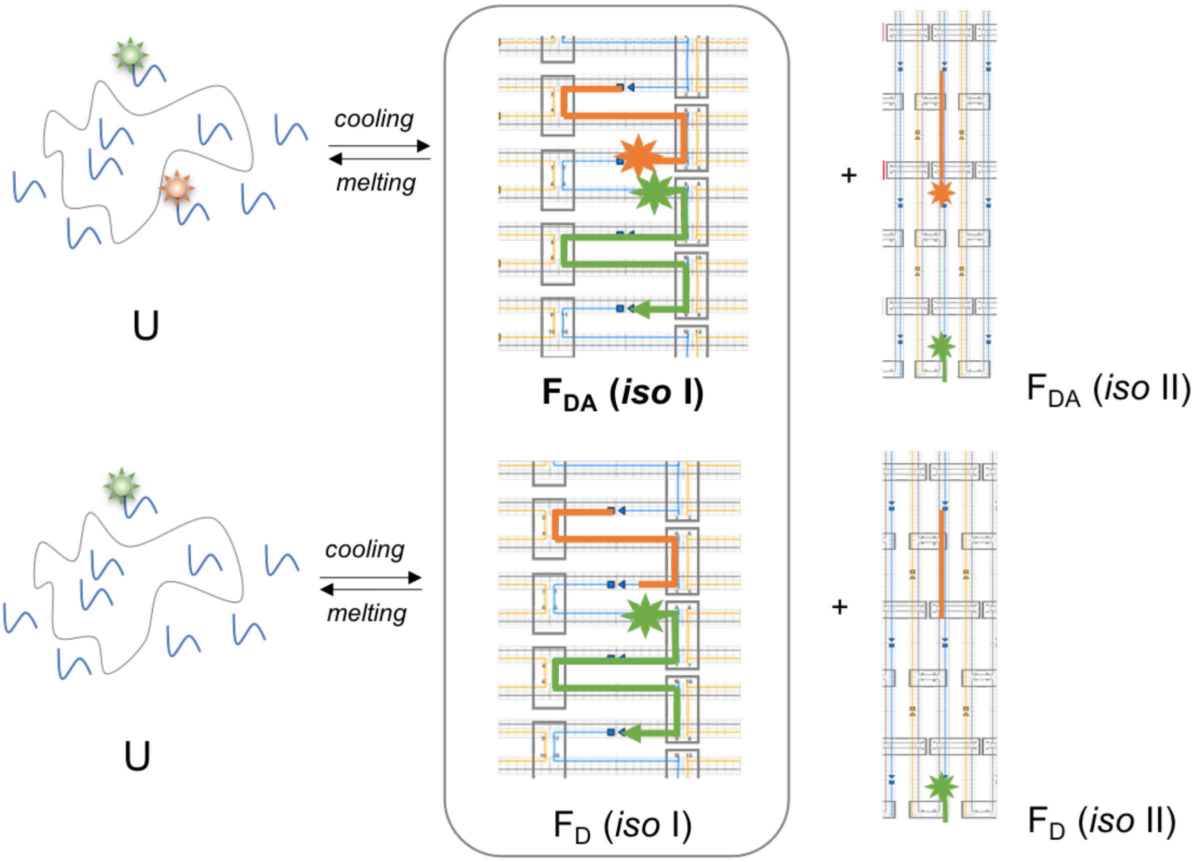

$$E = E_{isoI} + E_{isoII} = \frac{F_{D(isoI)} - F_{DA(isoI)}}{F_{D(isoI)}}$$

Supplementary Figure 33. Schematic representation of the thermal-dependent FRET I strategy. Applying the FRET I strategy to monitor the thermal folding or unfolding of the DNA origami domain and assuming formation of *iso* I and *iso* II isomers in both the donor-acceptor (upper panel) and donor-only (lower panel) sample, only one out of four possible species (F_{DA} (*iso* I)) will give a high FRET signal. The *iso* II isomer, although present, will not be revealed by this FRET assay as the contributions from donor-acceptor and donor-only sample will be approximately equal and cancelled out during the normalization process.

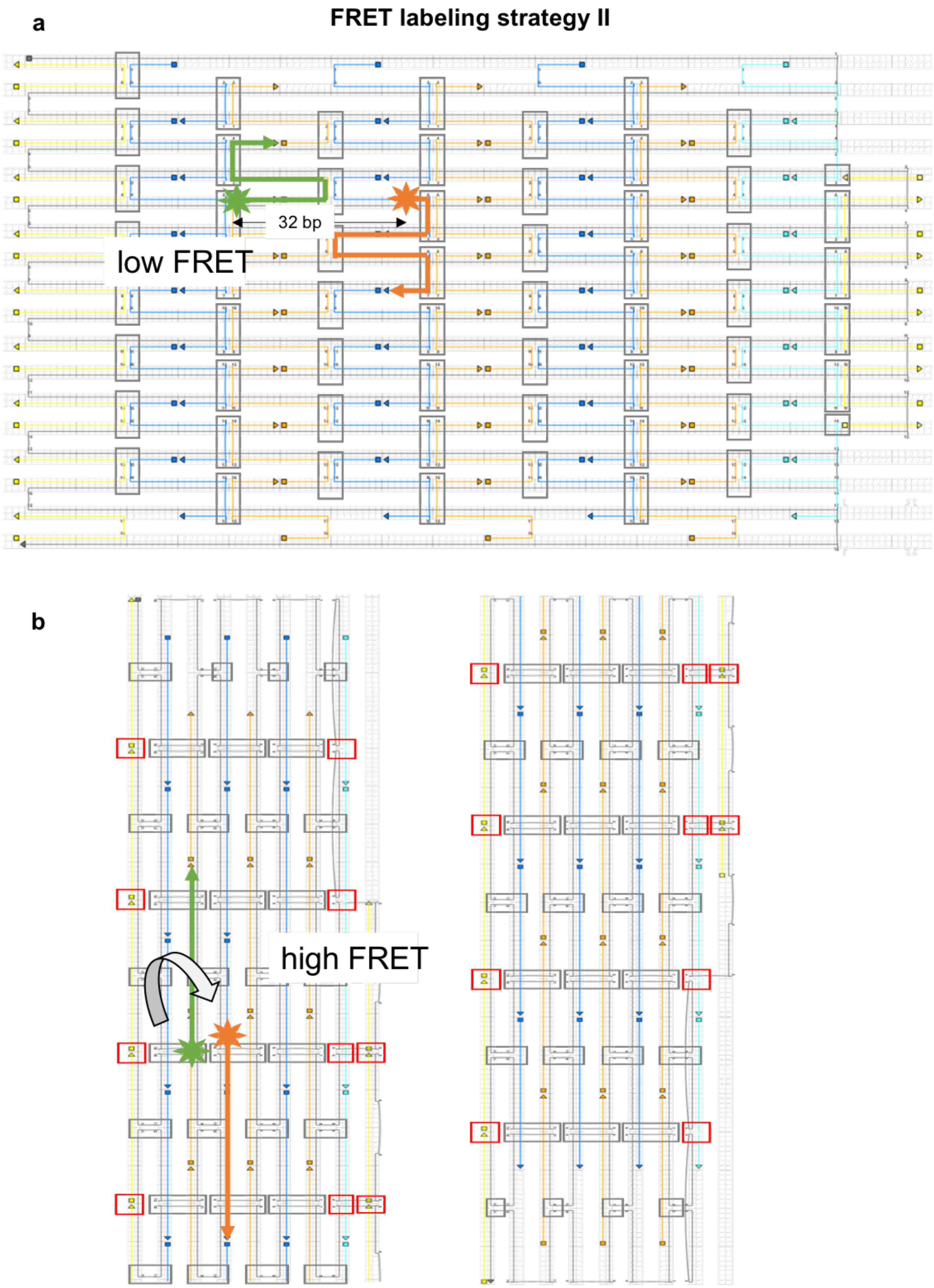

Supplementary Figure 34. FRET labelling strategy II to monitor the formation of the *iso* II conformer only. Two staple strands belonging to the core of domain A were labelled at their termini with a FAM and TAMRA fluorophore, such to give a low FRET signal when assembled in the *iso* I form (32 bp = ca. 11 nm) and a high FRET signal when assembled within the *iso* II conformer.

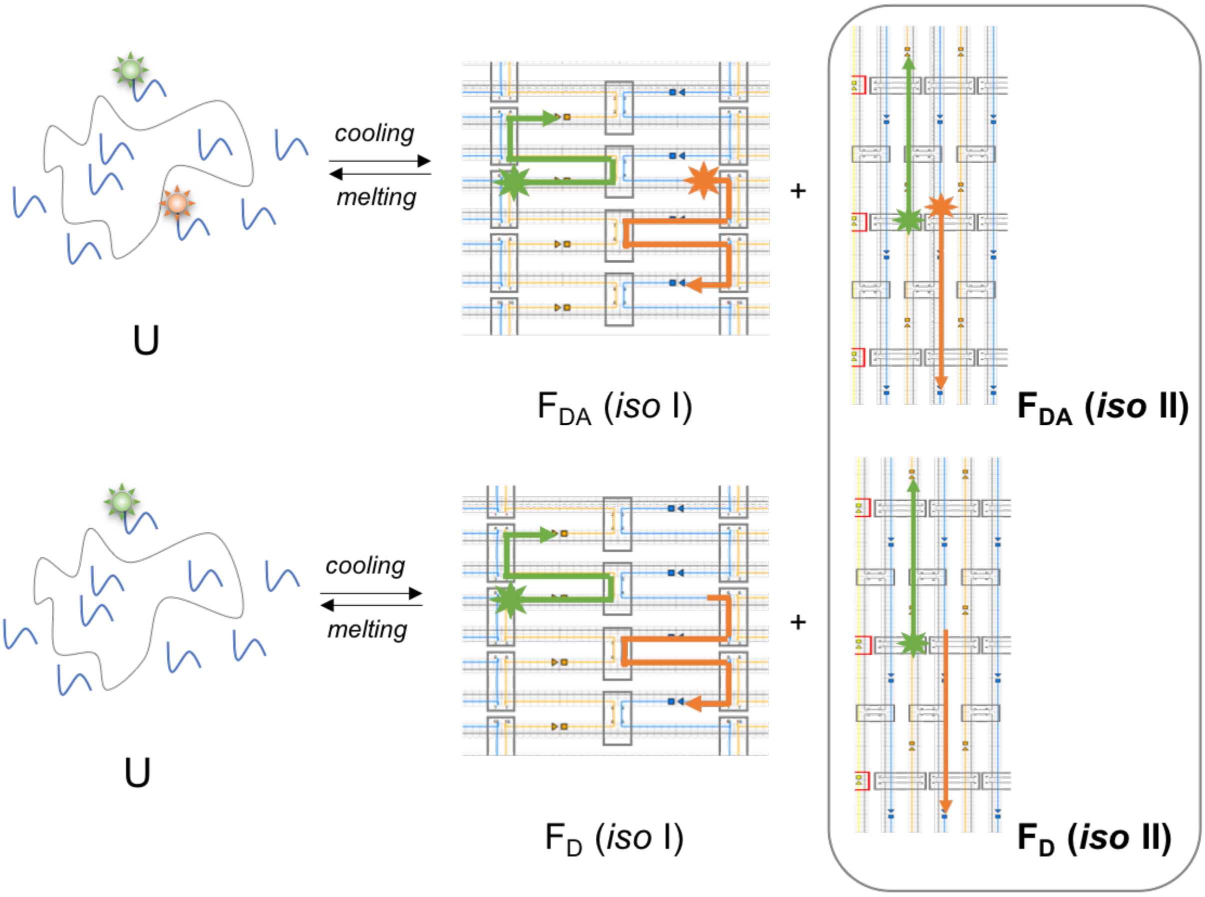

$$E = E_{isoI} + E_{isoII} = \frac{F_{D(isoII)} - F_{DA(isoII)}}{F_{D(isoII)}}$$

Supplementary Figure 35. Schematic representation of the thermal-dependent FRET II strategy. Applying the FRET II strategy to monitor the thermal folding or unfolding of the DNA origami domain and assuming formation of *iso* I and *iso* II isomers in both the donor-acceptor (upper panel) and donor-only (lower panel) sample, only one out of four possible species (F_{DA} (*iso* II)) will give a high FRET signal. The *iso* I isomer, although present, will not be revealed by this FRET assay as the contributions from donor-acceptor and donor-only sample will be approximately equal and cancelled out during the normalization process.

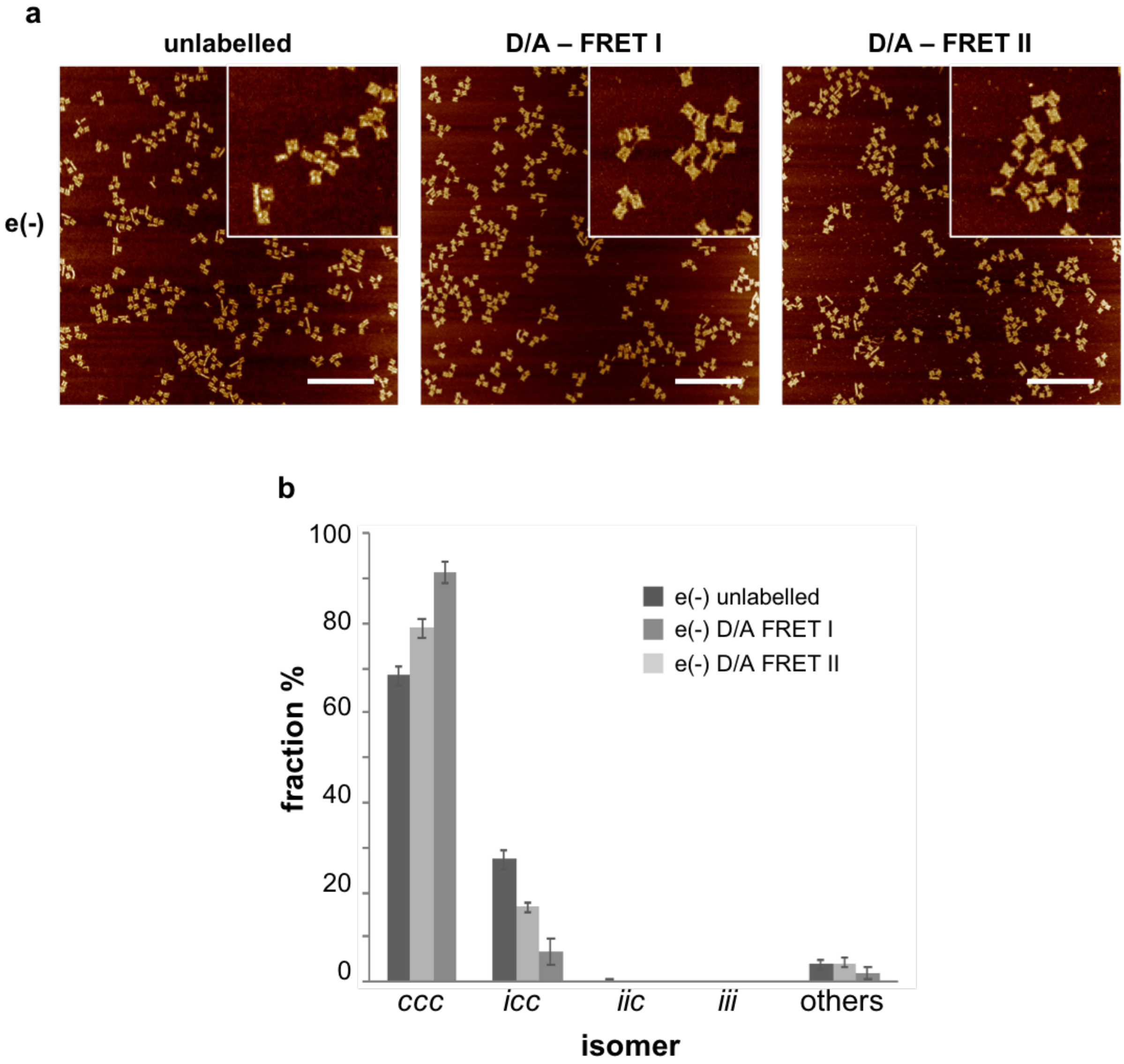

Supplementary Figure 36. Effect of fluorescent labelling on the degree of isomerization. The full DNA origami structure has been assembled in absence of edges using either unlabelled (e(-) unlabelled) or labelled staple strands, bearing the donor/acceptor fluorophores in the FRET I or FRET II configuration (e(-) D/A FRET I and e(-) D/A FRET II, respectively). Representative AFM images (**a**) and statistical analysis of the end products (**b**) show that fluorescent labelling of domain A slightly moves the equilibrium towards the *iso* I conformation. Of particular note is that the labelling strategy adopted involved a change in the length of two or three staples of the core. Interestingly, this modification does not change the sequence content of the domain, but the connectivity between its nucleobases, probably altering the balance of forces at the beginning of the folding process. This confirms that, for small DNA origami domains in absence of topological strain at the edges, both the sequence and connectivity of the bases can affect the assembly fate. Numerical values of the statistical analysis are given in Suppl. Table 9. Error bars were obtained from three independent images of the same sample, each showing several hundreds of individual structures. Source data are provided as a Source Data file.

Note: Analysis of the end products indicates that the presence of the labels slightly alters the *iso* I/*iso* II ratio, such that samples labelled with FRET I strands overestimate the *amount* of *iso* I and samples labelled with FRET II, underestimate the *amount* of *iso* II. However, our thermal FRET experiments are not intended to estimate the relative amount of the two isomers, rather their relative folding/unfolding energies and any possible impact from concentration-dependent effects will be cancelled out by normalization of the donor-acceptor fluorescence signal to the donor-only signal.

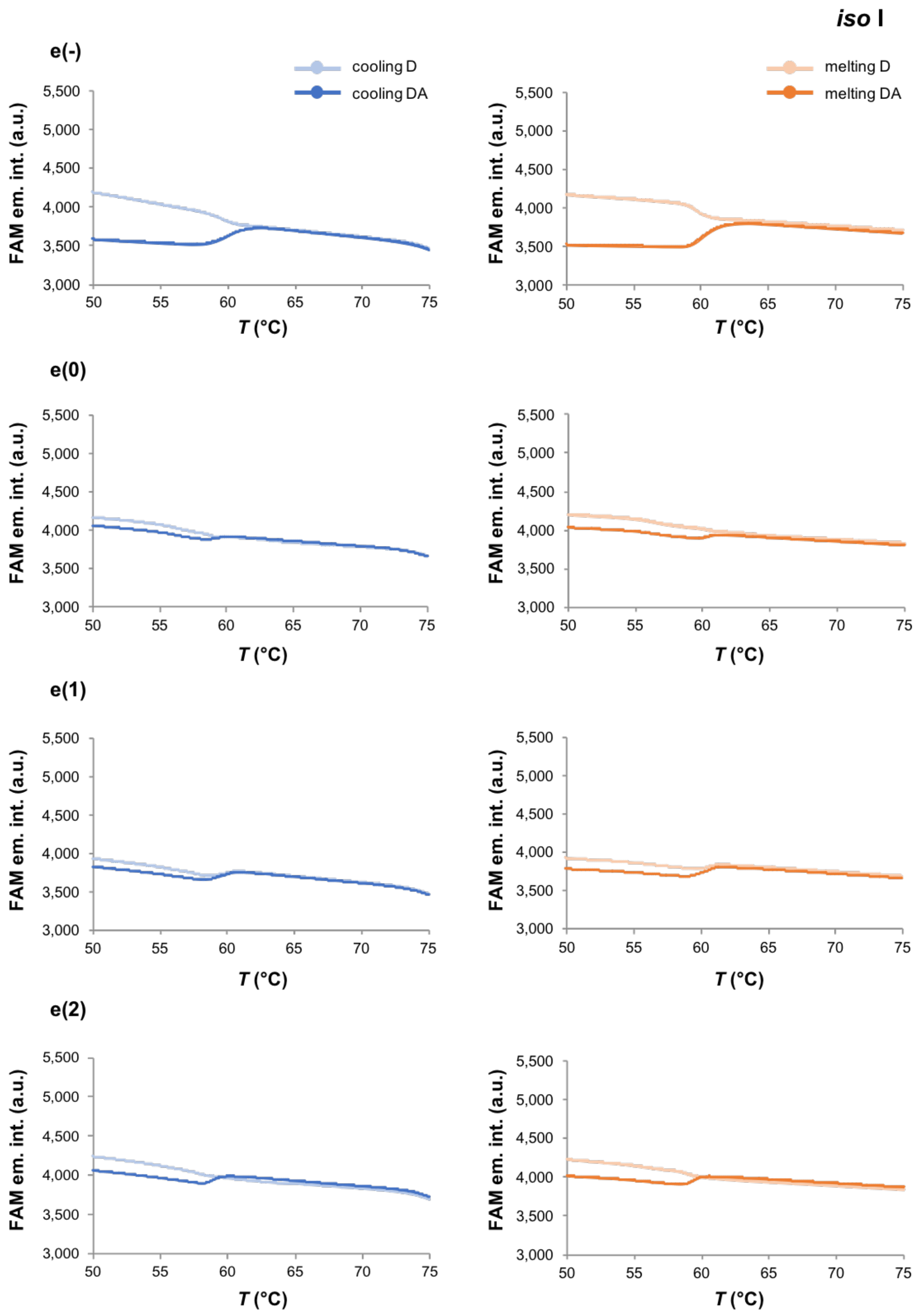

Supplementary Figure 37. Raw thermal profiles of domain A labelled with FRET I reporters. A mixture of 50 nM scaffold and 50X staple strands in 1X TEMg buffer was prepared for each FRET sample. Besides the sequences for assembly of domain B and C, the mixture contained the donor/acceptor (DA) FRET reporters for domain A in the *iso* I form and either no edges (e(-) design) or edges of different types (e(0), e(1) or e(2) constructs). Identical samples were prepared containing the donor-only fluorophore (D). All samples were exposed to cooling (blue curves) or melting (orange curves) in the range between 50°C and 75°C with a rate of +/- 0.1 °C/min to ensure enough equilibration time at each temperature. Data were averaged from four replicates of two independent experiments.

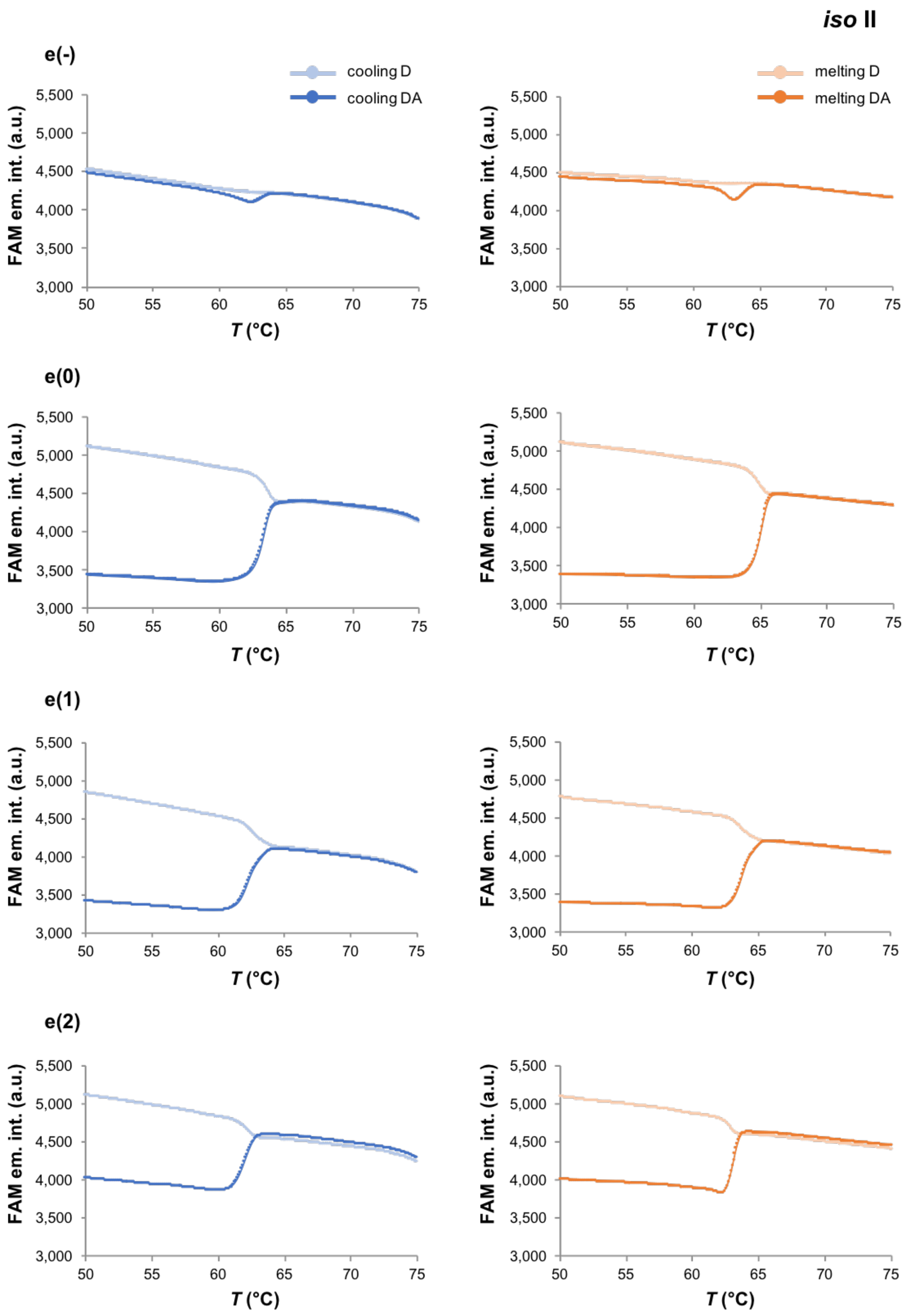

Supplementary Figure 38. Raw thermal profiles of domain A labelled with FRET II reporters. A mixture of 50 nM scaffold and 50X staple strands in 1X TEMg buffer was prepared for each FRET sample. Besides the sequences for assembly of domain B and C, the mixture contained the donor/acceptor (DA) FRET reporters for domain A in the *iso* II form and either no edges (e(-) design) or edges of different types (e(0), e(1) or e(2) constructs). Identical samples were prepared containing the donor-only fluorophore (D). All samples were exposed to cooling (blue curves) or melting (orange curves) in the range between 50°C and 75°C with a rate of +/- 0.1 °C/min to ensure enough equilibration time at each temperature. Data were averaged from four replicates of two independent experiments.

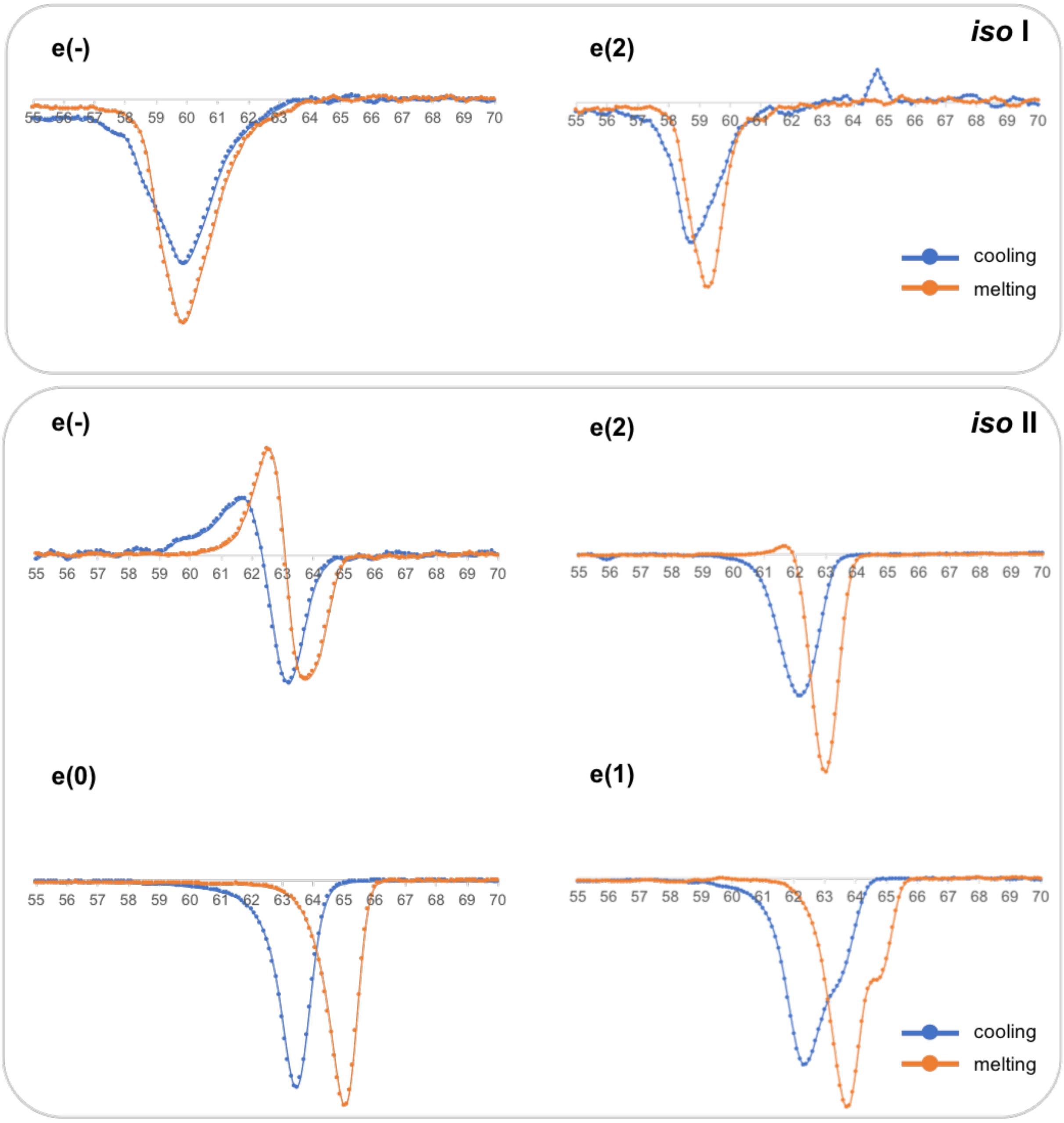

Supplementary Figure 39. First derivatives of the normalized FRET thermal profiles. The thermal profiles shown in Suppl. Fig. 37 and 38 were used to calculate the FRET efficiency (*E*) of the sample at different temperatures and normalized to enable direct comparison of the data obtained for distinct samples. The melting temperature of the structure, intended as the flex point of the *E* vs *T* profile, has been calculated from the minimum of the first derivative of the FRET thermal curve.

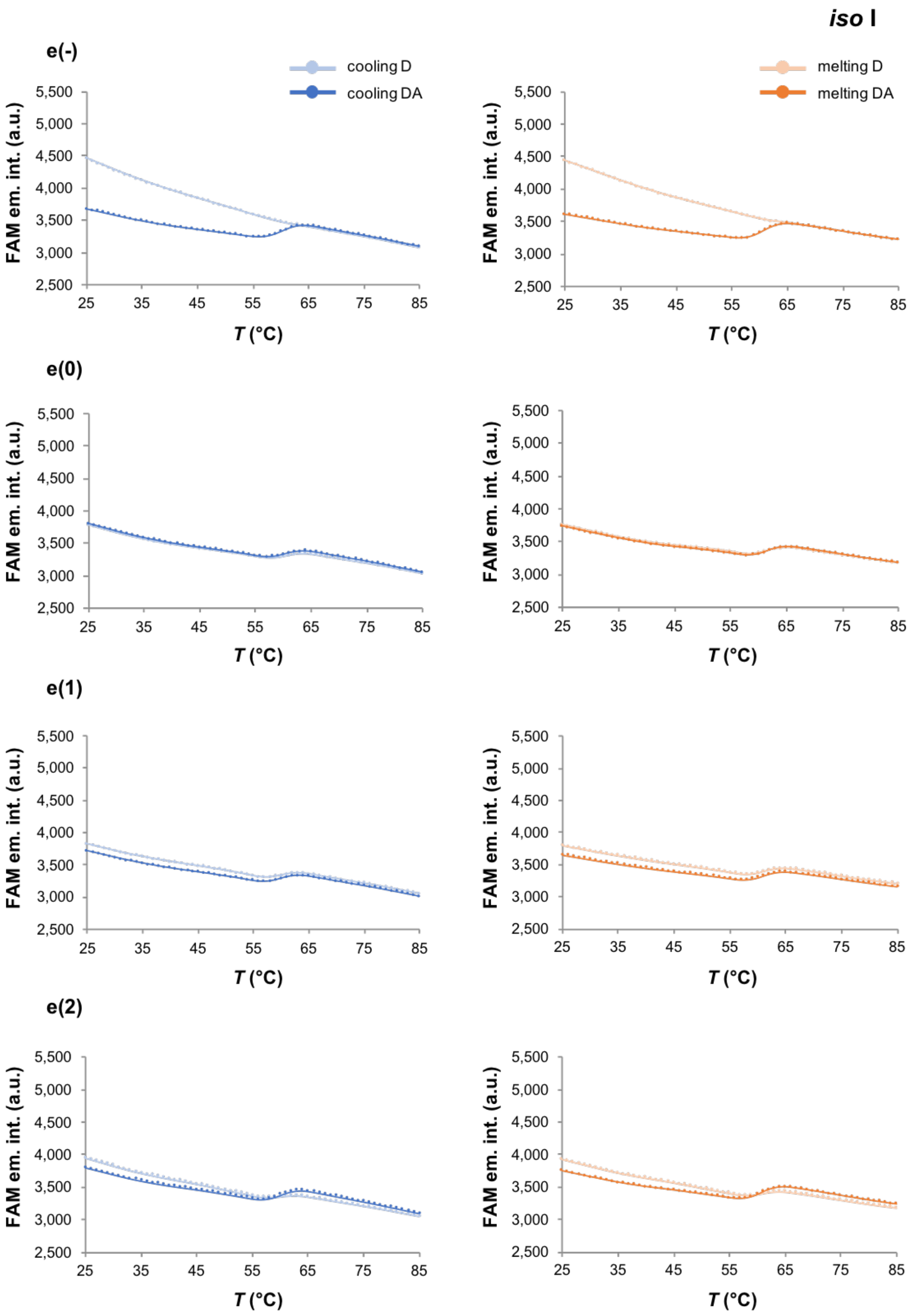

Supplementary Figure 40. Raw thermal profiles of domain A labelled with FRET I reporters. A mixture of 50 nM scaffold and 50X staple strands in 1X TEMg buffer was prepared for each FRET sample. Besides the sequences for assembly of domain B and C, the mixture contained the donor/acceptor (DA) FRET reporters for domain A in the *iso* II form and either no edges (e(-) design) or edges of different types (e(0), e(1) or e(2) constructs). Identical samples were prepared containing the donor-only fluorophore (D). All samples were exposed to cooling (blue curves) or melting (orange curves) in the range between 25°C and 85°C with a rate of +/- 1 °C/min. Data were averaged from four replicates of two independent experiments.

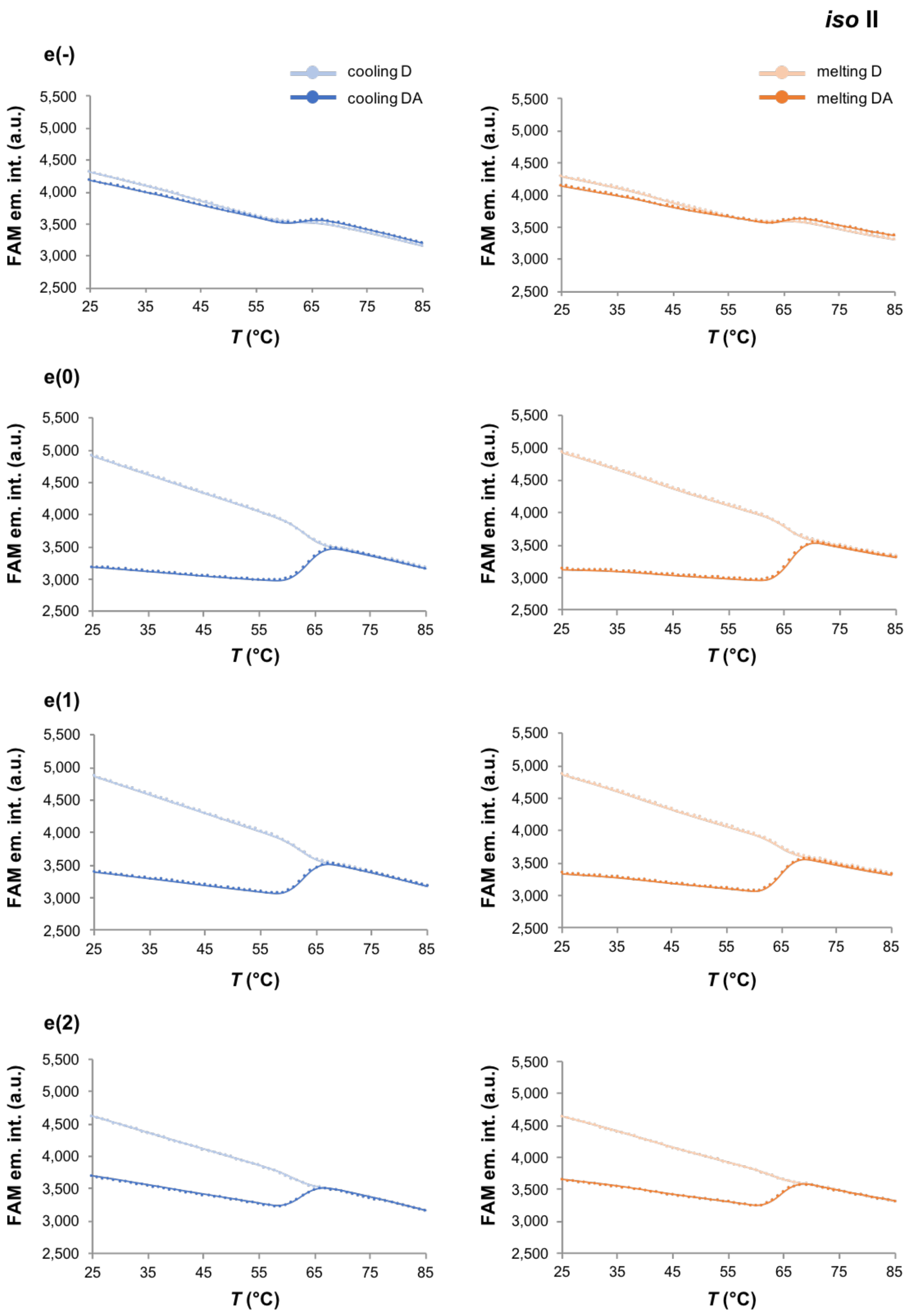

Supplementary Figure 41. Raw thermal profiles of domain A labelled with FRET II reporters. A mixture of 50 nM scaffold and 50X staple strands in 1X TEMg buffer was prepared for each FRET sample. Besides the sequences for assembly of domain B and C, the mixture contained the donor/acceptor (DA) FRET reporters for domain A in the *iso* II form and either no edges (e(-) design) or edges of different types (e(0), e(1) or e(2) constructs). Identical samples were prepared containing the donor-only fluorophore (D). All samples were exposed to cooling (blue curves) or melting (orange curves) in the range between 25°C and 85°C with a rate of +/- 1 °C/min. Data were averaged from four replicates of two independent experiments.

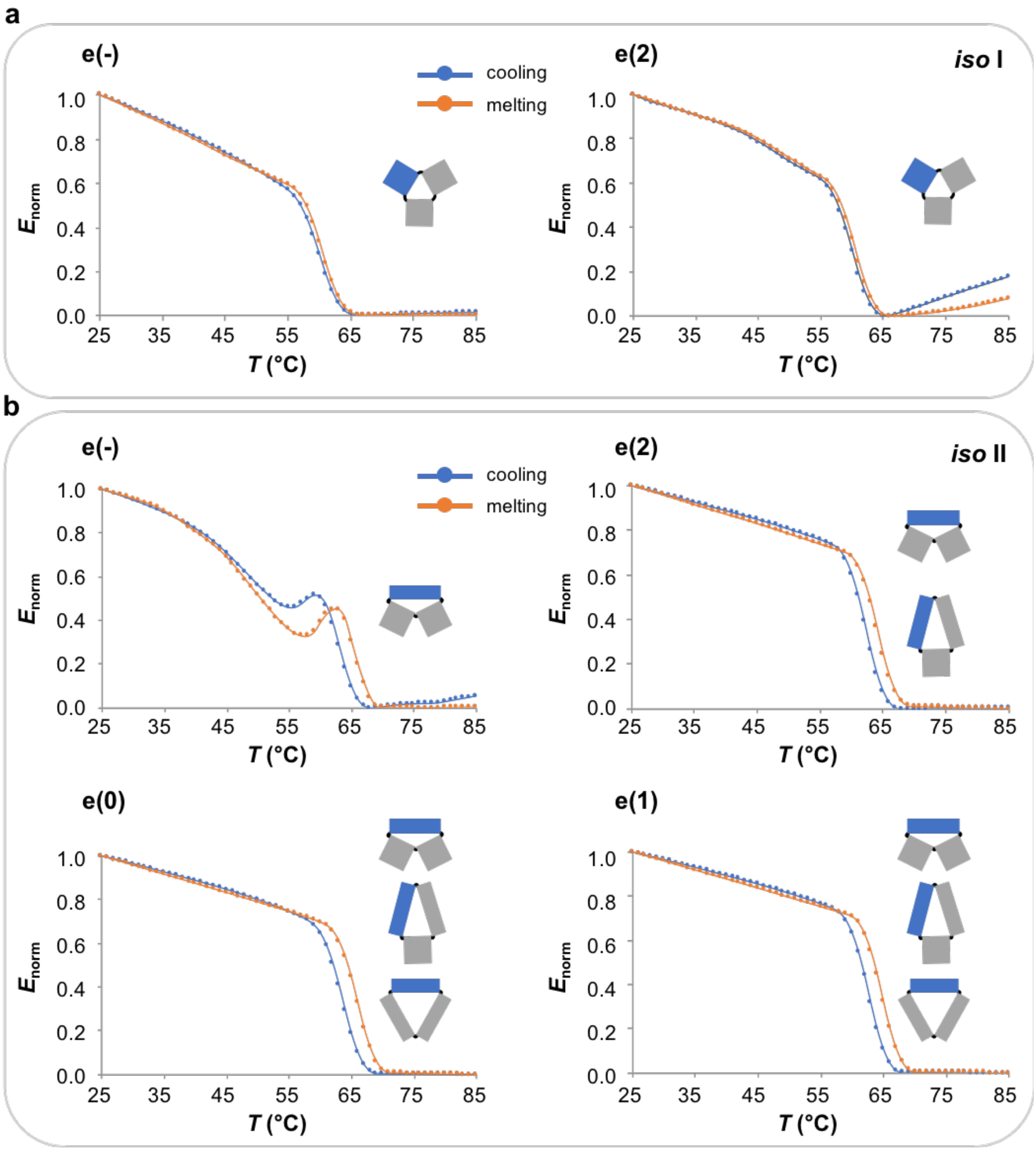

Supplementary Figure 42. Normalized FRET thermal profiles of domain A in the two labelling strategies. The raw data shown in Suppl. Fig. 40 and 41 were used to calculate the FRET efficiency of the sample at each visited temperature in both the *iso* I (**a**) and *iso* II (**b**) conformation and in all four designs analysed. The thermal profiles and melting temperatures obtained were similar to those observed for the same samples exposed to a slower cooling/melting rate (+/- 0.1 °C/min; Fig. 4 of the main manuscript), suggesting that whereas formation of *iso* I is under thermodynamic control, formation of *iso* II (showing thermal hysteresis) is not an equilibrium process. Source data are provided as a Source Data file.

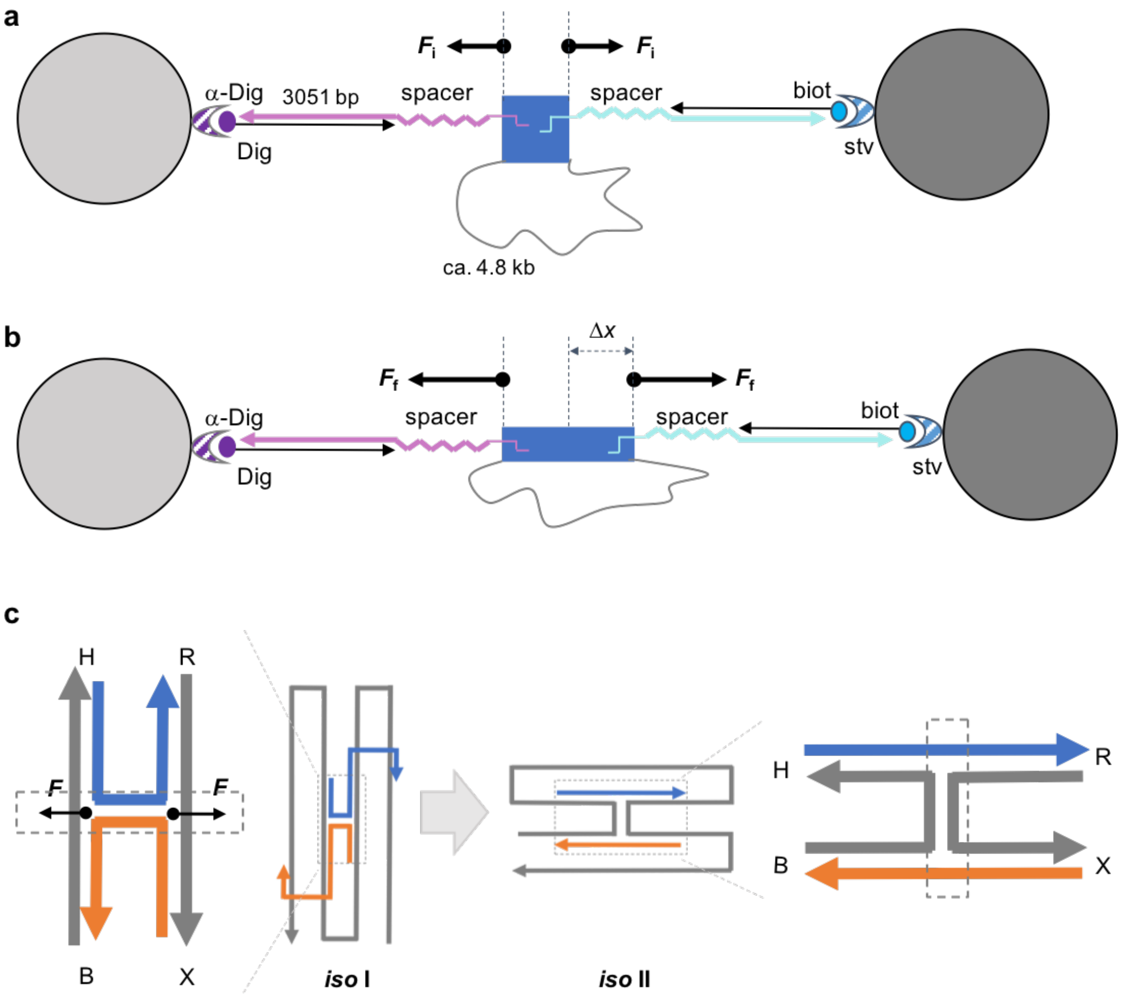

d

1st primer pair:

(FW) 5‘-(origami sequence1) – PEG spacer – Plasmide sequence
GCCAGCTGCGAAAATCCTGTTTGAGTTGTTCC[PEG9]gagatctcgatcctctacgccggacg

(RV) 5‘- digoxigenin plasmide sequence
DIG-aaaggcggacaggtatccggtaagc

2nd primer pair:

(FW) 5‘-(origami sequence2) – PEG spacer – Plasmide sequence
AGCGAACCTTATAGTCAGAAGCAAGAGAGGCT[PEG9]gagatctcgatcctctacgccggacg

(RV) 5‘-biotin plasmide sequence
Biotin-aaaggcggacaggtatccggtaagc

Supplementary Figure 43. Single-molecule optical tweezers measurements on domain A. The domain A in the e(-) design (see details in Suppl. Fig. 44) was trapped between two microspheres in a dual optical tweezer (C-Trap, Lumicks) using digoxigenin/anti-digoxigenin and biotin/streptavidin interactions (**a**). Constant-trap-distance measurements were performed in order to monitor the transition of the single domain from the *iso* I (a) to the *iso* II isomer (**b**) according to a model of mechanical reconfiguration of every single HJs (**c**). The sequence linking each side of the DNA origami domain to the corresponding bead is constituted by a central PEG spacer flanked by two regions partially complementary to the M13 (from one side) and to a ca. 3 kb long tether (from the other side) (**d,** in colour). The primers used for generation of the tethers were obtained from PCR amplification of a pET-28a plasmid (5369 bp) (**d**, in black).

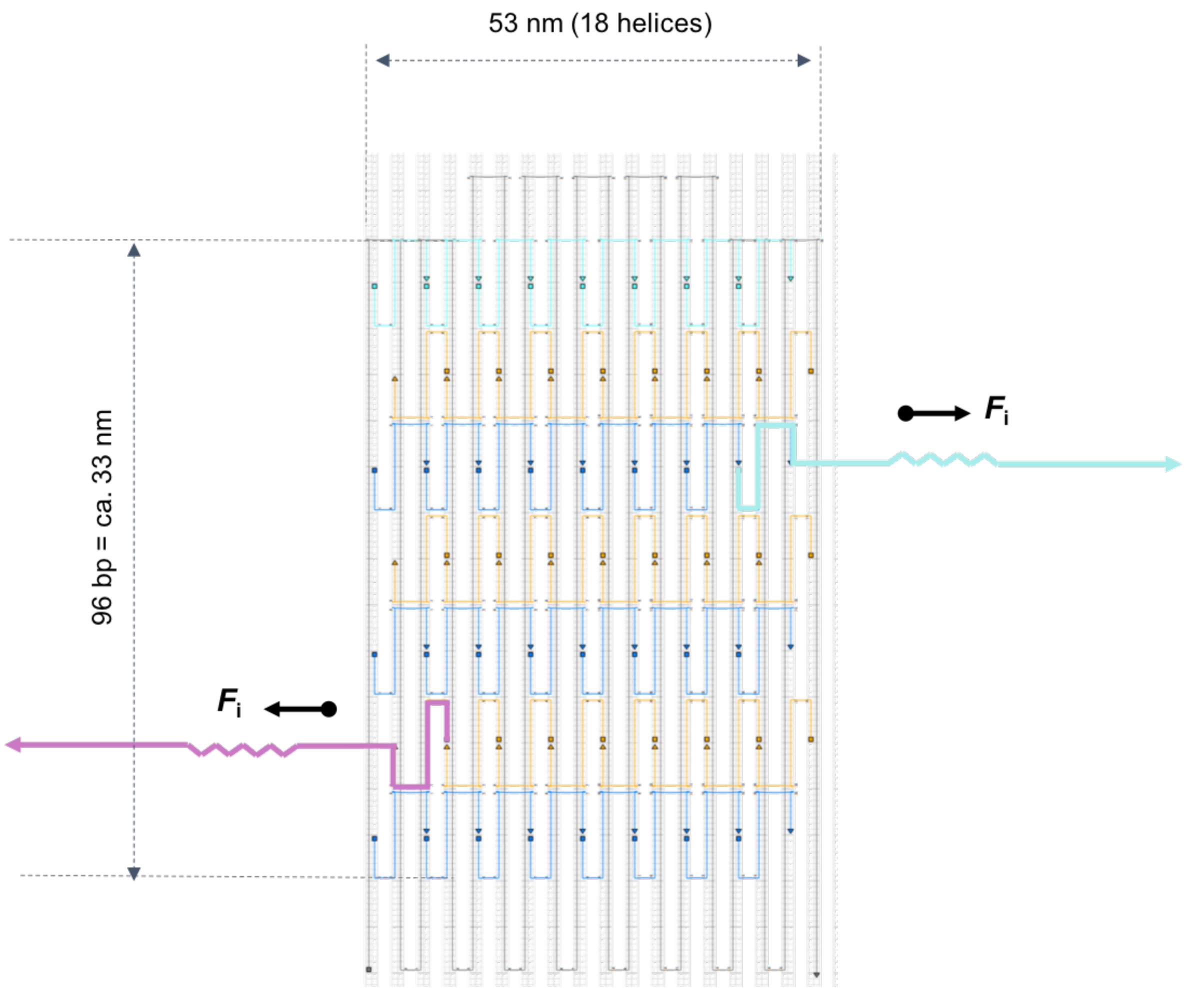

Supplementary Figure 44. Schematic representation of the domain A used for single-molecule optical tweezers measurements. The DNA origami domain has been modified with two linker sequences of equal length pointing towards opposite directions and anchored to the structure perpendicularly to the helical axes (magenta and cyan strands). At low force regimes, the structural reconfiguration from one isomer to the other could be monitored.

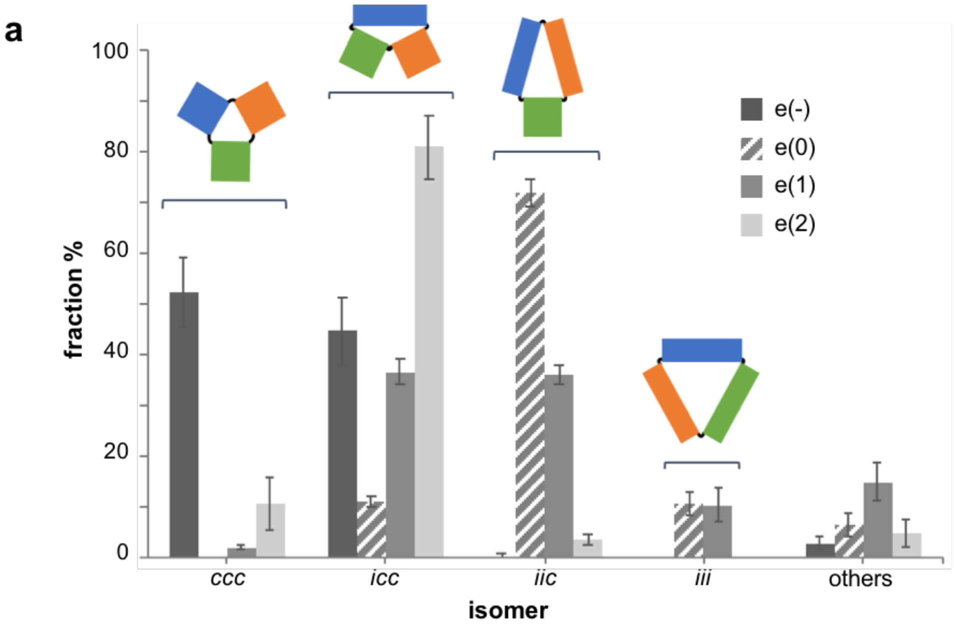

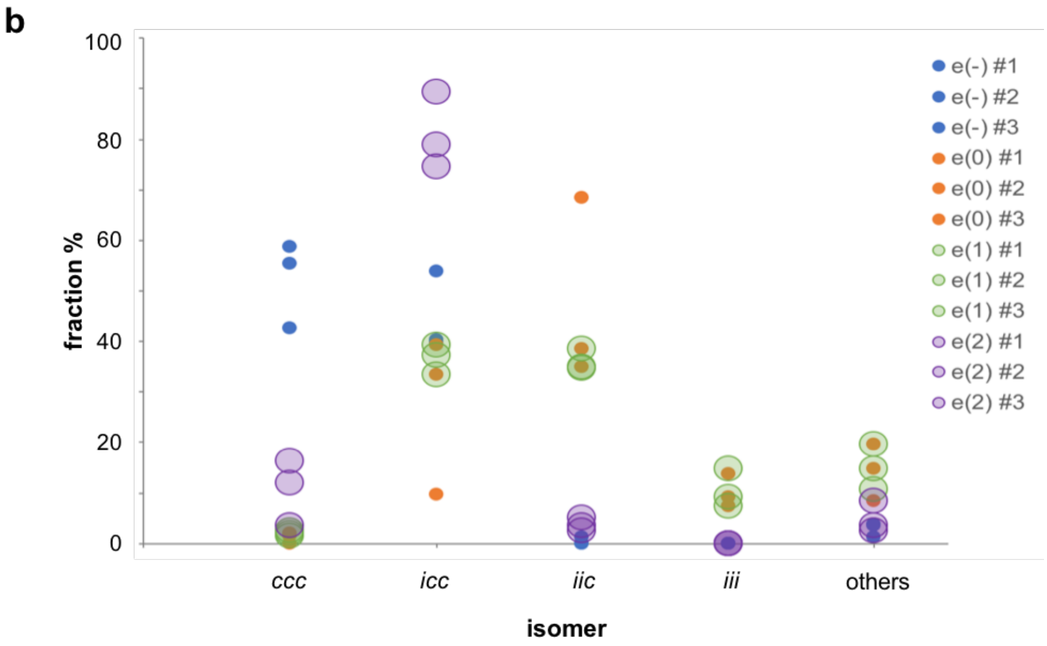

Supplementary Figure 45. Comparison between a bar-chart and a dot-plot representation of single-particle AFM analysis. The full DNA origami structure, either in absence (design e(-)) or presence of edges type 0, 1 or 2 (designs e(0), e(1) or e(2)) was assembled in 2 nM scaffold at -1°C/min. The results are displayed as a bar diagram (**a**) or as a dot-plot (**b**). The fraction of isomeric species present in each construct was determined by manual counting over a few hundreds of structures. Three independent measurements were performed for each construct, counting the individual structures adsorbed onto different regions of the mica support or obtained from different sample preparations. Each of these measurements is represented by a circle in the dot-plot. Standard deviations are indicated by error bars in (a) and by the relative distance along the vertical axis in (b). The two representations therefore provide the same information on the reproducibility of the data obtained for each conformation and their distribution in different design and experimental conditions.

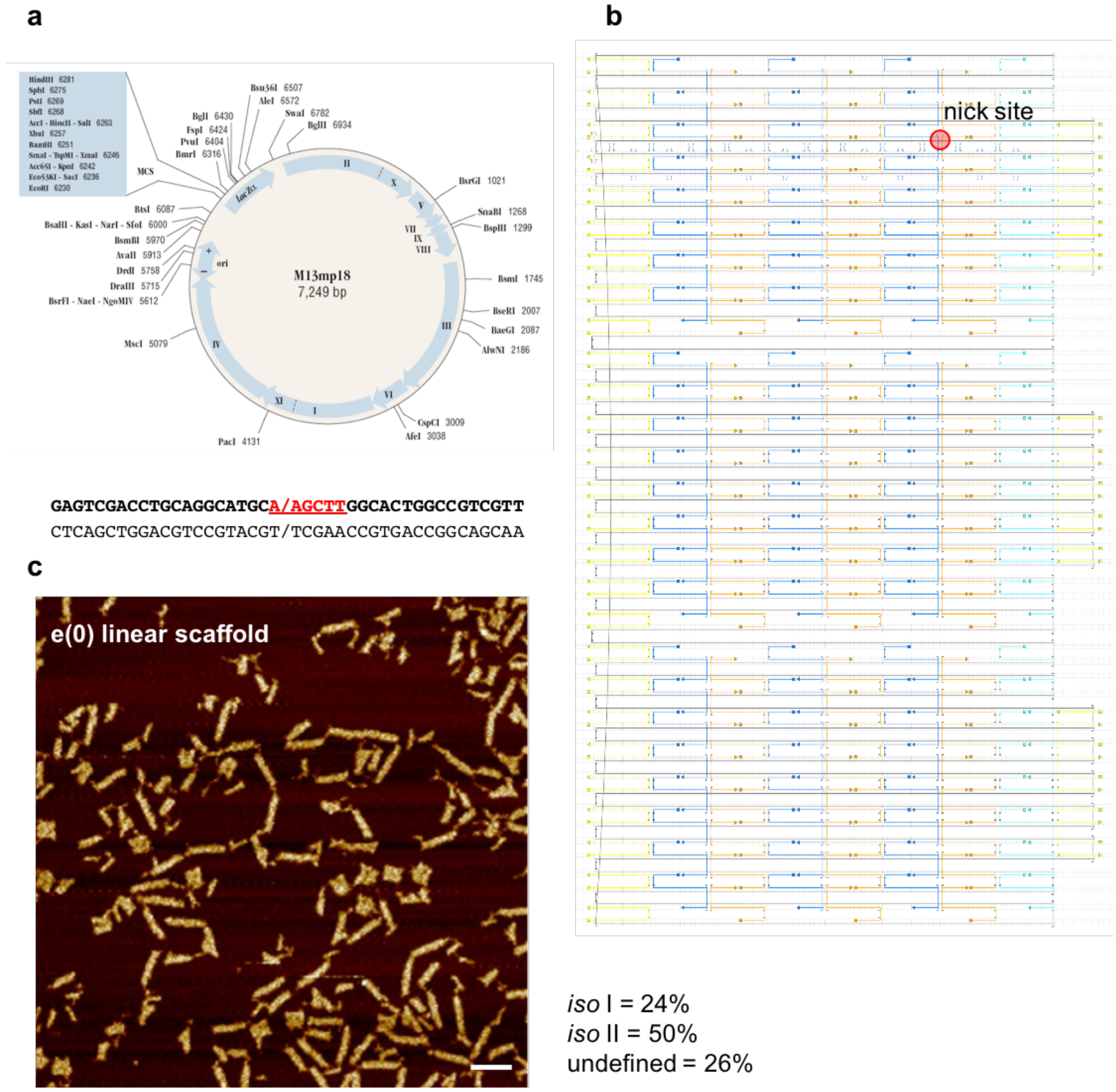

Supplementary Figure 46. Effect of scaffold linearization on the isomerization of domain A. Map of the enzymatic restriction sites and corresponding enzymes for the M13mp18 sequence (**a**). Hind III cleaves the scaffold at one defined position (indicated in red and bold), enabling to get a linear sequence starting from a circular one. Upon DNA origami assembly, the nick site of the scaffold is located within the core of domain A (**b**). The end products of the assembly using a linear scaffold and edges type 0 show about 24% of *iso* I forms, 50% of *iso* II and 26% of undefined structures (**c**). Scale bar is 100 nm. The observed fraction of isomerization is therefore only slightly reduced when compared to the use of a circular scaffold (62% of *iso* II forms), suggesting that the topological constraint induced by circularization of the scaffold is not the main cause for isomerization of the structure. In other words, the major input of mechanical stress experienced by the domain does not originate from the scaffold being circular. In view of our studies, we deduce that this is due to introduction of the nick into a region that is distant from the nucleation strands at the edges and has a lower thermal stability, thus affecting the fate of the assembly only in minimal part. The situation might be different when the nick is introduced at one of the edge strands, particularly if involved in the initiation of the assembly process. In this case, we presume that the conditions are met to release the mechanical stress applied without the need for isomerization of the entire domain.

Supplementary Table 1. Statistical AFM analysis of the full DNA origami structure in absence or presence of left- or right-side edges. The full structure was assembled in one-pot reaction in absence of any edges, (e(-) construct), resulting in the appearance of shapes where only one of the three domains is isomerized (*icc*), for a total of ca. 15% of all domains in the *iso* II form. Identical reaction mixtures were prepared and assembled in one-pot together with the staples that target either the left (L) or the right (R) side of each domain. Three sets of edges were used, either type 0, 1 or 2. The ends products of the thermal assembly were visualized at the AFM and counted manually. Standard deviations were obtained from three different images of the same sample for *n* distinct structures. Assembly conditions were 2 nM scaffold, 50X staples in 1X TEMg at -1°C/min.

			left-side edges			***right-side edges***		
	isomers	**e(-)**	**e(0)**	**e(1)**	**e(2)**	**e(0)**	**e(1)**	**e(2)**
STANDARD ASSEMBLY 2 nM, -1°C/min	*tot i-form*	15.1	61.8	49.5	26.8	18.8	16.3	16.6
	tot c-form	84.9	38.2	50.5	73.2	81.2	83.7	83.4
	ccc	52.2 ± 7.0	-	1.5 ± 0.2	20.0 ± 1.4	43.1 ± 3.6	49.6 ± 6.2	47.3 ± 2.2
	icc	44.6 ± 6.5	14.0 ± 0.8	37.8 ± 0.5	74.7 ± 1.4	51.3 ± 2.8	48.9 ± 5.9	49.9 ± 2.2
	iic	0.4 ± 0.6	68.2 ± 2.5	40.6 ± 2.3	2.8 ± 0.6	2.5 ± 1.3	-	-
	iii	-	11.7 ± 1.8	9.8 ± 1.1	-	-	-	-
	others	2.8 ± 1.2	6.1 ± 0.2	10.3 ± 2.0	2.5 ± 1.0	3.0 ± 0.5	1.5 ± 0.4	2.8 ± 0.9
	n	*604*	*555*	*526*	*402*	*465*	*527*	*482*

Supplementary Table 2. Statistical AFM analysis of the full DNA origami structure in absence or presence of both the left- and right-side edges. The full structure was assembled in one-pot reaction either in absence of any edges, (e(-) construct) or in presence of the right- and left-side edges of type 0, 1 or 2 (e(0), e(1) and e(2) constructs, respectively). The ends products of the thermal assembly were visualized at the AFM and counted manually. Standard deviations were obtained from three different images of the same sample for *n* distinct structures. Assembly conditions were 2 nM scaffold at -1°C/min (standard assembly), 2 nM at -1°C/20 min (long annealing time) and 50 nM at -1°C/min (high concentration). In all cases, the assembly buffer was 1X TEMg with 50X staple strands.

		edges			
	isomers	**e(-)**	**e(0)**	**e(1)**	**e(2)**
STANDARD ASSEMBLY 2 nM, -1°C/min	*tot i-form*	15.1	62.2	46.6	29.4
	ccc	52.2 ± 7.0	-	2.0 ± 0.4	10.6 ± 5.3
	icc	44.6 ± 6.5	11.0 ± 1.0	36.6 ± 2.4	80.9 ± 6.2
	iic	0.4 ± 0.6	71.7 ± 2.6	36.0 ± 1.7	3.7 ± 1.1
	iii	-	10.7 ± 2.2	10.4 ± 3.3	-
	others	2.8 ± 1.2	6.6 ± 2.2	15.0 ± 3.6	4.9 ± 2.6
	n	*604*	*470*	*402*	*195*
LONG ANNEALING TIME 2 nM, -1°C/20min tot 20h	*tot i-form*	7.9	45.9	28.3	17.2
	ccc	55.5 ± 4.4	-	-	21.3 ± 6.5
	icc	23.6 ± 4.7	-	6.0 ± 3.5	51.7 ± 9.8
	iic	-	54.9 ± 5.9	21.3 ± 7.0	-
	iii	-	9.3 ± 1.2	12.1 ± 7.4	-
	others	20.9 ± 0.4	35.8 ± 6.0	60.6 ± 2.8	27.0 ± 4.2
	n	*124*	*108*	*113*	*59*
HIGH CONCENTRATION 50 nM, -1°C/min	*tot i-form*	9.2	65.9	51.2	23.2
	ccc	68.4 ± 2.2	-	1.2 ± 1.7	36.7 ± 4.6
	icc	27.5 ± 2.1	9.2 ± 6.4	42.0 ± 4.9	57.1 ± 5.6
	iic	0.2 ± 0.3	73.0 ± 6.1	46.6 ± 5.3	6.3 ± 8.8
	iii	-	14.1 ± 1.2	6.2 ± 1.8	-
	others	4.0 ± 1.2	3.6 ± 1.2	4.1 ± 1.1	-
	n	*422*	*142*	*300*	*60*

Supplementary Table 3. Statistical AFM analysis of the assembly products of the full DNA origami structure in absence or presence of left-side e(0) edges of different length. The three domains were assembled in one-pot reaction either in absence of any edges, (e(-) design) or in presence of the right- and left-side edges of type 0. Full-length edge staples (e(0) construct) result in a high degree of isomerization. As predicted by our torque-model, e(0) staples on the left-side of the structure that are half-helical turn shorter on both the 5' and 3' ends (e(short) construct) lead instead to a minimal deviation from the core design. The ends products of the thermal assembly were visualized at the AFM and counted manually. Standard deviations were obtained from three different images of the same sample for *n* distinct structures. Assembly conditions were 2 nM or 5 nM scaffold at -1°C/min (standard assembly), in 1X TEMg with 50X staple strands.

		edges		
	isomers	**e(-)**	**e(short)**	**e(0)**
2 nM, -1°C/min	*tot i-form*	15.1	19.2	62.2
	ccc	52.2 ± 7.0	25.8 ± 1.9	-
	icc	44.6 ± 6.5	54.9 ± 0.7	11.0 ± 1.0
	iic	0.4 ± 0.6	1.3 ± 0.4	71.7 ± 2.6
	iii	-	-	10.7 ± 2.2
	others	2.8 ± 1.2	18.0 ± 2.9	6.6 ± 2.2
	n	*250*	*381*	*470*
5 nM, -1°C/min	*tot i-form*	15.3	19.0	60.7
	ccc	53.3 ± 7.0	28.7 ± 3.9	-
	icc	42.5 ± 6.5	54.8 ± 4.3	9.5 ± 1.0
	iic	1.7 ± 0.6	1.1 ± 0.8	73.7 ± 2.6
	iii	-	-	8.4 ± 2.2
	others	2.5 ± 1.2	15.4 ± 0.7	8.4 ± 2.2
	n	*362*	*468*	*357*

Supplementary Table 4. Statistical AFM analysis of the assembly products of the full DNA origami and its single domains in absence of the last raw of right-side staples. The full DNA origami as well as the three domains were individually assembled in absence of the last row of right-side staples (cyan strands in Suppl. Fig. 16). The omission of these staples favoured formation of the isomerized species, both in the absence (e(--) construct) or presence (e(0-) construct) of the left-side edges of type 0. The total fraction of *iso* II domains was 15.3% and 60.7%, for e(-) and e(0), respectively (Suppl. Table 3). Omitting the last right-sided edges, *iso* II percentage increased to 42.5% and 85.8%, for e(--) and e(0-) constructs, respectively, confirming the role of topological stress in the rate of isomerization. The ends products of the thermal assembly were visualized at the AFM and counted manually. Standard deviations were obtained from three different images of the same sample for *n* distinct structures. Assembly conditions were 5 nM scaffold at -1°C/min in 1X TEMg with 50X staple strands.

		edges					
		**e(--)**			**e(0-)**		
5 nM, -1°C/min	***isomers***	**A + B + C**					
	tot i-form	42.5			85.8		
	ccc	9.5 ± 4.0			-		
	icc	42.7 ± 6.8			0.4 ± 0.5		
	iic	38.8 ± 4.4			41.7 ± 1.7		
	iii	2.4 ± 0.7			57.9 ± 1.3		
	others	6.6 ± 3.5			10.4 ± 2.9		
	n	*211*			*296*		
		**A**	**B**	**C**	**A**	**B**	**C**
	can	23.6 ± 2.4	33.5 ± 2.1	95.4 ± 0.7	-	0.3 ± 0.4	33.4 ± 1.8
	iso	72.3 ± 4.0	60.3 ± 0.6	3.9 ± 0.7	99.8 ± 0.3	96.5 ± 0.5	55.5 ± 4.9
	others	4.0 ± 1.7	6.2 ± 1.5	0.8 ± 0.1	0.2 ± 0.3	3.2 ± 0.6	11.1 ± 3.1
	n	*250*	*254*	*258*	*464*	*319*	*304*

Supplementary Table 5. Statistical AFM analysis of the assembly products of the full DNA origami and its single domains in absence and presence of both the left- and right-side staples. The full structure, as well as each individual domain, was assembled in one-pot reaction either in absence of any edges, (e(-) construct) or in presence of the right- and left-side edges of type 0 (e(0) construct). The ends products of the thermal assembly were visualized at the AFM and counted manually. Standard deviations were obtained from three different images of the same sample for *n* distinct structures. Assembly conditions were 5 nM scaffold at -1°C/min in 1X TEMg with 50X staple strands.

		edges					
		**e(-)**			**e(0)**		
5 nM, -1°C/min	***isomers***	**A + B + C**					
	tot i-form	15.3			60.7		
	ccc	53.3 ± 3.3			-		
	icc	42.5 ± 2.7			9.5 ± 1.8		
	iic	1.7 ± 0.6			73.7 ± 4.1		
	iii	-			8.4 ± 1.4		
	others	2.5 ± 0.4			8.4 ± 1.6		
	n	*362*			*357*		
		**A**	**B**	**C**	**A**	**B**	**C**
	can	55.4 ± 2.5	95.3 ± 4.6	97.3 ± 0.5	-	9.3 ± 2.3	83.9 ± 2.2
	iso	40.9 ± 4.8	0.8 ± 0.1	-	94.0 ± 3.3	84.9 ± 2.5	9.1 ± 1.8
	others	3.6 ± 2.5	3.9 ± 4.4	2.7 ± 0.5	6.0 ± 3.3	5.8 ± 2.0	7.0 ± 0.8
	n	*355*	*360*	*364*	*381*	*324*	*316*

Supplementary Table 6. Statistical AFM analysis of the assembly products of the full DNA origami at increasing magnesium ions concentrations, both in absence and presence of e(0) staples. The full structure was assembled in one-pot reaction either in absence of any edges, (e(-) construct) or in presence of the right- and left-side edges of type 0 (e(0) construct). Assembly conditions were 5 nM scaffold at -1°C/min and 50X staple strands. Assembly buffer was 1X TE containing different concentrations of magnesium ions, ranging from 2 mM to 20 mM. The end products of the thermal assembly were visualized at the AFM and counted manually. Standard deviations were obtained from three different images of the same sample for *n* distinct structures. One can note that, in the e(-) construct, whereas a low magnesium content favors the canonical shape (ca. 99%), high magnesium ions concentrations can lead to ca. a 30-fold increase in the fraction of isomerized forms, mainly at the level of the A domain. A similar trend, although to a less extent (only 3-fold increase in *iso* II shapes from 2 mM to 20 mM Mg^{2+} concentration), was observed in presence of the e(0) edges, meaning that mechanically-driven transformations may involve energy changes that are large enough to partially overwhelm those associated to chemical interactions at the crossovers.

		effective Mg concentration (mM)											
		**e(-)**						**e(0)**					
	isomers	*2*	*4*	*6*	*8*	*10*	*20*	*2*	*4*	*6*	*8*	*10*	*20*
5 nM, -1°C/min	*tot i-form*	0.6	1.1	3.8	6.0	10.4	18.0	25.4	59.7	59.1	64.5	68.5	62.4
	ccc	98.3	96.6	88.5	81.5	69.8	46.1	28.0	0.5	-	-	-	-
	icc	1.7	3.4	11.5	17.7	29.2	53.9	66.5	39.8	21.0	13.2	19.0	6.7
	iic	-	-	-	0.8	1.0	-	5.0	39.8	66.3	38.7	56.5	53.9
	iii	-	-	-	-	-	-	0.4	19.9	7.9	34.3	24.5	24.3
	others	-	-	-	-	-	-	-	-	4.7	13.7	1.6	15.0
	n	*357*	*499*	*625*	*249*	*202*	*321*	*211*	*211*	*190*	*204*	*184*	*193*

Supplementary Table 7. Statistical AFM analysis of the assembly products of the full e(-) DNA origami in absence or presence of e(0) staples in domain A. The full structure was assembled in one-pot reaction in the absence of any edges (e(-) construct). Identical solutions were prepared where pairs of adjacent e(0) staples have been added, which target the left-side of domain A, while keeping domain B and C unchanged. Staple pairs are indicated as e(1/2), e(3/4), e(5/6), e(7/8) and e(8/9), and refer to the positions on the left-side of domain A, going from the top to the bottom of the structure. The end products of the thermal assembly were visualized at the AFM and counted manually. Standard deviations were obtained from three different images of the same sample for *n* distinct structures. Assembly conditions were 2 nM scaffold at -1°C/min in 1X TEMg with 50X staple strands.

		domain A: edges e(-) → e(0)					
	isomers	e(-)	e(1/2)	e(3/4)	e(5/6)	e(7/8)	e(8/9)
2 nM, -1°C/min	*tot i-form*	13.3 ± 0.1	28.2 ± 2.6	30.4 ± 0.4	21.6 ± 0.2	18.1 ± 0	16.0 ± 0.5
	tot c-form	79.5 ± 0.1	70.9 ± 2.6	66.3 ± 0.4	72.5 ± 0.2	76.0 ± 1.0	81.9 ± 0.5
	ccc	53.7 ± 1.0	15.8 ± 3.4	5.9 ± 0.9	29.2 ± 2.8	39.9 ± 0.3	50.4 ± 1.7
	icc	38.3 ± 0.1	82.0 ± 5.5	90.5 ± 0.5	64.9 ± 0.6	54.2 ± 0.1	47.1 ± 0.7
	iic	0.8 ± 0	1.2 ± 1.2	0.4 ± 0.4	-	-	0.4 ± 0.4
	iii	-	-	-	-	-	-
	others	7.2 ± 1.2	0.9 ± 0.9	3.3 ± 1.0	5.9 ± 3.4	5.9 ± 0.3	2.1 ± 0.5
	n	*248*	*311*	*274*	*262*	*288*	*236*

Supplementary Table 8. Base stacking energies of the DNA origami domains in the two isomeric forms and in different edge designs. Values of base stacking energy were calculated as the sum of the stacking contributions from the two pairs of bases at each crossover, in both the *iso* I and *iso* II configuration. The addition of edges (e(0) construct) leads to an increase of base stacking energy in all domains, when compared to the values calculated in the absence of them (e(-) construct). Lack of an additional set of staples on the right side of each domain (e(--) construct) results, as expected, in a further decrease of base stacking energy. The difference in energy values between the *iso* II and *iso* I form (in bold), generally favours the latter over the former, for the reasons explained in the main text and Suppl. Fig. 25 and 26. Despite distinct sequence contents clearly result in different base stacking contributions, the values obtained by our calculations are not always consistent with the experimental data observed, suggesting either (i) a minor role of base stacking energy to folding or (ii) an inaccurate assessment of their actual contribution to it.

			edges design		
	isomers	**domain**	**e(0)**	**e(-)**	**e(--)**
base stacking energy (kcal/mol)	*iso* I	A	-141.1	-107.8	-95.1
		B	-141.3	-94.8	-74.6
		C	-129.8	-99.0	-80.9
	iso II	A	-173.7	-116.6	-104.3
		B	-163.8	-108.6	-95.1
		C	-143.9	-97.1	-79.4
	$\Delta(E_{II}-E_{I})$	A	**-32.6**	**-8.9**	**-9.2**
		B	**-22.4**	**-13.7**	**-20.4**
		C	**-14.2**	**+1.9**	**+1.5**

Supplementary Table 9. Statistical AFM analysis of the effect of fluorescent labelling on the degree of isomerization. The full DNA origami structure was assembled in one-pot reaction in the absence of any edges (e(-) construct). Identical solutions were prepared where the domain A contained two fluorescently labelled strands for performing orthogonal temperature-dependent FRET experiments. One FRET sample was designed to be sensitive to folding/unfolding of *iso* I only (D/A FRET I), the other FRET sample was instead designed to monitor the folding/unfolding of *iso* II only (D/A FRET II). The end products of the thermal assembly were visualized at the AFM and counted manually. Standard deviations were obtained from three different images of the same sample for *n* distinct structures. Assembly conditions were 50 nM scaffold at +/-1°C/min in 1X TEMg with 50X staple strands.

		e(-)		
	isomers	**unlabelled**	**D/A (FRET I)**	**D/A (FRET II)**
50 nM, -1°C/min	*tot i-form*	9.3	5.6	2.2
	ccc	68.3 ± 2.2	78.9 ± 2.3	91.5 ± 2.3
	cci	27.4 ± 2.1	16.7 ± 1.2	6.6 ± 3.0
	cii	-	-	-
	iii	-	-	-
	others	3.9 ± 1.2	4.4 ± 1.1	1.9 ± 1.2
	n	*423*	*365*	*364*

Supplementary Table 10. Thermodynamic analysis of the *iso* I and *iso* II isomers of domain A from the FRET thermal profiles at +/- 1°C/min. Values of melting temperatures were obtained from the first derivative of the E_{norm} vs T profiles (Suppl. Fig. 42). Activation energies relative to the cooling or melting process of both conformers could not be extracted by the thermal profiles of the samples because of lack of a sufficient number of data points around the melting temperature, which is necessary for a statistically relevant application of a linear fitting model.

		iso I		***Iso II***	
		cooling	*melting*	*cooling*	*melting*
		T_m (°C)	T_m (°C)	T_m (°C)	T_m (°C)
+/- 1°C/min	**e(-)**	61	60	63/58	64/59
	**e(0)**	-	-	64	66
	**e(1)**	-	-	63	64
	**e(2)**	61	60	63	64

Supplementary Note 1.

Kinetic analysis of the thermal process

Isoconversional (model-free) methods are used for determining the activation energy as a function of the reacted fraction without any previous assumption on the kinetic model fitted by the reaction. In particular, the Friedman-Ozawa isoconversional method[7,8] is a widely used differential method that, unlike conventional integral model-free methods, provides accurate values of activation energies even if the activation is a function of the reacted fraction.[9]

Let´s assume we have a reaction, which can be followed as the variation of a fraction α of a defined species during time. The reaction rate can be described by the following general equation:

$$v = \frac{d\alpha}{dt} = k \times f(\alpha) \qquad \text{Eq. 1}$$

where k is the rate constant and can be described by the Arrhenius equation (Eq. 2).

$$\frac{d\alpha}{dt} = Ae^{-\frac{E_{act}}{RT}} \times f(\alpha) \qquad \text{Eq. 2}$$

in which A is the Arrhenius pre-exponential factor, R is the gas constant and E_{act} is the activation energy of the reaction under study. Equation 2 is a general expression that describes the relationship among the reaction rate, the reacted fraction and temperature independently of the thermal pathway used for recording the experimental data. In case the experimental data are recorded at a constant heating rate (β):

$$\beta = \frac{dT}{dt}$$

Eq. (2) can be written as follows

$$\frac{d\alpha}{dT} = \frac{A}{\beta}e^{-\frac{E_{act}}{RT}} \times f(\alpha) \qquad \text{Eq. 3}$$

Here the reaction is recorded as a function of time, which in turn corresponds to a linear increase (or decrease) in temperature.

The logarithmic form of the general kinetic Eq. (2) can be then expressed as follows:

$$ln\left(\frac{d\alpha/dt}{f(\alpha)}\right) = \ln(A) - \frac{E_{act}}{RT} \qquad \text{Eq. 4}$$

If the proper $f(\alpha)$ is considered for the analysis, the plot of the left-hand side of the equation versus the inverse of the temperature will yield a straight line, whose slope leads to the activation energy and the intercept leads to the pre-exponential factor. As no assumption regarding the thermal pathway is made in Eq. (4), the kinetic parameters obtained should be independent of the model used to describe the reaction. The challenge is to determine the function $f(\alpha)$. Most kinetic equations described in the literature have been developed assuming idealized physical conditions; however, these are not always satisfied in real systems, making some models unsuitable to describe the thermal process under study.

To overcome this problem, a new procedure has been recently introduced, which describes the function $f(\alpha)$ with the following general expression:[10]

$$f(\alpha) = c(1-\alpha)^n\alpha^m \qquad \text{Eq. 5}$$

This equation is a modified form of the Sestak-Berggren empirical equation[11] and has been shown to fit almost every function by merely adjusting the parameters *c*, *n* and *m.* Thus, substituting in Eq. (4), one obtains a general expression that covers the most common physical models and their possible deviations from ideal conditions.

$$ln\left(\frac{d\alpha/dt}{(1-\alpha)^n\alpha^m}\right) = \ln(cA) - \frac{E_{act}}{RT} \qquad \text{Eq. 6}$$

Despite this method has been originally developed for the analysis of solid-state thermal processes, the theoretical background is of general applicability and can be employed for any set of experimental data obtained under any heating schedule.

We therefore developed a software suite encoded in R program language to find out the parameters *n* and *m* that yield the best linear correlation, from which the corresponding values of E_{act} can be calculated. We used the open source software RStudio to access and analyze our data from R.

The good match between the values obtained applying this model-free method and the two-state model approximation to the thermal profiles of the e(2) construct in the *iso* I form confirmed the validity of the approach used.

Values of n and m found for best fitting of the *iso* I and *iso* II curves were consistently equal in all designs analyzed, supporting the validity of the approach used to describe the thermal process. In particular, *iso* I thermal profiles, associated to both the heating and cooling process, were described by $n = 0.7 \pm 0.2$ and $m = 2.5 \pm 0.3$. Thermal profiles of the *iso* II shapes were instead described by $n = 0.3 \pm 0.1$ and $m = 1.9 \pm 0.3$.

Supplementary Methods

Linearization of the M13mp18 scaffold

(This procedure was used to obtain the data reported in Suppl. Fig. 46)

Annealing. 10 µL of M13mp18 (1.57 µg/µL) was mixed with 25 µL of the oligonucleotide sequence complementary to the HindIII recognition site (5' to 3': AACGACGGCCAGTGCCAAGCTTGCATGCCTGCAGGTCGACTC) and 65 µL TEMg buffer to reach a final volume of 100 µL. The mixture was incubated for 5 minutes at 95°C and subsequently cooled to 10°C at a rate of -1°C/min. Two sample were prepared and run in parallel.

Restriction digest. 50 µL of the annealed sample was mixed with 10 µL CutSmart Buffer (New England Biolabs), 1 µL HindIII-HF restriction enzyme (New England Biolabs) and 39 µL aqua dest. in order to gain a final volume of 100 µL. The mix was incubated at 37°C for 1 hour. Enzymes were inactivated at 80°C for 20 minutes and finally the mix was cooled down to 10°C. Four digest solutions were prepared and run in parallel.

Purification of digests. All four digests were purified by ultracentrifugation, using 50 kDa Amicon Ultracentrifugal Filter Units. The filters were washed beforehand by applying 300 µL of aqua dest. followed by centrifugation at 15.000 rcf for 2 minutes. Digests were pooled, applied to the filter unit and centrifuged at 4.500 rcf for 5 minutes. The filters were then washed twice with 300 µL aqua dest., placed upside down onto a test tube and centrifuged at 2000 rcf for 2 minutes. Final concentration was determined using a DS-11 Spectrophotometer.

Exonuclease digest. To test whether the linearization process did work, a small sample was taken from the purified digest and treated with Exonuclease I (New England Biolabs). For this purpose, about 1 µg of purified scaffold was mixed with 5 µL exonuclease I buffer (New England Biolabs), 1 µL Exonuclease I and filled up to a final volume of 50 µL with aqua dest. The mix was incubated at 37°C for 1 hour. Enzymes were inactivated at 80°C for 20 minutes and finally the mix was cooled down to 10°C.

Supplementary References

1. Pan, K. et al. Lattice-free prediction of three-dimensional structure of programmed DNA assemblies. *Nat Commun* **5**, 5578 (2014).
2. Woo, S. & Rothemund, P.W. Programmable molecular recognition based on the geometry of DNA nanostructures. *Nat Chem* **3**, 620-627 (2011).
3. Zuker, M. Mfold web server for nucleic acid folding and hybridization prediction. *Nucleic Acids Res* **31**, 3406-3415 (2003).
4. Yakovchuk, P., Protozanova, E. & Frank-Kamenetskii, M.D. Base-stacking and base-pairing contributions into thermal stability of the DNA double helix. *Nucleic Acids Res* **34**, 564-574 (2006).
5. Nash, J. DNA Origami Visualization Tools. https://nanohub.org (2017).
6. Protozanova, E., Yakovchuk, P. & Frank-Kamenetskii, M.D. Stacked-unstacked equilibrium at the nick site of DNA. *J Mol Biol* **342**, 775-785 (2004).
7. Friedman, H.L. Kinetics of thermal degradation of char-forming plastics from thermogravimetry. Application to phenolic plastic. *J Polym Sci C Polym. Symp* **6PC**, 183-195 (1964).
8. Ozawa, T. Kinetic analysis by repeated temperature scanning. Part 1. Theory and methods. *Thermochimica Acta* **356**, 173-180 (2000).
9. Criado, J.M., Sanchez-Jimenez, P.E. & Perez-Maqueda, L.A. Critical study of the isoconversional methods of kinetic analysis. *J Therm Anal Calorim* **92**, 199-203 (2008).
10. Perez-Maqueda, L.A., Criado, J.M., Gotor, F.J. & Malek, J. Advantages of combined kinetic analysis of experimental data obtained under any heating profile. *J Phys Chem A* **106**, 2862-2868 (2002).
11. Sestak, J. & Berggren, G. Study of the kinetics of the mechanism of solid-state reactions at increased temperature. *Thermochim Acta* **3**, 1-12 (1971).
