# Supplementary material for: Sites of high local frustration in DNA origami
Source: Nat Commun. 2019 Mar 5;10:1061. doi: 10.1038/s41467-019-09002-6 (PMC6400978; doi:10.1038/s41467-019-09002-6)
Supplement: Supplementary file 3 — Description of Additional Supplementary Files [file 41467_2019_9002_MOESM3_ESM.pdf]

Description of Additional Supplementary Files

File Name: Supplementary Data 1

Description: Full list of DNA sequences used for the self-assembly of the DNA origami structures reported in this work. Sequence sets are highlighted in different colors.
